# Miniature Short Hairpin RNA Screens to Characterize Antiproliferative Drugs

**DOI:** 10.1534/g3.113.006437

**Published:** 2013-08-01

**Authors:** Saranya Kittanakom, Anthony Arnoldo, Kevin R. Brown, Iain Wallace, Tada Kunavisarut, Dax Torti, Lawrence E. Heisler, Anuradha Surendra, Jason Moffat, Guri Giaever, Corey Nislow

**Affiliations:** * Department of Molecular Genetics, University of Toronto, Toronto, Ontario M5S 3E1, Canada; † Banting and Best Department of Medical Research, University of Toronto, Toronto, Ontario M5S 3E1, Canada; ‡ Terrence Donnelly Centre for Cellular and Biomolecular Research, University of Toronto, Toronto, Ontario M5S 3E1, Canada; ** Donnelly Sequencing Center, University of Toronto, Toronto, Ontario M5S 3E1, Canada; § Division of Endocrinology and Metabolism 73170, Department of Medicine, Faculty of Medicine, Siriraj Hospital, Mahidol University, Thailand; †† Department of Pharmaceutical Sciences, University of Toronto, Toronto, Ontario M5S 3M2, Canada; ‡‡ Department of Pharmaceutical Sciences, University of British Columbia, Vancouver, BC, V6T1Z3, Canada

**Keywords:** shRNA screening, synthetic lethality, next-generation sequencing

## Abstract

The application of new proteomics and genomics technologies support a view in which few drugs act solely by inhibiting a single cellular target. Indeed, drug activity is modulated by complex, often incompletely understood cellular mechanisms. Therefore, efforts to decipher mode of action through genetic perturbation such as RNAi typically yields “hits” that fall into several categories. Of particular interest to the present study, we aimed to characterize secondary activities of drugs on cells. Inhibiting a known target can result in clinically relevant synthetic phenotypes. In one scenario, drug perturbation could, for example, improperly activate a protein that normally inhibits a particular kinase. In other cases, additional, lower affinity targets can be inhibited as in the example of inhibition of c-Kit observed in Bcr-Abl−positive cells treated with Gleevec. Drug transport and metabolism also play an important role in the way any chemicals act within the cells. Finally, RNAi *per se* can also affect cell fitness by more general off-target effects, *e.g.*, via the modulation of apoptosis or DNA damage repair. Regardless of the root cause of these unwanted effects, understanding the scope of a drug’s activity and polypharmacology is essential for better understanding its mechanism(s) of action, and such information can guide development of improved therapies. We describe a rapid, cost-effective approach to characterize primary and secondary effects of small-molecules by using small-scale libraries of virally integrated short hairpin RNAs. We demonstrate this principle using a “minipool” composed of shRNAs that target the genes encoding the reported protein targets of approved drugs. Among the 28 known reported drug−target pairs, we successfully identify 40% of the targets described in the literature and uncover several unanticipated drug−target interactions based on drug-induced synthetic lethality. We provide a detailed protocol for performing such screens and for analyzing the data. This cost-effective approach to mammalian knockdown screens, combined with the increasing maturation of RNAi technology will expand the accessibility of similar approaches in academic settings.

Despite enormous spending increases in pharmaceutical research and development, the number of new drugs approved each year has remained nearly constant during the past 60 years (Munos 2009). Among the many contributing factors is the lack of novel “credentialed” targets with strong disease associations (Rask-Andersen *et al.* 2011). An experimental approach that simultaneously identifies such novel targets along with potential inhibitors would be quite valuable. Furthermore, better understanding of a drug’s primary mechanism of action and potential polypharmacological effects can help uncover new therapeutic applications (Roth *et al.* 2004; Boran and Iyengar 2010; Kneller 2010; Knight *et al.* 2010; Morrow *et al.* 2010). During the past decade, our group and others have made extensive use of parallel screening of yeast deletion mutants for drug target identification (Giaever *et al.* 1999; Skrtic *et al.* 2011), and here we aim to provide an analogous method that combines reverse genetics in human cells with drug-induced synthetic lethality. To date, the infrastructure cost and resources required to support genome-wide, human reverse genetic screens have limited the access of many labs to this powerful technology.

RNA interference is a reliable and efficient approach to modulate gene expression in mammalian cells. It is also a powerful technique for identifying putative drug targets by knocking down mRNA, subsequently reducing protein expression and observing the resulting cell fitness in the presence of drug (Knight *et al.* 2010). For example, when dihydrofolate reductase (*i.e.*, DHFR) gene expression is reduced by short hairpin RNA (shRNA) knock-down, cells become hypersensitive to methotrexate treatment (Giaever *et al.* 2004). Stable and persistent gene knockdown has become feasible by integrating shRNAs with lentivirus as the delivery system; genome-scale, cell-based RNA interference (RNAi) screens are now performed in many larger laboratories and core facilities (Bommi-Reddy *et al.* 2008; Duan *et al.* 2010; Smogorzewska *et al.* 2010). However, analysis of the data from such screens is a challenge because most screens include multiple shRNAs per gene but rarely do all create the same level of knockdown, even in the same genetic background. This challenge is magnified as the size of the RNAi pool increases. Finally, the variety of different experimental designs and readout methods (*e.g.*, microarrays *vs.* sequencing) comprise additional variables.

To develop a straightforward, reproducible screening platform for drug evaluation, we designed a mini-pool shRNA library against known human therapeutic drug targets and developed a set of extensible protocols for their use and analysis. We focused our effort on FDA-approved drugs to benchmark our method and to potentially gain insight into how such drugs might be repurposed toward new targets. Accordingly, we generated a shRNA library to target genes that encode known targets, reasoning that any additional activities of the drugs will manifest as deviations from expectation. Given the library's small size, the screen is readily performed in reduced culture volumes, decreasing the amount of drug consumed, increasing the number of compounds that can be screened, and keeping overall cost low. Although the number of protein targets and drugs tested here is modest (368 and 50, respectively) our compilation of experimental profiles provides a foundation for future clustering and pattern matching informatics studies that can be applied to less well-characterized compounds. We expect that these results will illuminate some of the biology that underlies the enormous variability in patient drug response and that this simple robust protocol can be adopted and adapted for different cellular pathways.

## Materials and Methods

### Cell line and growth condition

A549 cells (human lung adenocarinoma) were obtained from ATCC (http://www.atcc.org) and maintained in Dulbecco’s Modified Eagle Medium (DMEM) + 10% fetal bovine serum (FBS) + penicillin/streptomycin (P/S) and incubated at 37° and 5% CO_2_.

### shRNA minipool library

Three hairpins were selected for each of 368 human genes from The RNAi Consortium (TRC) lentiviral libraries (http://www.broadinstitute.org/rnai/trc; Supporting Information, Figure S1). Hairpins were selected based on the reported knockdown efficiency, as measured by real-time polymerase chain reaction (PCR) by TRC (Table S7). The resulting pool contains 1098 shRNA lentivirus plasmids that target 368 human genes. Equal amounts of plasmid DNA were combined, and the pooled shRNA plasmids, including lentivirus packaging and viral envelope plasmids, were mixed and transfected into HEK293T packaging cells using FuGENE reagent (Roche). Forty-eight hours after transfection, media containing lentivirus shRNA particles were collected, aliquoted and stored at −80° as described (http://www.broadinstitute.org/rnai/public/resources/protocols).

### Minipool infection of A549 cells

A549 cells (3 × 10^7^) were infected with the lentiviral shRNA minipool at a multiplicity of infection of 0.3−0.4. After 2 d of selection in 2 μg/mL puromycin-containing medium to eliminate uninfected cells, puromycin-resistant A549 cells were amplified for an additional 2 d, resulting in a total of 4 × 10^7^ cells. The transduced A549 cells were split into aliquots of 1.5 × 10^6^ cells each and frozen for future screens. Frozen aliquots of the A549 with the integrated shRNA minipool collection are available upon request.

### Dose determination for growth inhibition

Infected A549 cells were seeded in 96-well plates at 2200 cells/well in 200 μL of medium. Drugs were serially diluted first in dimethyl sulfoxide (DMSO) then in RPMI, with the final DMSO concentration not exceeding 1%. After 72 hr of incubation, cell viability was measured with a sulforhodamine B (SRB) viability assay (Vichai and Kirtikara 2006). Briefly, cells were fixed by addition of trichloroacetic acid to 3.3% w/v final concentration, incubated at 4° for 30 min, then washed gently five times with running tap water. Fixed precipitates were dried and 50 μL of 0.4% w/v of SRB dissolved in 1% acetic acid was added to each well and incubated at room temperature for 30 min on a rocking platform. Unbound SRB was removed by four washes with 1% acetic acid. The bound dye was solubilized by adding 150 μL of 10 mM Tris base (pH 10.5). After 10 min, free dye concentration was determined using a microplate reader at a wavelength of 510 nm (BioTek Synergy 2) within 1 hr. Background subtraction was based on wells incubated in the absence of cells. Percentage growth inhibition was calculated by dividing the absorbance obtained from the cells treated with drug by the absorbance of the cells cultured in presence of vehicle alone (100% growth control). Results obtained from 96-well plate growth were used as a guide to test a narrow range of drugs in 6-well plates, the format used for the screens. For each drug, a concentration that corresponded to 25% growth inhibition (IC_25_) was used for subsequent minipool screens (Table 1).

**Table 1:** Compounds used in the screens

Class	Average IC_25_		Compound	Screening concentration, μM	Clinical Application
Antiproliferative	8.70 ± 19.20 μM	3.16 ± 4.85 μM (excluding outliers)	Hydroxy urea	90	Antineoplastic
			Etoposide	0.35	Antineoplastic
			Camptothecin (nm)	5	Antineoplastic
			Doxorubicin (nm)	6	Antineoplastic
			Vincristine (nm)	6.5	Antineoplastic
			Amsacrine	12.5	Antineoplastic
			Methotrexate	0.03	Antineoplastic
			Taxol (nm)	1	Antineoplastic
			Gossypol	5	Antineoplastic
			Methyl methanesulfonate (%)	0.0012	Antineoplastic
			Vorinostat	1.25	Antineoplastic
			Gefitinib	4	Antineoplastic
			Mitomycin C	9	Antineoplastic
			Imatinib	10	Antineoplastic
			Marimastat (BB-2516)	16	Antineoplastic
			Digoxin (nm)	12	Heart treatment
			Cyclosporin A	0.45	Immunosuppressive
			Mycophenolic acid	0.4	Immunosuppressive
			Rapamycin (nm)	1	Immunosuppressive
			Tacrolimus	22	Immunosuppressive
			Rotenone	0.1	Insecticide, and pesticide
			Roscovitine	3	Treatment of nonsmall cell lung cancer, leukemia, HIV infection, herpes simplex infection
			Mitaplatin	1	Antineoplastic
			Retinoic acid	25	Antineoplastic
Nonantiproliferative	162.66 ± 222.46 μM	80.87 ± 75.20 μM (excluding outliers)	Racecadotril	90	Antidiarrheal
			Artemisinin	22.5	Anti-infective
			Sulfasalazine	900	Anti-inflammatory
			Indomethacin	95	Anti-inflammatory (NSAID)
			Naproxen	96	Anti-inflammatory (NSAID)
			Ibuprofen	450	Anti-inflammatory (NSAID)
			Salicylate	700	Anti-inflammatory (NSAID)
			Verapamil	35	Antiarrhythmic, angina, hypertension
			Tigecycline	50	Antibiotic
			Erythromycin	200	Antibiotic
			Warfarin	120	Anticoagulant
			Metformin	85	Antihyperglycemic
			Orlistat	11.2	Antilipemic
			Lovastatin	16.5	Antilipemic
			Trifluoperazine	10	Antipsychotic
			Haloperidol	17.5	Antipsychotic
			Clozapine	16.5	Antipsychotic
			Methimazole	400	Antithyroid
			Isoproterenol	27.5	Asthma and bronchospasm
			Aminophylline	225	Asthma and bronchospasm
			Propanolol	33	Bronchospasm and heart treatment
			Mancozeb	42.5	Fungicide
			Allopurinol	250	Hyperuricemia treatment
			Sildenafil	46	Pulmonary hypertension
			Naltrexone	80	Treatment of alcohol dependence
			Mptp	210	Neurotoxin, Parkinson disease

NSAID, nonsteroidal anti-inflammatory drug.

### Minipool drug screens in infected A549 cells

Infected A549 cells were seeded in six-well plates at 1.65 × 10^5^ cells/well in 2 mL of DMEM + FBS + P/S such that each hairpin was represented at least 125 times per well. The cells from the first generation of growth were saved as the “T0” sample and stored at −80°. Two hours after seeding, drug (at IC_25_) was added to fresh cells. Cells were incubated for 72 hr and trypsinized. Then, 1.65 × 10^5^ cells/well were reseeded in 2 mL of DMEM + FBS + P/S + drug, whereas 5−8 × 10^5^ cells were collected and stored at −80° for subsequent genomic DNA extraction. Each screen comprised seven consecutive 3 d time points and was performed in triplicate.

### Genomic DNA extraction and shRNA amplification (half-hairpin barcodes)

The genomic DNA of each individual sample was extracted using a QiaExtractor DNA robot (QIAGEN) in a 96-well format according to manufacturer’s instructions. DNA was normalized such that, on average 1 ug was obtained and 50% was used in each barcode PCR. PCR amplification of shRNA was performed using indexed Illumina PCR primers to incorporate both the Illumina adapter sequences and indexing sequences, with Platinum Pfx polymerase kit (Invitrogen) as previously described (Ketela *et al.* 2011). The PCR product was verified on a 2% agarose gel to confirm that there was a preponderance of a ~234-bp PCR product (178 bp for hairpin + 48 bp for Illumina adaptor + and 8 nt for barcode). PCR products were pooled (30 μL for each sample) and purified using a QIAGEN PCR purification kit. Purified pooled samples were separated by agarose gel electrophoresis and the 234-bp product was excised. MinElute PCR purification was used to extract DNA from the gel slice before sequencing on an Illumina HiSeq2000.

### Illumina next-generation sequencing

Normalized DNA template was quantified by real-time PCR as described previously (Ammar *et al.* 2009; Smith *et al.* 2009), diluted to 8pM, and clusters were generated on a single-read flow cell. Samples were divided into two batches, each consisting of 25 drug treatments plus vehicle control, at eight time points each, in triplicate. An 8-mer index sequence unique to each sample was incorporated along with the Illumina adapters before it was clustered onto the flowcell to allow multiplexing of 96 samples within each flowcell lane. The hairpin sequence was generated using the sequence primer 5′-GATTTCTTGGCTTTATATATCTTGTGGAAAGGACGAAACACCGG, and collecting 20 cycles. A second read of eight cycles for the sample index was generated using the primer 5′-GAATTCTCGACCTCGAGACAAATGGCAGTATTCATCCACA. The median reads generated per sample was 720,918 (interquartile range 532,435), which corresponds to an average count of ~656 reads for each of the 1098 hairpins in the pool. Hairpins were matched to sequence reads, using custom Perl scripts, allowing up to two mismatches per alignment. The median percentage of reads assigned to a hairpin across all samples was 97%. The actual range of counts assigned to each hairpin varied between 0 and 17,000.

### Data analysis

Raw read counts were shifted up by 100 to remove zero counts and converted to log_2_. Replicate screens were normalized by cyclic loess using the Bioconductor package *limma* (v.3.12.0), followed by taking the antilog of the counts. To assess the combined impact of shRNA knock-down and drug treatment on cell growth, a linear model was fit to the observed triplicate data for each hairpin as follows. Briefly, we fit a generalized linear model with Poisson distribution and log link function using the *glm* function in R (v.2.15.0). Normalized read counts were treated as the response variable, number of population doublings as the first predictor variable, and drug treatment and doublings as an interaction term. This scoring approach assumes (correctly) a common intercept on the *y*-axis (starting read counts) for both the vehicle control and the drug treatment, and allows for missing data when necessary. Hairpins were ranked according to the fitted interaction parameter as a measure of the effect of drug treatment while controlling for the effect of the hairpin treatment. Hairpins with interaction values that are 2 SD from the screen mean (those synthetic lethal or those rescuing the drug toxicity) were considered as primary hits.

### Detection of cell death by flow cytometry

Specific target genes in A549 cells were silenced by lentiviral shRNA as described previously. Three days after drug treatment, cells were harvested, washed, and stained with fluorescein isothiocyanate−conjugated annexin V and propidium iodide according to manufacturer’s instructions (BD 556419). Cells undergoing apoptosis were detected by phosphatidylserine externalization (annexin V-positive, propidium iodide-negative) using two-color flow cytometry analysis on a BD FACS Calibur. A step-by-step protocol is provided as an appendix (File S1).

## Results and Discussion

### Computational selection of protein targets and selection of diverse test drugs

The genes comprising our shRNA minipool library were selected on the basis of their previous characterization as human drug targets, *i.e.*, we excluded bacterial, viral, fungal, or other targets. The list was further filtered to include only those targets that were characterized as being inhibited by at least one compound present in the collection of 640 Food and Drug Administration−approved drugs from Biomol (http://www.enzolifesciences.com/BML-2841/fda-approved-drug-library/), ChEMBL (http://www.ebi.ac.uk/chembldb/), and MATADOR (http://matador.embl.de). The resulting 368 human drug targets were then grouped according to their Gene Ontology designation. Gene Ontology analysis showed that the final list of targets belong to diverse pathways including signaling, protein/DNA metabolism, transport, receptor, cell cycle, DNA damage, and repair (Figure S1).

We prioritized 50 drugs of the 640 present in the Biomol FDA library for our screen against the mini-pool library. For the purposes of this study, we divided these drugs into two classes (antiproliferatives and nonantiproliferatives). Together this set covers a broad range of therapeutic indications, including immunosuppressive agents, antipsychotic, anti-cholesterol, anticoagulant, anti-inflammatory, antidiabetic, and cancer chemotherapeutics (Table 1). For each drug, dose-curve responses were established in A549 (nonsmall cell lung carcinoma) cells to establish a screening concentration at which each compound manifests an IC_25_ (as indicated in Table 1). Among the FDA-approved drugs, the majority have a defined mode of action even though a significant number of the drugs tested target biological pathways rather than protein targets (*e.g.*, MMS and mitomycin C, which both target DNA-related processes).

### Overview of the minipool screen

To develop these screens, we used A549 cells because this lung cancer cell line is commonly used in large-scale cell-based assays (Bai *et al.* 2006; Ji *et al.* 2007; Chang *et al.* 2008). Many of the reasons are practical: they can be maintained in standard media, have a fast doubling time (about 22 hr), rapidly adhere after seeding, and are easily infected with lentivirus. They are also amenable to immunocytochemical analysis, and dead cells are easily eliminated during the course of the drug treatment as they detach from the surface and can be removed by media exchange. Of course, this particular cell line is not ideal for all purposes; like most cultured cells, it is defective in certain pathways and deregulated for others. Therefore, mutations found in the cell line might influence its response to the drug treatment (Barretina *et al.* 2012). Nonetheless, its positive features outweighed its negatives and provided a starting point. Our preliminary screens indicated that drug treatment at IC_25_ was a suitable dose in that it maintained drug pressure while exhibiting minimal variation in inhibition after each splitting during the 21 d screens. This value is similar to the IC_15-30_ used in many large-scale yeast screens (Giaever *et al.* 2004; Lum *et al.* 2004). The small size of the shRNA minipool provides the flexibility to transfer the pool to diverse cell lines to better match the cell line to the nature of the perturbation that is to be tested. Further, the ability to infect with virus and then freeze “screen-sized” aliquots of cells eliminates the need for reinfection before every experiment and minimizes this potential variable.

To test whether the freezing and thawing affected the representation of our hairpin collection in the A549 cells, we compared two sets of infected cells. The first set contains cells collected immediately after the original infection (“mother” population). The second set represents cells from two stock vials used in all our screens, obtained after infection, puromycin selection, amplification, and one freeze-thaw cycle (“daughter” populations 1 and 2). For each sample, genomic DNA was extracted from 1 × 10^6^ cells and the shRNAs amplified with PCR and sequenced with the use of Illumina HiSequation 2000. A high correlation (r = 0.981) in the sequence count number for each hairpin was found between daughter populations (Figure S2A, right panel) but more importantly, a very high correlation (r = 0.947 and r = 0.932) was also found between the mother and both daughter populations (Figure S2A, left and middle panels). Additionally, a closer analysis revealed that only 25 shRNAs (of 1098) were missing from our collection and that most of these were lost immediately after the initial infection step (Figure S2B). Thus, freezing aliquots after the initial infection provides a uniform source of cells for screening. These results encouraged us to screen our minipool of 1098 shRNAs targeting 368 human genes, against 50 selected drugs at IC values between 20 and 30%.

To estimate the number of cytotoxic hairpins in the A549 cell background, we calculated the hairpin representation after 12 and 21 doublings, when the cells were cultured in the presence of DMSO, methanol, or a no-vehicle control. We first compared how well different screens correlated with one another. As anticipated, greater correlations were observed after 12 doublings compared to 21 doublings for all three culture conditions, reflecting a continuing dynamic loss and amplification of shRNAs overtime. A good correlation (0.72 < r < 0.85) was observed between different doubling times compared with T0, attesting to the moderate level of toxicity conferred by the expression of hairpins (Figure S3). To directly address what fraction of the minipool library was depleted in the absence of drug, we analyzed the sequence count numbers for each hairpin and found that less than 7% of the minipool library (73/1098 shRNAs) was toxic in A549s (Figure S4).

**Figure 1:**
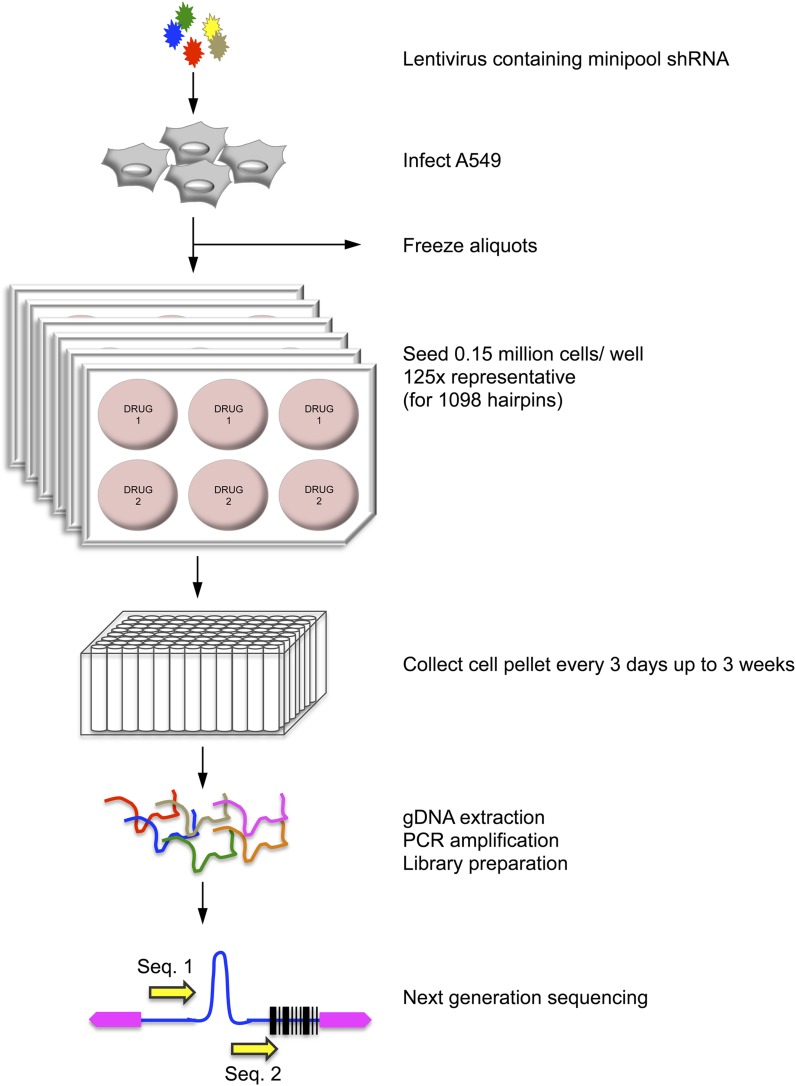
Schematic of drug screen. A549 cells were infected with a pool of lentivirus containing 1098 shRNAs. Two days after puromycin selection, transduced A549s were amplified, aliquoted, and frozen for further screens. Cells were seeded in six-well culture plates (0.150 million cells/ well, 150x hairpin representation) and treated with the drug of interest in triplicate. The cells were split and harvested every 3 d for 21 d. Genomic DNA was extracted using high throughput QiaExtractor (QIAGEN) in a 96-well format. shRNAs were amplified by PCR, and the samples were indexed for next-generation sequencing.

The small size of the minipool allowed us to perform screens in reduced culture volumes and in triplicate (one screen per well, two drugs per six-well plate). This format allowed a single person to test 50 drugs in a few weeks. After drug treatment, cells were collected every 3 d and maintained in culture in drug over 21 d, being split every third day. Cell counts were obtained at each subculture step. Once the final time point was collected, the shRNAs from each time point were amplified via PCR from gDNA as described (Ketela *et al.* 2011) and sequenced using an Illumina HiSequation 2000 (Figure 1). A total of 96 samples (30 drug screens in triplicate with two vehicle controls) were combined in the same lane for massively parallel sequencing. Sequence reads for individual screens were demultiplexed by virtue of their unique index sequences (Smith *et al.* 2010).

**Figure 2:**
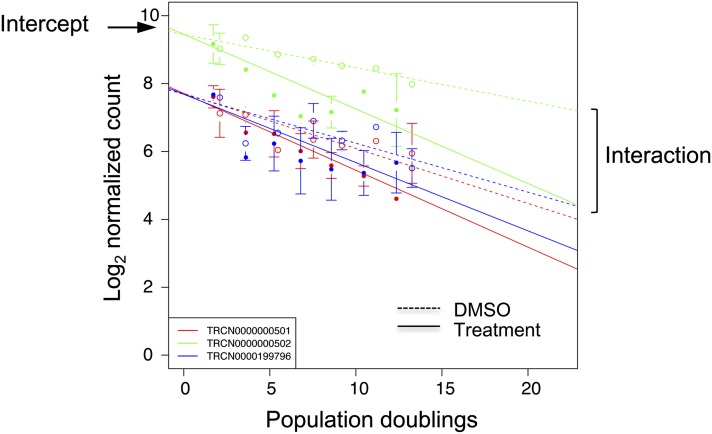
Generalized linear model fitting. Log-linear trends with a negative slope indicate the depletion of cells containing specific shRNAs over time. The dashed line shows the vehicle control (DMSO); the solid line indicates the drug treatment. Error bars represent the SD for triplicate drug treatments. An interaction term from the model fits was used to quantify the difference in slopes between the cells cultivated with or without drug while controlling for the effect of the hairpins.

For each drug, the triplicate screens showed a high correlation at each time point (*e.g.*, r ≥ 0.8, day 21, Figure S5). We quantified the abundance of each shRNA in the presence or absence of drug. A linear fit was applied to each of the three shRNAs for each gene (see the section *Materials and Methods* and Figure 2). A typical profile for a drug and the shRNA(s) directed against its mRNA is characterized by a relative decrease in sequence counts over time in presence of drug. These linear plots depict the log_2_-transformed sequence counts on the Y-axis and the number of cell doublings on the X-axis. The analysis is computed such that DMSO and drug share the common origin of shRNA sequence counts (the intercept). The difference between DMSO controls (dashed line) and drug treatment (solid line) is designated as the “interaction value,” a term that reflects the abundance of shRNAs in the population during the course of drug treatment. Any shRNA that renders a cell hypersensitive is characterized by a negative interaction value. Based on their interaction values (Table S2) and their linear plots, a list of potential synthetic lethal genes for each of the 50 tested drugs was then compiled (Table S3).

### Understanding drug mechanism

Drugs were classified according to their known antiproliferative and nonantiproliferative properties in cultured human cells. As anticipated, our assay was more sensitive for antiproliferative compounds (average IC_25_ = 8.70 μM) compared with the nonantiproliferative drugs (average IC_25_ = 162.66 μM; Table 1). In addition, the trend for the calculated IC_25_s correlated well to the reported experimental GI_50_ values (Table S1), further validating the doses we selected and highlighting that A549 is an appropriate cell line to test and validate our initial pipeline.

Among the 50 screened drugs, 28 have targets that are reported in the literature. We identified the targets for 11 of these drugs (40%) in the top hits from our screens (Table S4). A total of 56% of targets were identified using antiproliferative compounds, whereas only 17% of reported targets were identified with nonantiproliferatives. This enrichment for antiproliferatives reflects the nature of the screen and the growth readout which we used. By acting on a target whose activity is crucial for the cell survival, antiproliferative agents induce toxicity at low concentrations. In contrast, nonantiproliferative agents modulate targets whose function is not necessarily essential for cell growth, and, consequently, greater concentrations are required to induce a detectable growth defect. The results also highlight the need to tailor the assay for nonantiproliferative drugs by, for instance, selecting a non growth-based assay. Despite a greater risk of false-positive results that could arise from using relatively high doses of drug, the study of nonantiproliferative compounds can also prove useful in understanding secondary activities (Ericson *et al.* 2008). The normalized sequence count number for individual hairpin results from all 50 screens is provided as additional files (Table S5 and Table S6).

**Figure 3:**
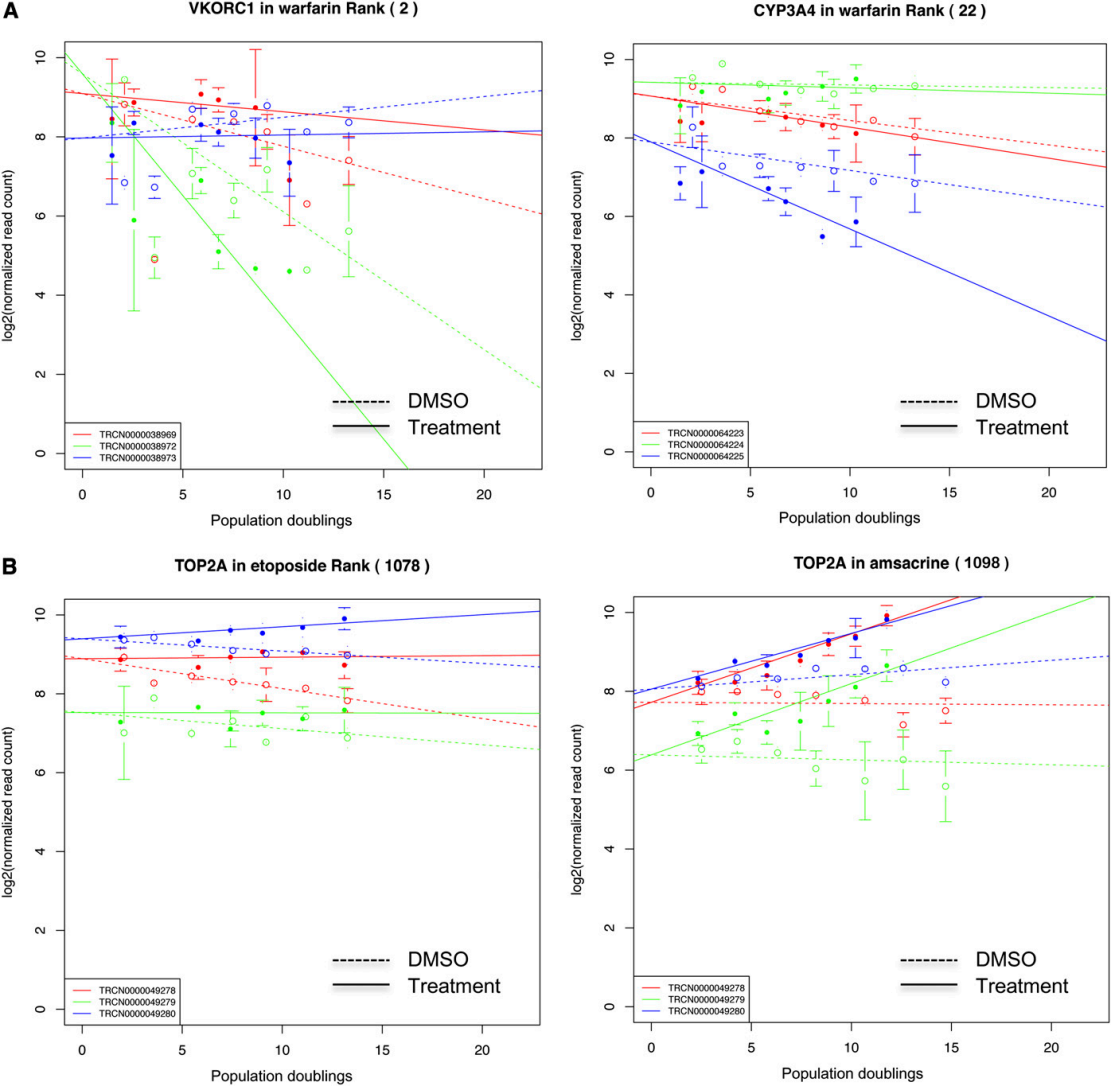
Proof-of-principle drug-target pairs. (A) When silenced, VKORC1 (left panel) and CYP3A4 (right panel), decreased cell survival during warfarin treatment. (B) Knock-down of topoisomerase II alpha (TOP2A) by three individual shRNAs conferred resistance to both etoposide (left panel) and amsacrine (right panel) treatment in A549 cells.

To demonstrate the utility of our approach, we highlight several screens from our compendium of 50 drugs (Table 1). Of particular interest, we selected three well-characterized inhibitors to validate the platform. The screen with warfarin, an anticoagulant used to prevent blood clots by inhibiting protein vitamin K epoxide reductase complex subunit 1 (VKORC1), revealed VKORC1 as one of the top hits (ranked 2 of 1098 with 2 of 3 shRNAs; Figure 3A, left panel) (Suarez-Kurtz *et al.* 2009). CYP3A4 (cytochrome P450 3A4), an essential protein in warfarin metabolism, was ranked 22 (2 of 3 hairpins), consistent with the essential role of metabolism with this drugs mechanism (Figure 3A, right panel) (Dandara *et al.* 2011; Zhong *et al.* 2011, 2012). The topoisomerase II inhibitors, etoposide and amsacrine, exert their cytotoxic effects by binding to DNA and forming a ternary complex with topoisomerase II, inhibiting DNA relaxation, inducing the formation of DNA breaks and resulting in cell death. Depleting topoisomerase II from cells by shRNA knockdown should prevent or reduce the formation of the toxic ternary complex and render these cells resistant to both of these agents. As shown in Figure 3B, all three hairpins targeting topoisomerase II alpha enhanced survival of A549 to either etoposide (left panel: rank 1078/1098) or amsacrine (right panel: rank 1098/1098) compared with DMSO (Koshiyama *et al.* 2001; Chen *et al.* 2005).

Having calibrated our screen and analysis to detect both drug sensitivity and resistance, we used the same approach to study drug mode-of-action for the 50 drugs summarized in Table 1. Below, we highlight the result from experiments with metformin (a diabetes treatment with a characterized target) and gossypol (an agent with an uncharacterized mechanism).

### Metformin

Metformin is an oral diabetic drug that decreases insulin resistance and is prescribed as a first-line treatment according to most type 2 diabetes mellitus guidelines (Inzucchi *et al.* 2012). Metformin activates the AMP-activated protein kinase AMPK, a liver enzyme that plays an important role in insulin signaling, whole-body energy balance, and the metabolism of glucose and fats (Viollet *et al.* 2012). Metformin also increases glucagon-like peptide-1 levels (Mannucci *et al.* 2001). Here, we found that shRNA silencing of DPP-4 reduced cell survival in metformin (Figure S6), suggesting that DPP4 may be an additional target of metformin.

Recent interest in the use of metformin as a cancer chemotherapeutic was sparked by a meta-analysis of patients with diabetes that demonstrated a statistically robust decrease of several diverse cancers in those patients taking metformin (Noto *et al.* 2012). The mechanism for the potential anticancer effects of metformin is a subject of great interest (Bost *et al.* 2012). Several investigators have hypothesized that, because insulin acts as a mitogen, metformin may not only correct hyperinsulinemia but may also decrease the growth of rapidly dividing cells. In contrast, observations with thiazolidinedione, a different insulin sensitizer, showed no such decrease in cancer incidence (Azoulay *et al.* 2012). Accordingly, further investigations into the mechanisms of action of metformin are warranted to define whether, and how, it affects cancer progression.

Our metformin screen produced additional hits for gene products implicated in apoptosis (CHEK1, CHFR, BCL2L1, and HUWE1) which, taken together, suggests that the consequence of disrupting signaling is often the induction of apoptosis. This suggests that in addition to detecting growth inhibition by perturbing new potential drug targets, the data can provide insight regarding general pathways required for viability. These data are summarized in Table 2A.

**Table 2:** shRNA interaction scores

A.	Drug	Gene	UniGene ID	shRNA Interaction Score		
				1	2	3
	Metformin	BCL2L1	Hs.516966	0.077	−0.088	−0.106
		CHEK1	Hs.24529	−0.137	−0.085	−0.05
		CHFR	Hs.656770	−0.046	−0.06	−0.118
		DPP4	Hs.368912	−0.048	−0.118	−0.086

B.	Drug	Gene	UniGene ID	shRNA Interaction Score		
				1	2	3
	Gossypol	BRAF	Hs.550061	−0.254	−0.076	−0.031
		BRCA1	Hs.194143	−0.188	0.145	−0.076
		BRCA2	Hs.34012	−0.174	−0.147	−0.034
		CHAT	Hs.302002	−0.249	0.062	−0.111
		CHEK1	Hs.24529	−0.174	−0.108	0.031
		CHFR	Hs.656770	−0.239	−0.08	0.014
		CRTAM	Hs.159523	−0.334	−0.203	0.07
		DBI	Hs.78888	−0.214	−0.204	0.119
		ERBB2	Hs.446352	−0.208	−0.031	−0.031
		HDAC2	Hs.3352	−0.209	−0.189	−0.027
		HSPA8	Hs.702021	−0.26	−0.125	0.004
		HUWE1	Hs.136905	−0.343	−0.301	−0.155
		KCND3	Hs.666367	−0.185	−0.123	−0.022
		MCL1	Hs.632486	−0.283	0.01	−0.02
		PTK2	Hs.395482	−0.178	−0.111	−0.039
		WRN	Hs.632050	−0.235	−0.15	−0.066

shRNA, short hairpin RNA.

### Gossypol and its potential mechanism of action in inhibiting cell growth

Gossypol is a natural polyphenolic compound derived from seeds, foliage, and roots of cotton plants and was first described as an endocrine disruptor (Xian *et al.* 1987). More recently, several studies have demonstrated the potential antineoplastic properties of this compound. Although its exact mechanism of action is not known, gossypol displays proapoptotic properties by inhibiting the antiapoptotic protein, MCL1 (myeloid cell leukemia sequence 1, Bcl2-related) in cancer cells (Goldsmith and Hogarty 2005; Lestini *et al.* 2009; Goldsmith *et al.* 2010). Normally, MCL1 is regulated via proteasomal degradation by the E3 ubiquitin-protein ligase HUWE1 (HECT, UBA, and WWE domain containing 1) (Hogarty 2010; Mandel *et al.* 2012). HUWE1 has been shown to target several proteins, including the antiapoptotic protein MCL1 and HDAC2 a protein involved in DNA damage response (Zhang *et al.* 2011). Moreover, elimination of HDAC2 and MCL1 by HUWE1 activity results in arrest of cell cycle progression in G1-to-S and forces cancer cells to undergo apoptosis

**Figure 4:**
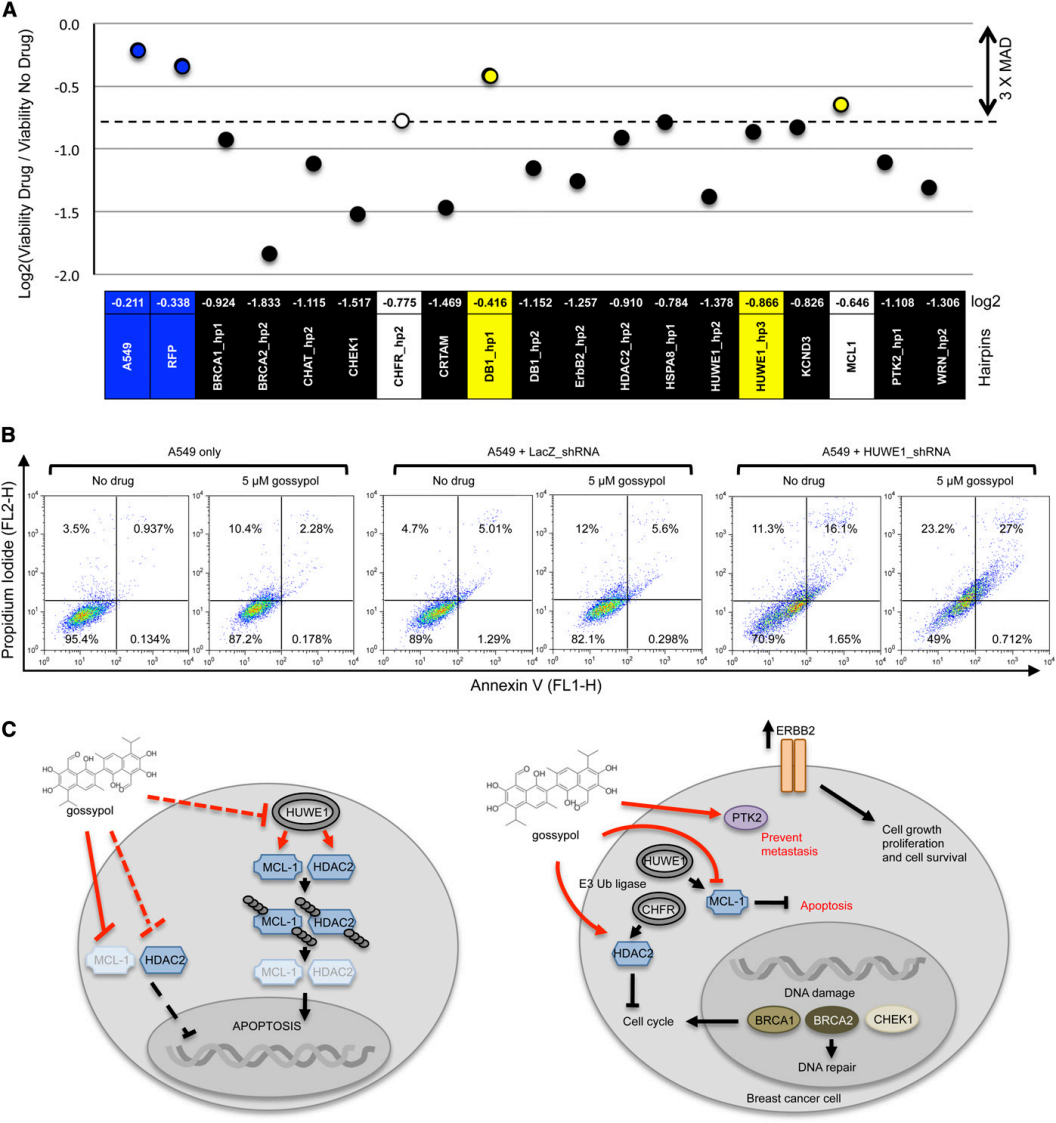
Gossypol mechanism of action. (A) Growth inhibition resulting from the silencing of candidate genes in the presence of gossypol in the A549 cell line. Regardless of the drug treatment, plate sample median was calculated and set to 100%. Then, viability for each individual hairpin was calculated relative to the sample median. Finally, the log2 value was calculated for the ratio of the viability with drug over that without drug for the same shRNA. Graphic representation (upper panel) was based on individual hairpin log2 ratio values (lower panel). Hairpins with greater or equal to three median absolute deviations (shRNA below the dashed line, in black) values from the sample median were considered as validated hits. Cells without hairpin or with a hairpin directed against RFP are used as negative controls. Hairpins that display toxicity in absence of any drug treatment are in yellow. Cell viability is assessed 3 days after drug treatment using SRB test. Individual shRNAs for selected genes are indicated (1 to 3 hairpins per gene). Analysis was performed on data collected from 3 biological replicates. (B) Detection of cell viability and cytotoxicity by flow cytometry. During gossypol treatment, HUWE1 silencing decreases A549 viability compared to cells without shRNA and with LacZ shRNA controls. After 3 d of gossypol treatment, cells were trypsinized and stained with FITC-conjugated annexin V (FL1 detection, 488 nm/515−545 nm, X-axis) and propidium iodide (FL2 detection, 488 nm/564−606 nm, Y-axis). Dot plots show the percentage of dead (top quadrants; FL1−/FL2+ and FL1+/FL2+), apoptotic (bottom right quadrant; FL1+/FL2−) and living A549 cells (bottom left quadrant; FL1−/FL2−) in the presence or absence of 5 μM gossypol. One representative example is shown where the viability of A549 partially depleted for HUWE1 (left panel) is compared with both A549 without hairpin (right panel) and A549 with a hairpin against lacZ (middle panel) as negative controls. (C) *Model for gossypol mode of action*. Our gossypol screen uncovered several potential cancer-related targets that fall into three broad categories: (1) E3 ubiquitin protein ligases, HUWE1 and CHFR; (2) breast cancer signaling proteins, ErbB2 and PTK2; and (3) cell-cycle−regulating proteins BRCA1, BRCA2, and CHEK1. This model is based on the published literature for each of the nine represented proteins.

The results of our gossypol screen are summarized in Table 2B. We focused on the top 15 potential target genes of gossypol based on the strength of their drop out (their interaction value) and visual inspection of the data (linear model fits). For these 15 potential targets, we generated individual A549 cell lines each containing one integrated shRNA and assayed their viability by SRB test (Figure 4A) and flow cytometry (Figure 4B and Figure S7). After 3 d of exposure to 5 μM of gossypol, the parent A549 cells and control A549 cells with an integrated RFP hairpin showed 81% and 77% survival, respectively, compared with cells cultured without drug (Figure S8), consistent with our initial dosing experiments. In addition, only 2 hairpins strongly reduced the cell viability in their own (Figure S8, indicated by arrows), one of which (HUWE1) was already found toxic in our screen (Figure S4). The shRNA validation was based on the median absolute deviation statistical method (Chung *et al.* 2008). Hairpins with values greater or equal to three times the median absolute deviation from the sample median were considered as validated (Figure 4A, dashed line). A total of 13 of 15 genes tested individually manifested a dramatic increase in gossypol sensitivity. These gossypol-induced synthetic lethal interactions were further confirmed by flow cytometry. Characteristic examples of drug-induced, hairpin-dependent hypersensitivity are shown in Figure 4B. As illustrated by the number of cells present on both top quadrants (composed of dead/dying cells), A549 cells cultured in the absence or presence of gossypol show an increase in sensitivity (4.4–12.7%, respectively). A similar increase in sensitivity is obtained for a control hairpin, LacZ (9.7–17.6%, respectively). In contrast, for cells carrying HUWE1 shRNAs, the fraction of dead cells increased dramatically (27.4–50.2%), suggesting that HUWE1 is required for gossypol resistance. The combined validation of 13 of 15 genes by both flow cytometry and SRB assay illustrate the low frequency of false-positive results using our stringent selection criteria. It also validates our approach to study compounds of unknown target or mode of action.

The exact roles of each of these genes in the manifestation of synthetic lethality with gossypol are not yet clear. However, on the basis of the characterization of several of these genes in the literature, we can propose potential mechanisms.

Here, we found that knock-down of HDAC2 showed a decrease in survival in response to gossypol (Figure 4A, Figure S7, and Figure S9), consistent with previous reports (Zhang *et al.* 2011). Our results also suggest that the E3 ubiquitin-protein ligase, HUWE1 is a potential target of gossypol. HUWE1 knock-down (3/3 hairpins) induces a clear loss of viability (Figure 4, A and B, Figure S7, and Figure S9), a finding in contrast to the published reports that HUWE1 depletion shows a reduced degradation of MCL1, less apoptosis and an increase in cell survival (Mandel *et al.* 2012).

Our results, combined with previous knowledge of the biological functions of the genes identified in that screen, suggest that gossypol exerts its antiproliferative effect in potentially three distinct ways: (1) by stabilization of the MCL1-HUWE1 complex; (2) by inducing apoptosis through inhibition of the anti-apoptotic protein MCL1 via HUWE1; and (3) by arresting cancer cells in G1 phase presumably via HDAC2 inhibition (Figure 4C).

In this study, we report the development and application of a sensitive, cost-effective assay to facilitate drug mode of action studies. To demonstrate the utility of this method, we screened shRNAs and drugs based on known drug−target interactions. From 50 selected drugs with diverse activities, 28 have targets that are reported in the literature. We identified 40% of known targets, several potential novel targets and related drug pathways The data for all screens is available (Table S5 and Table S6) as well as a detailed, step-by-step protocol in the Appendix. Focusing our shRNA library on protein targets for which drugs already exist has the potential for uncovering new therapeutic applications of such compounds. Translating this experimental method and data analysis routine to other cell lines and particular pathways will allow such screens to be performed using the cell culture expertise that already exists in many laboratories.

## Supplementary Material

Supporting Information

## Appendix

### Step-by-step protocol for a minipool drug target screen

To provide flexibility in term of experimental design, the minipool protocol is divided into four modules: (1) the shRNA library, (2) cell culturing, (3) test compounds, and (4) barcode assessment. This modular design allows one to adapt and combine these modules to best fit the biological question.

### A minipool (100−5000) shRNA library is selected

In our study we focused on known human therapeutic drug targets available from the TRC lentiviral libraries (http://www.broadinstitute.org/rnai/trc). A variety of filters can be applied to define the size of the library, *e.g.*, selection of hairpins with known biological activity or with known levels of knock-down (quantitative PCR), and elimination of shRNAs found empirically to be “promiscuous positives” in multiple screens.

### A cell line is selected

We chose A549 because of its ease of culture (ATCC CCL-185) and its previous use in genome-scale screens.

### A set of drugs/compounds is selected

We used the Biomol/ENZO FDA drug library as a starting point to select 50 compounds for screening.

### Barcode analysis

We use a “Barseq” approach to quantify the hairpins in the screening population that using short-read Next-Generation Sequencing.

#### Minipool drug screens:

1. Drug screening concentrations (IC_25_) are determined by generating dose−response curves for each chemical compound. We determined a screening concentration for each drug tested by (1) generating dose−response curves from data obtained with a rapid viability test in a 96-well plate format, and (2) by extrapolating the results obtained to test a narrow range of drugs in six-well plates, the format used for the screens.
  We tested a range of drug concentrations in 96-well plates as follows:
  - A549 cells were seeded in 96-well plates at 2200 cells/well in 200 μL of media
  - Drugs were serially diluted first in DMSO then in DMEM, with the final DMSO concentration not exceeding 0.5%.
  - Drugs were added to the cells as follows: one drug per lane, the first well containing vehicle control (considered the 100% growth control) and the 11 remaining wells with a serial dilution of the drug.
  - After 72 hr of incubation, cell viability was measured by SRB viability assay.
  - A549s were fixed by addition of trichloroacetic acid from a 10% stock in water to 3.3% w/v final concentration, incubated at 4° for 30 min, then washed gently five times with running tap water.
  - Fixed precipitates were air dried and 50 μL of 0.4% w/v of SRB dissolved in 1% acetic acid was added to each well and incubated at room temperature for 30 min on a rocking platform.
  - Unbound SRB was removed by four washes with 1% acetic acid and then the plates were air dried. The bound dye was solubilized by adding 150 μL of 10 mM Tris base (pH 10.5). After 10 min solubilized dye concentration was determined with a absorbance reader set at a wavelength of 510 nm (*e.g.*, BioTek Synergy 2).
  - Background signal subtraction was based on wells incubated in the absence of cells. The percentage growth inhibition was calculated by dividing the absorbance obtained from the cells treated with drug by the absorbance of the cells cultured in presence of vehicle alone (100% growth control).
  - Dose−response curves are generated to determine each drug IC_25_.
  Based on the 96-well growth data, we performed an identical experiment with a narrow range of drug in six-well plates, the format used for the screens. Performing the dosing in the same vessel as the minipool experiment provides a more robust IC_25_ for the screens.
2. Lentiviral particles are produced to create frozen “screen-sized” stocks of infected cells. Lentiviral particles harboring the set of shRNAs were produced according to the modified protocols provided by TRC (http://www.broadinstitute.org/rnai/public/resources/protocols) as described below:
  - Seed 10 mL of 293T packaging cells at 350,000 cells/mL (total 3.5 × 10^6^ cells) in low-antibiotic growth media (DMEM + 10%FBS + 0.1X Pen/Strep) in 10-cm tissue culture dishes, in triplicate.
  - Incubate at 37°, 5% CO_2_ until cells reach ~70% confluence (~24 hr).
  - Transfect packaging cells with 7.2 μg of packaging plasmid (psPAX2), 800 ng of envelope plasmid (VSV-G/pMD2.G), 8 μg of pool hairpin plasmid mix (pLKO.1) using 49 μL of FuGene 6 transfection reagent (Roche; cat. no. 11-814-443-001).
  - Incubate transfected cells for 18 hr (37°, 5% CO_2_).
  - Change media to remove transfection reagent and replace with 10 mL of high-bovine serum albumin growth media for viral harvests (500 mL DMEM + 10% FBS + P/S with 5.5 g of bovine serum albumin).
  - Incubate cells for 48 hr (37°, 5% CO_2_)
  - Harvest media containing lentivirus, transfer media to a polypropylene storage tube.
  - Centrifuge the media containing virus at 150 × g for 5 min to pellet any packaging cells that were collected during harvesting.
  - Transfer the supernatant to a sterile polypropylene storage tube.
  - Aliquot virus (to reduce the number of freeze/thaw cycles) and store at −80°
  A549 cells are infected with the lentiviral shRNA minipool at a multiplicity of infection of 0.3−0.4 (this low multiplicity of infection minimizes the number of multiple shRNA integrations) as follows:
  - 1.5 × 10^7^ cells were seeded in 40 mL of DMEM + FBS + P/S +8 μg/mL of polybrene in duplicate, virus was immediately added to the two T175 flasks.
  - 24 hr after infection, the medium with virus was replaced by media containing 2 μg/mL puromycin to kill uninfected cells.
  - After 2 d of selection, puromycin-resistant A549 cells were amplified for two additional days, resulting in a total of 4 × 10^7^ cells.
  - The transduced A549 cells were split into aliquots of 1.5 × 106 cells each and frozen for future screens. Storage medium: 50% FBS + 40% complete medium + 10% DMSO.
  Hairpin representation is assessed and toxic hairpins are identified. We tested the hairpin representations before and after thawing as well as among the different frozen aliquots as detailed below:
  - We compared two sets of infected cells. The first set contains cells collected immediately after the original infection (the “mother” population). The second set represents cells from two stock vials used in all our screens, obtained after infection, puromycin selection, amplification and one freeze-thaw cycle (the “daughter” populations 1 and 2).
  - For each sample, genomic DNA was extracted from 1 × 10^6^ cells, the shRNAs were amplified via PCR and sequenced using an Illumina HiSequation 2000.
  - A high correlation (r = 0.981) in the sequence count number for each hairpin was found between daughter populations, and a very high correlation (r = 0.947 and r = 0.932) was also found between the mother and both daughter populations.
  - Only 25 shRNAs (of 1098) were missing from our collection and most of these were lost immediately after the initial infection step.
  - Freezing aliquots following the initial infection provides a uniform source of cells for screening.
3. Screens are performed in triplicate and cells are collected at several time points throughout the experiment. We grew A549s in six-well plates so that one experiment could fit in one well, reducing cost, simplifying manipulations, and allowing us to test several drugs in parallel.
  - A549s are seeded in 2 mL of DMEM + 10% FBS + P/S + drug.
  - Cells were collected every 3 d and maintained in culture in drug over 21 d.
  - Cells are split every third day with 1.65 × 10^5^ cells reseeded per well.
4. Samples are processed for sequencing, and primary hits are selected and validated. Genomic DNA is extracted in a 96-well format using a QiaExtractor DNA robot (QIAGEN) according to the manufacturer’s instructions. Resulting samples were normalized to 1 μg and samples stored at −80°.
  shRNAs are amplified via PCR from the genomic DNA obtained from the minipool screens as follows:
  - To amplify shRNAs from genomic DNA, reactions were set up in 0.2-mL strip tubes containing 100 μL of master mix with 500 ng of genomic template DNA and 600 nM of a custom reverse Illumina adapter-barcode primer (with each barcode indicating the sample’s specific drug treatment, replicate # and time point).
  - The amplification master mixture contained 2X PCR Amplification Buffer, 2X Enhancer Buffer, 300 μM dNTPs mix, 1mM MgSO_4_, 450nM of reverse Illumina adapter primer, and 7.5 units of Platinum Pfx DNA polymerase (Invitrogen; cat. no. 11708-039).
  - The amplification reaction was performed by denaturating once at 94° for 5 min, followed by (94° for 15 sec, 55° for 15 sec, 68° for 20 sec) × 28 cycles and a final extension step at 68° for 5min, then cooling to 4°.
  *Note: Hairpins are PCR amplified using desalted primers including Illumina adaptors and a barcode specific for each condition*.
  Forward primer sequence: 5′ –CAAGCAGAAGACGGCATACGAGATNNNNNNNNTGTGGATGAATACTGCCATTTGTCTCGAGGTC
  Reverse primer sequence: 5′-AATGATACGGCGACCACCGAGATCAATGGACTATCATATGCTTACCGTAACTTGAA
  *Italics: The variable sequence NNNNNNNN represents the 8-mer indexing tag used in multiplexing/index readout*.
  The shRNA PCR product is prepared for next generation sequencing on the Illumina platform as follows:
  - The PCR product size was verified on a 2% agarose gel to confirm that there was a preponderance of a ~234-bp PCR product (178 bp for hairpin + 48 bp for Illumina adaptor + and 8 nt for barcode).
  - PCR products were pooled (30 μL for each sample) and purified using a QIAGEN PCR purification kit (cat. no. 28106).
  - Purified pooled samples were separated by agarose gel electrophoresis, and the 234-bp product was excised.
  - MinElute PCR purification (cat. no. 28004) was used to extract DNA from the gel slice prior to sequencing on an Illumina HiSeq2000.
  Next-generation sequencing: we used the Illumina HiSeq2000 sequencing platform as described below:
  - Normalized DNA template was quantified by real-time PCR, diluted to 8 pM, and clusters were generated on a single-read flow cell.
  - Samples were divided into two batches, each consisting of 25 drug treatments plus vehicle control, at eight time points each, in triplicate.
  - An 8-mer index sequence unique to each sample was incorporated along with the Illumina adapters before clustering onto the flowcell to allow multiplexing of 96 samples within each flowcell lane.
  - The hairpin sequence was decoded using the sequence primer 5′- gatttcttggctttatatatcttgtggaaaggacgaaacaccgg, and collecting 20 cycles of sequencing.
  - A second read of eight cycles of sequencing was used to decode the sample’s barcode/index using the primer 5′-gaattctcgacctcgagacaaatggcagtattcatccaca.
  Sequencing data are analyzed as follows:
  - Hairpins were matched to sequence reads, allowing up to two mismatches per alignment, in our experiments, the median percentage of reads assigned to a hairpin across all samples was 97%.
  - The sequence reads for individual screens were demultiplexed by virtue of their unique index sequences.
  - At the time of the experiment, 96 samples could be processed at once in a single flowcell lane producing an average count of ~656 reads for each of the 1098 hairpins in the pool.
  Primary hits are selected based on both their interaction values and individual linear plots:
  - To assess the combined impact of shRNA knock-down and drug treatment on cell growth, a linear model was fit to the observed triplicate data for each hairpin.
  - The difference between DMSO controls and drug treatment is designated as the “interaction value,” a term that reflects the abundance of shRNAs in the population during the course of drug treatment.
  - For example, any shRNA that renders a cell hypersensitive is characterized by a negative interaction value. Hairpins were ranked according to the interaction values as a measure of the effect of drug treatment while controlling for the effect of the hairpin treatment.
  - Hairpins with interaction values that are 2 SD from the screen mean (those synthetic lethal or those rescuing the drug toxicity) were considered as primary hits.

### Visual inspection of individual linear plots to reduce the number of false-positive results

Candidate genes are then individually validated with the use of identical shRNAs and additional hairpins. We retested 15 potential synthetic lethal genes found in the minipool screen performed with gossypol. The viability of cell line containing individual shRNAs was then quantified by both SRB assay and flow cytometry, and we found 13 out of 15 genes validated.

The use of alternative hairpins or RNAi method (siRNA, esiRNA) for candidate gene validation is recommended to minimize off-target effects that could interfere with the interpretation of the results. Cell viability can be determined by several methods (vital dyes, cellular metabolism, *e.g.*, ATP or MTT, apoptosis assay).
