# Supplementary material for: Miniature Short Hairpin RNA Screens to Characterize Antiproliferative Drugs
Source: G3 (Bethesda). 2013 Aug 1;3(8):1375–87. doi: 10.1534/g3.113.006437 (PMC3737177; doi:10.1534/g3.113.006437)
Supplement: Supporting Information [file supp_3_8_1375__index.html]

Miniature Short Hairpin RNA Screens to Characterize Antiproliferative Drugs — Supporting Information 

# Miniature Short Hairpin RNA Screens to Characterize Antiproliferative Drugs

## Supporting Information for Kittanakom *et al.*, 2013

**Files in this Data Supplement:**

- Supporting Information - Figures S1-S9, Tables S1-S7, and References (PDF, 9 MB)
- Figure S1 - List of human gene targets in the minipool grouped by gene ontology (PDF, 5 MB)
- Figure S2 - Hairpin representation in the A549 cells used for the synthetic lethality drug screen (PDF, 665 KB)
- Figure S3 - Hairpin representations in the A549 cells used for the synthetic lethality drug screen (PDF, 1 MB)
- Figure S4 - List of toxic hairpins (PDF, 219 KB)
- Figure S5 - Correlation plots between triplicate (A, B, and C) of metformin (A.) and gossypol (B.) screen. (PDF, 963 KB)
- Figure S6 - Linear plots of an individual gene that was knocking down by shRNA in A549 cells cultured in the presence of metformin are BCL2L1, CHEK1, CHFR and DPP4 respectively. (PDF, 228 KB)
- Figure S7 - Percentage of dead/dying (FL1-/FL2+ and FL1+/FL2+), apoptotic (FL1+/FL2-) and living cells (FL1-/FL2-) in the presence of gossypol in the A549 cell lines silenced for the indicated candidate genes. (PDF, 146 KB)
- Figure S8 - Effect of the hairpins targeting the gossypol potential hit genes on A549 viability (PDF, 266 KB)
- Figure S9 - Potential gossypol targets are shown in sixteen linear plots (PDF, 348 KB)
- Table S1 - Compounds used in the screens and their published GI50s (drug concentrations that causes 50% cell growth inhibition). (PDF, 145 KB)
- Table S3 - Linear plots of the top hairpins for the 50 tested drugs (PDF, 6 MB)
- Table S4 - List of potential targets for each of the 28 drugs with reported targets (PDF, 112 KB)
- References - PDF, 81 KB
- Table S2 - List of the calculated hairpin interaction values for the 50 tested drugs (.xlsx, 2 MB)
- Table S5 - Normalized count number for individual hairpin in the A549s cultured in presence of indicated drugs (.xlsx, 10 MB)
- Table S6 - Normalized count number for individual hairpin in the A549s cultured in presence of indicated drugs (.xlsx, 11 MB)
- Table S7 - Percentage of remaining gene expression assessed by qPCR in A549, MCF7 or HEK293T/17 for each hairpins of the minipool collection (source CCBR-OICR Lentiviral Technology Cancer). (xlsx, 69 KB)
