# Supplementary material for: Miniature Short Hairpin RNA Screens to Characterize Antiproliferative Drugs
Source: G3 (Bethesda). 2013 Aug 1;3(8):1375–87. doi: 10.1534/g3.113.006437 (PMC3737177; doi:10.1534/g3.113.006437)
Supplement: Supporting Information [file supp_g3.113.006437_006437SI.pdf]

G3 Genes | Genomes | Genetics

Miniature shRNA screens to characterize antiproliferative drugs

Saranya Kittanakom[1,2,3,*], Anthony Arnoldo[1,2,3,*], Kevin R. Brown[2,3], Iain Wallace[1,2,3,#], Tada Kunavisarut[4], Dax Torti[5], Lawrence E. Heisler[1,2,3,5], Anuradha Surendra[5], Jason Moffat[1,3], Guri Giaever[1,3,6,7], and Corey Nislow[1,2,3,5,7**]

[1]Department of Molecular Genetics, University of Toronto, Canada
[2]Banting and Best Department of Medical Research, University of Toronto, Canada
[3]Terrence Donnelly Centre for Cellular and Biomolecular Research, University of Toronto, 160 College Street, Toronto, Ontario M5S 3E1, Canada
[4]Division of Endocrinology and Metabolism, Department of Medicine, Faculty of Medicine, Siriraj Hospital, Mahidol University, Thailand
[5]Donnelly Sequencing Center, University of Toronto, 160 College Street, Toronto, Ontario M5S 3E1, Canada
[6]Department of Pharmaceutical Sciences, University of Toronto, 144 College Street, Toronto, Ontario M5S 3M2, Canada
[7]Department of Pharmaceutical Sciences, University of British Columbia, Vancouver, BC, V6T1Z3
[#]Current address Novartis, 250 Massachusetts Ave., Cambridge 02139, USA

* These authors contributed equally to this work
** Corresponding author

DOI: 10.1534/g3.113.006437

Category	Symbol	GeneID	Approved Name
Carbohydrate metabolism	ACE	1636	angiotensin I converting enzyme (peptidyl-dipeptidase A) 1
	AKR1B1	231	aldo-keto reductase family 1, member B1 (aldose reductase)
	AKT1	207	v-akt murine thymoma viral oncogene homolog 1
	B3GAT1	27087	beta-1,3-glucuronyltransferase 1 (glucuronosyltransferase P)
	DCXR	51181	dicarbonyl/L-xylulose reductase
	INSR	3643	insulin receptor
	MGAM	8972	maltase-glucoamylase (alpha-glucosidase)
	MMP13	4322	matrix metallopeptidase 13 (collagenase 3)
	MMP3	4314	matrix metallopeptidase 3 (stromelysin 1, progelatinase)
	MMP7	4316	matrix metallopeptidase 7 (matrilysin, uterine)
Cell cycle	CCND2	894	cyclin D2
	KNTC1	9735	kinetochore associated 1
	MAD1L1	8379	MAD1 mitotic arrest deficient-like 1 (yeast)
	BRIP1	83990	BRCA1 interacting protein C-terminal helicase 1
	CDKN1A	1026	cyclin-dependent kinase inhibitor 1A (p21, Cip1)
Cell organization and biogenesis	ABL1	25	c-abl oncogene 1, receptor tyrosine kinase
	BBC3	27113	BCL2 binding component 3
	BCL2	596	B-cell CLL/lymphoma 2
	BCL2L1	598	BCL2-like 1
	BCL2L7	578	BCL2-antagonist/killer 1
	BLM	641	Bloom syndrome, RecQ helicase-like
	BRCA1	672	breast cancer 1, early onset
	BRCA2	675	breast cancer 2, early onset
	BUB1B	701	budding uninhibited by benzimidazoles 1 homolog beta (yeast)
	CDKN2A	1029	cyclin-dependent kinase inhibitor 2A (melanoma, p16, inhibits CDK4)
	CFTR	1080	cystic fibrosis transmembrane conductance regulator (ATP-binding cas
	KRAS	3845	v-Ki-ras2 Kirsten rat sarcoma viral oncogene homolog
	MARK2	2011	MAP/microtubule affinity-regulating kinase 2
	MOAP1	64112	modulator of apoptosis 1
	NEK2	4751	NIMA (never in mitosis gene a)-related kinase 2
	PIM2	11040	pim-2 oncogene
	PTK2	5747	PTK2 protein tyrosine kinase 2
	SEC23IP	11196	SEC23 interacting protein
	STK36	27148	serine/threonine kinase 36
	TNK2	10188	tyrosine kinase, non-receptor, 2
	TOP2A	7153	topoisomerase (DNA) II alpha 170kDa
	TP53	7157	tumor protein p53
	TP63	8626	tumor protein p63
	ZW10	9183	ZW10, kinetochore associated, homolog (Drosophila)
	ZWINT	11130	ZW10 interactor
	WRN	7486	Werner syndrome ATP-dependent helicase
	PMAIP1	5366	phorbol-12-myristate-13-acetate-induced protein 1
	PML	5371	promyelocytic leukemia
	TTK	7272	TTK protein kinase
DNA damage response	ATM	472	ataxia telangiectasia mutated
	CSNK1E	1454	casein kinase 1, epsilon
	FANCA	2175	Fanconi anemia, complementation group A
	MLL	4297	myeloid/lymphoid or mixed-lineage leukemia (trithorax homolog, Droso
	MTOR	2475	mechanistic target of rapamycin (serine/threonine kinase)
	NSMCE1	197370	non-SMC element 1 homolog (S. cerevisiae)
	SMG1	23049	SMG1 homolog, phosphatidylinositol 3-kinase-related kinase (C. elega
	TP73	7161	tumor protein p73
	TYMS	7298	thymidylate synthetase
	XPC	7508	xeroderma pigmentosum, complementation group C
	CHEK1	1111	CHK1 checkpoint homolog (S. pombe)
	CHEK2	11200	CHK2 checkpoint homolog (S. pombe)
	RAD17	5884	RAD17 homolog (S. pombe)
	APC	324	adenomatous polyposis coli
	POLH	5429	POLH polymerase (DNA directed), eta

DNA metabolism	DAPK3	1613	death-associated protein kinase 3
	ERCC2	2068	excision repair cross-complementing rodent repair deficiency, complementation group
	ERCC3	2071	excision repair cross-complementing rodent repair deficiency, complementation group
	HDAC1	3065	histone deacetylase 1
	HDAC10	83933	histone deacetylase 10
	HDAC11	79885	histone deacetylase 11
	HDAC2	3066	histone deacetylase 2
	HDAC3	8841	histone deacetylase 3
	HDAC4	9759	histone deacetylase 4
	HDAC5	10014	histone deacetylase 5
	HDAC6	10013	histone deacetylase 6
	HDAC8	55869	histone deacetylase 8
	HDAC9	9734	histone deacetylase 9
	MYST4	23522	MYST histone acetyltransferase (monocytic leukemia) 4
	NR3C1	2908	nuclear receptor subfamily 3, group C, member 1 (glucocorticoid receptor)
	RB1	5925	retinoblastoma 1
	RECQL4	9401	RecQ protein-like 4
	TOP1	7150	topoisomerase (DNA) I
	TOP2B	7155	topoisomerase (DNA) II beta 180kDa
	HDAC7	51564	histone deacetylase 7
	CAT	847	catalase
	TUBA4A	7277	tubulin, alpha 4a
	TUBB	203068	tubulin, beta
	UGT2B11	10720	UGT2B11 UDP glucuronosyltransferase 2 family, polypeptide B11
	ACHE	43	acetylcholinesterase
	TCF3	6929	transcription factor 3 (E2A immunoglobulin enhancer binding factors E12/E47)
Metabolism	ADK	132	adenosine kinase
	AKR1A1	10327	aldo-keto reductase family 1, member A1 (aldehyde reductase)
	ALDH1A1	216	aldehyde dehydrogenase 1 family, member A1
	ALOX12	239	arachidonate 12-lipoxygenase
	ALOX15	246	arachidonate 15-lipoxygenase
	ALOX5	240	arachidonate 5-lipoxygenase
	BHMT	635	betaine--homocysteine S-methyltransferase
	CA1	759	carbonic anhydrase I
	CHDH	55349	choline dehydrogenase
	COMT	1312	catechol-O-methyltransferase
	DHFR	1719	dihydrofolate reductase
	DPYS	1807	dihydropyrimidinase
	FDFT1	2222	farnesyl-diphosphate farnesyltransferase 1
	FDPS	2224	farnesyl diphosphate synthase
	FKBP1A	2280	FK506 binding protein 1A, 12kDa
	FKBP2	2286	FK506 binding protein 2, 13kDa
	FKBP3	2287	FK506 binding protein 3, 25kDa
	FKBP5	2289	FK506 binding protein 5
	FPGS	2356	folylpolyglutamate synthase
	GGH	8836	gamma-glutamyl hydrolase (conjugase, folylpolygammaglutamyl hydrolase)
	GLUL	2752	glutamate-ammonia ligase
	HMGCR	3156	3-hydroxy-3-methylglutaryl-CoA reductase
	LACTB	114294	lactamase, beta
	LIPF	8513	lipase, gastric
	LSS	4047	lanosterol synthase (2,3-oxidosqualene-lanosterol cyclase)
	MAOB	4129	monoamine oxidase B
	MMAB	326625	methylmalonic aciduria (cobalamin deficiency) cblB type
	MTHFS	10588	5,10-methenyltetrahydrofolate synthetase (5-formyltetrahydrofolate cyclo-ligase)
	NQO1	1728	NAD(P)H dehydrogenase, quinone 1
	PNPLA6	10908	patatin-like phospholipase domain containing 6
	PON1	5444	paraoxonase 1
	PPAP2B	8613	phosphatidic acid phosphatase type 2B
	PPIA	5478	peptidylprolyl isomerase A (cyclophilin A)
	S100B	6285	S100 calcium binding protein B

Metabolism	STS	412	steroid sulfatase (microsomal), isozyme S
	TH	7054	tyrosine hydroxylase
	TPMT	7172	thiopurine S-methyltransferase
	UGDH	7358	UDP-glucose 6-dehydrogenase
	UMPS	7372	uridine monophosphate synthetase
	XDH	7498	xanthine dehydrogenase
	ARSE	415	arylsulfatase E (chondrodysplasia punctata 1)
	GSTA1	2938	glutathione S-transferase alpha 1
	HPRT1	3251	hypoxanthine phosphoribosyltransferase 1
	NMNAT1	64802	nicotinamide nucleotide adenylyltransferase 1
	PTGS2	5743	prostaglandin-endoperoxide synthase 2 (prostaglandin G/H synthase a
	SQLE	6713	squalene epoxidase
	DCK	1633	deoxycytidine kinase
	TYR	7299	tyrosinase (oculocutaneous albinism IA)
	OAT	4942	ornithine aminotransferase
	ABP1	26	amiloride binding protein 1 (amine oxidase (copper-containing))
	CES2	8824	carboxylesterase 2 (intestine, liver)
	CHAT	1103	choline O-acetyltransferase
	CPA6	57094	CPA6 carboxypeptidase A6
	EBP	10682	EBP emopamil binding protein (sterol isomerase)
	FH	2271	fumarate hydratase
	FMO1	2326	flavin containing monooxygenase 1
	GRIN1	2902	GRIN1 glutamate receptor, ionotropic, N-methyl D-aspartate 1
	HDC	3067	histidine decarboxylase
	IMPDH1	3614	IMP (inosine 5'-monophosphate) dehydrogenase 1
	MME	4311	membrane metallo-endopeptidase
	NAT2	10	NAT2 N-acetyltransferase 2 (arylamine N-acetyltransferase)
	NR1H4	9971	nuclear receptor subfamily 1, group H, member 4
	PAM	5066	PAM peptidylglycine alpha-amidating monooxygenase
	REN	5972	renin
	SHBG	6462	sex hormone-binding globulin
	TPO	7173	TPO thyroid peroxidase
	PPP3CB	5532	protein phosphatase 3, catalytic subunit, beta isozyme
	PTPN1	5770	protein tyrosine phosphatase, non-receptor type 1
Protein amino acid phosphorylation	ALK	238	anaplastic lymphoma receptor tyrosine kinase
	AURKB	9212	aurora kinase B
	AURKC	6795	aurora kinase C
	CAMK1	8536	calcium/calmodulin-dependent protein kinase I
	CAMKK1	84254	calcium/calmodulin-dependent protein kinase kinase 1, alpha
	CCND1	595	cyclin D1
	CDK2	1017	cyclin-dependent kinase 2
	CDK4	1019	cyclin-dependent kinase 4
	CDK5	1020	cyclin-dependent kinase 5
	CDK6	1021	cyclin-dependent kinase 6
	DAPK2	23604	death-associated protein kinase 2
	DGUOK	1716	deoxyguanosine kinase
	F2	2147	coagulation factor II (thrombin)
	FER	2241	fer (fps/fes related) tyrosine kinase
	FGFR1	2260	fibroblast growth factor receptor 1
	FGR	2268	Gardner-Rasheed feline sarcoma viral (v-fgr) oncogene homolog
	FYN	2534	FYN oncogene related to SRC, FGR, YES
	GAK	2580	cyclin G associated kinase
	HCK	3055	hemopoietic cell kinase
	IKBKB	3551	inhibitor of kappa light polypeptide gene enhancer in B-cells, kinase be
	JAK1	3716	Janus kinase 1
	JAK2	3717	Janus kinase 2
	LYN	4067	v-yes-1 Yamaguchi sarcoma viral related oncogene homolog
	MKNK2	2872	MAP kinase interacting serine/threonine kinase 2
	MYLK2	85366	myosin light chain kinase 2
	NTRK1	4914	neurotrophic tyrosine kinase, receptor, type 1

Protein amino acid phosphorylation	PDGFRB	5159	platelet-derived growth factor receptor, beta polypeptide
	PIK3CA	5290	phosphoinositide-3-kinase, catalytic, alpha polypeptide
	PLK1	5347	polo-like kinase 1
	PLK4	10733	polo-like kinase 4
	PRKAA1	5562	protein kinase, AMP-activated, alpha 1 catalytic subunit
	RIPK2	8767	receptor-interacting serine-threonine kinase 2
	RPS6KA2	6196	ribosomal protein S6 kinase, 90kDa, polypeptide 2
	SRC	6714	v-src sarcoma (Schmidt-Ruppin A-2) viral oncogene homolog (avian)
	STK17B	9262	serine/threonine kinase 17b
	STK38L	23012	serine/threonine kinase 38 like
	YES1	7525	v-yes-1 Yamaguchi sarcoma viral oncogene homolog 1
	ERBB2	2064	Receptor tyrosine-protein kinase erbB-2
	ERBB3	2065	Receptor tyrosine-protein kinase erbB-3
	ERBB4	2066	Receptor tyrosine-protein kinase erbB-4
	IGF1R	3480	Insulin-like growth factor 1 receptor
	BRAF	673	v-raf murine sarcoma viral oncogene homolog B1
	MAP2K1	5604	mitogen-activated protein kinase kinase 1
	MAP2K4	6416	mitogen-activated protein kinase kinase 4
	MAPK8	5599	mitogen-activated protein kinase 8
	MAPK9	5601	mitogen-activated protein kinase 9
	EPHA2	1969	EPH receptor A2
	LCK	3932	lymphocyte-specific protein tyrosine kinase
	STK10	6793	serine/threonine kinase 10
	STK16	8576	serine/threonine kinase 16
	STK4	6789	serine/threonine kinase 4
	BCR	613	breakpoint cluster region
	ATR	545	ataxia telangiectasia and Rad3 related
	BMP2K	55589	BMP2 inducible kinase
	BMPR1A	657	bone morphogenetic protein receptor, type IA
	CLK1	1195	CDC-like kinase 1
	FRK	2444	fyn-related kinase
	PRKCA	5578	protein kinase C, alpha
	SLK	9748	SLK STE20-like kinase
Protein degradation	ADAM17	6868	ADAM metallopeptidase domain 17
	CAPN1	823	calpain 1, (mu/I) large subunit
	CHFR	55743	checkpoint with forkhead and ring finger domains
	CTSG	1511	cathepsin G
	DPP4	1803	dipeptidyl-peptidase 4
	LTA4H	4048	leukotriene A4 hydrolase
	MGEA5	10724	meningioma expressed antigen 5 (hyaluronidase)
	PGA4	643847	pepsinogen 4, group I (pepsinogen A)
	PLG	5340	plasminogen
	PRCP	5547	prolylcarboxypeptidase (angiotensinase C)
	PRSS1	5644	protease, serine, 1 (trypsin 1)
	PYCARD	29108	PYD and CARD domain containing
	VKORC1	79001	vitamin K epoxide reductase complex, subunit 1
	HSPCA	3320	heat shock protein 90kDa alpha (cytosolic), class A member 2
Protein transport	SLC15A1	6564	solute carrier family 15 (oligopeptide transporter), member 1
	ARHH	399	ras homolog gene family, member H
	RASD1	51655	RAS, dexamethasone-induced 1
	TSPO	706	translocator protein (18kDa)
	CD24	1E+08	Signal transducer CD24
	DICER1	23405	dicer 1, ribonuclease type III
	FIP1L1	81608	FIP1 like 1 (S. cerevisiae)

Signal transduction	ADORA1	134	adenosine A1 receptor
	ADORA2A	135	adenosine A2a receptor
	ADRA1B	147	adrenergic, alpha-1B-, receptor
	ADRB1	153	adrenergic, beta-1-, receptor
	AGTR1	185	angiotensin II receptor, type 1
	AGTR2	186	angiotensin II receptor, type 2
	AVPR1A	552	arginine vasopressin receptor 1A
	BDKRB2	624	bradykinin receptor B2
	CCND3	896	cyclin D3
	CRABP1	1381	cellular retinoic acid binding protein 1
	CREBBP	1387	CREB binding protein
	CXCR4	7852	chemokine (C-X-C motif) receptor 4
	CYSLTR1	10800	cysteinyl leukotriene receptor 1
	DRD4	1815	dopamine receptor D4
	EDNRA	1909	endothelin receptor type A
	GNRHR	2798	gonadotropin-releasing hormone receptor
	GPBAR1	151306	G protein-coupled bile acid receptor 1
	GPR109A	338442	G protein-coupled receptor 109A
	GRB2	2885	growth factor receptor-bound protein 2
	ITGA2B	3674	integrin, alpha 2b (platelet glycoprotein IIb of IIb/IIIa complex, antigen C
	LTB4R	1241	leukotriene B4 receptor
	MLNR	2862	motilin receptor
	MTNR1A	4543	melatonin receptor 1A
	NCOA2	10499	nuclear receptor coactivator 2
	NISCH	11188	nischarin
	NR1I2	8856	nuclear receptor subfamily 1, group I, member 2
	OXTR	5021	oxytocin receptor
	P2RX1	5023	purinergic receptor P2X, ligand-gated ion channel, 1
	P2RY1	5028	purinergic receptor P2Y, G-protein coupled, 1
	P2RY4	5030	pyrimidinergic receptor P2Y, G-protein coupled, 4
	PDE11A	50940	phosphodiesterase 11A
	PDE1A	5136	phosphodiesterase 1A, calmodulin-dependent
	PDE3A	5139	phosphodiesterase 3A, cGMP-inhibited
	PPARG	5468	peroxisome proliferator-activated receptor gamma
	RARA	5914	retinoic acid receptor, alpha
	RARB	5915	retinoic acid receptor, beta
	RXRA	6256	retinoid X receptor, alpha
	RYR1	6261	ryanodine receptor 1 (skeletal)
	SSTR1	6751	somatostatin receptor 1
	TACR1	6869	tachykinin receptor 1
	TBXA2R	6915	thromboxane A2 receptor
	TLR3	7098	toll-like receptor 3
	TLR7	51284	toll-like receptor 7
	MCL1	4170	myeloid cell leukemia sequence 1 (BCL2-related)
	ARHGEF12	23365	Rho guanine nucleotide exchange factor (GEF) 12
	CXCR2	3579	chemokine (C-X-C motif) receptor 2
	IL2RA	3559	interleukin 2 receptor, alpha
	IL2RB	3560	interleukin 2 receptor, beta
	IL2RG	3561	interleukin 2 receptor, gamma
	LOX	4015	lysyl oxidase
	NR1I3	9970	nuclear receptor subfamily 1, group I, member 3
	OPRL1	4987	opiate receptor-like 1
	PGR	5241	progesterone receptor
	PTAFR	5724	platelet-activating factor receptor
	PTGER1	5731	prostaglandin E receptor 1 (subtype EP1), 42kDa
	TRHR	7201	thyrotropin-releasing hormone receptor
	CXCR1	3577	chemokine (C-X-C motif) receptor 1
	GPR44	11251	G protein-coupled receptor 44
	HRH1	3269	HRH1 histamine receptor H1
	HRH2	3274	HRH2 histamine receptor H2
	CNR1	1268	cannabinoid receptor 1 (brain)
	DBI	1622	DBI diazepam binding inhibitor (GABA receptor modulator, acyl-CoA bi
	EGFR	1956	epidermal growth factor receptor
	FPR1	2357	formyl peptide receptor 1
	ITGB3	3690	integrin, beta 3 (platelet glycoprotein IIIa, antigen CD61)
	NPC1L1	29881	NPC1 (Niemann-Pick disease, type C1, gene)-like 1
	VDR	7421	vitamin D (1,25- dihydroxyvitamin D3) receptor
	INSR	3643	Insulin receptor

Stress response	GSR	2936	glutathione reductase
	HSPA4	3308	heat shock 70kDa protein 4
	HSPA8	3312	heat shock 70kDa protein 8
	PPARA	5465	peroxisome proliferator-activated receptor alpha
Transcription	ETV6	2120	ets variant 6
	HUWE1	10075	HECT, UBA and WWE domain containing 1
	PPARD	5467	peroxisome proliferator-activated receptor delta
	RXRB	6257	retinoid X receptor, beta
	WT1	7490	Wilms tumor 1
Transport	CYP2A6	1548	cytochrome P450, family 2, subfamily A, polypeptide 6
	CYP2C9	1559	cytochrome P450, family 2, subfamily C, polypeptide 9
	CYP3A4	1576	cytochrome P450, family 3, subfamily A, polypeptide 4
	SLC12A2	6558	solute carrier family 12 (sodium/potassium/chloride transporters), mem
	SLC18A1	6570	solute carrier family 18 (vesicular monoamine), member 1
	SLC19A1	6573	solute carrier family 19 (folate transporter), member 1
	SLC22A6	9356	solute carrier family 22 (organic anion transporter), member 6
	SLC29A1	2030	solute carrier family 29 (nucleoside transporters), member 1
	SLC35A2	7355	solute carrier family 35 (UDP-galactose transporter), member A2
	SLCO1A2	6579	solute carrier organic anion transporter family, member 1A2
	SLCO1B1	10599	solute carrier organic anion transporter family, member 1B1
	SLCO1B3	28234	solute carrier organic anion transporter family, member 1B3
	SLCO4A1	28231	solute carrier organic anion transporter family, member 4A1
	SLCO4C1	353189	solute carrier organic anion transporter family, member 4C1
	ABCC3	8714	ATP-binding cassette, sub-family C (CFTR/MRP), member 3
	ABCG2	9429	ATP-binding cassette, sub-family G (WHITE), member 2
	ACCN1	40	amiloride-sensitive cation channel 1, neuronal
	AKR1C1	1645	aldo-keto reductase family 1, member C1 (dihydrodiol dehydrogenase
	AMD1	262	adenosylmethionine decarboxylase 1
	CACNA1G	8913	calcium channel, voltage-dependent, T type, alpha 1G subunit
	CACNA1S	779	calcium channel, voltage-dependent, L type, alpha 1S subunit
	CACNA2D1	781	calcium channel, voltage-dependent, alpha 2/delta subunit 1
	CNGA1	1259	cyclic nucleotide gated channel alpha 1
	CPT2	1376	carnitine palmitoyltransferase 2
	FADS2	9415	fatty acid desaturase 2
	FKBP4	2288	FK506 binding protein 4, 59kDa
	GC	2638	group-specific component (vitamin D binding protein)
	KCNA5	3741	potassium voltage-gated channel, shaker-related subfamily, member 5
	KCND3	3752	potassium voltage-gated channel, Shal-related subfamily, member 3
	PCTP	58488	phosphatidylcholine transfer protein
	SCN2A	6326	sodium channel, voltage-gated, type II, alpha subunit
	SIGMAR1	10280	sigma non-opioid intracellular receptor 1
	TCN2	6948	transcobalamin II
	TRPV3	162514	transient receptor potential cation channel, subfamily V, member 3
	ABCB1	5243	ATP-binding cassette, sub-family B (MDR/TAP), member 1
	ALO17	57674	RNF213 ring finger protein 213
Unknown	BCL2L10	10017	BCL2-like 10 (apoptosis facilitator)
	BCL2L11	10018	BCL2-like 11 (apoptosis facilitator)
	BCL2L13	23786	BCL2-like 13 (apoptosis facilitator)
	BCL2L14	79370	BCL2-like 14 (apoptosis facilitator)
	BCL2L2	599	BCL2-like 2
	BCL2L4	581	BCL2-associated X protein
	BCL2L9	666	BCL2-related ovarian killer
	CDK16	5127	cyclin-dependent kinase 16
	CRTAM	56253	cytotoxic and regulatory T cell molecule
	ELANE	1991	elastase, neutrophil expressed
	EPYC	1833	epiphycan
	HIST1H4I	8294	histone cluster 1, H4i
	KSP	3832	KIF11 kinesin family member 11
	MAD2L1BP	9587	MAD2L1 binding protein
	PEBP1	5037	phosphatidylethanolamine binding protein 1
	S100A13	6284	S100 calcium binding protein A13
	SFTPC	6440	surfactant protein C
	ZWILCH	55055	Zwilch, kinetochore associated, homolog (Drosophila)
	CD44	960	CD44 antigen
	CD133	8842	Prominin-1
	OGFR	11054	opioid growth factor receptor
	ADCY10	55811	adenylate cyclase 10 (soluble)

Figure S1 List of human gene targets in the minipool grouped by gene ontology (gray box).

A.

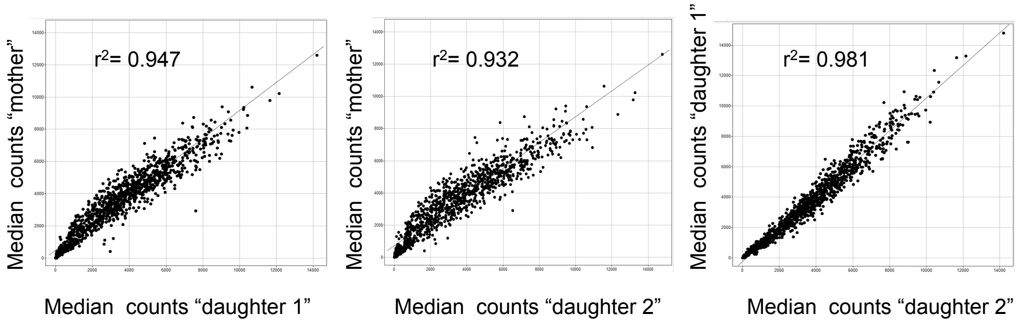

B.

	Mother	Daughter 1	Daughter 2
Hairpins missing	19.24 ± 1.70	24.5 ± 0.65	25.5 ± 1.04

Figure S2 Hairpin representation in the A549 cells used for the synthetic lethality drug screen. (A) Correlation of hairpin counts between freshly transduced A549s (“mother”) and two derived independent cell stocks (“daughter 1” and “daughter 2”). Median counts were calculated from 4 technical replicates. Raw count ranged from 0 to 17,000. (B) Hairpins present in freshly transduced A549s and two derived independent cell stocks. After normalization, hairpins with a count number below 50 were considered missing from the minipool population. Median counts were calculated from 4 technical replicates.

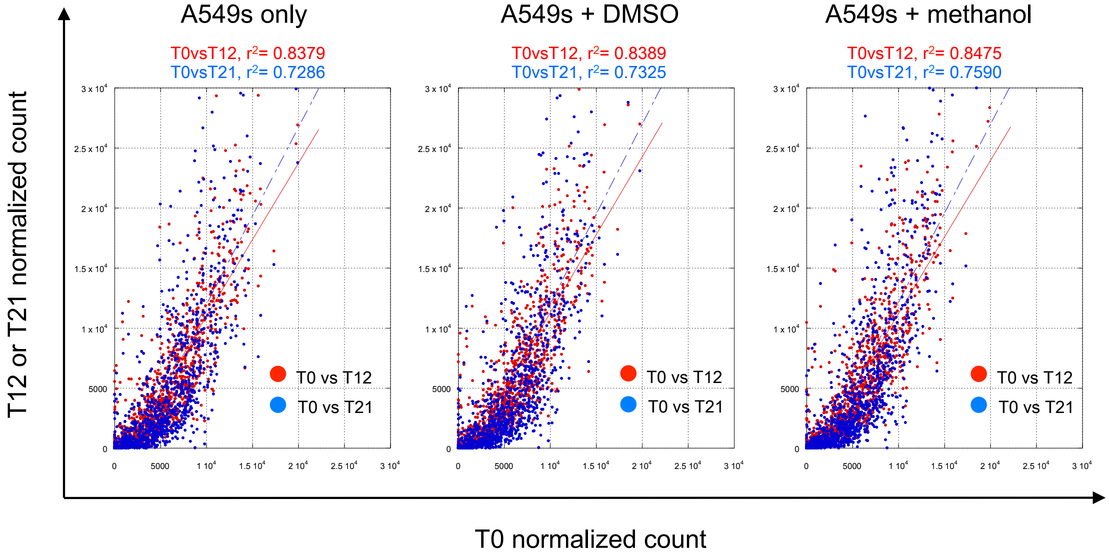

Figure S3 Hairpin representations in the A549 cells used for the synthetic lethality drug screen. Scatter plots comparing the hairpin representations at T0 vs T12 (red dots) and T0 vs. T21 (blue dots) of the A549s cultured in the absence of control vehicle (left panel), in the presence of DMSO (middle panel) or in the presence of methanol (right panel). Hairpin count was based on the results of experiments done in triplicate.

Cells only

T0 vs T12		T0 vs T21	
hpid	*gene*	*hpid*	*gene*
TRCN0000045929	FPGS	TRCN0000045929	FPGS
TRCN0000004828	HDAC3	TRCN0000056848	TLR3
TRCN0000006988	STK36	TRCN0000006988	STK36
TRCN0000117932	KNTC1	TRCN0000117932	KNTC1
TRCN0000044140	CNGA1	TRCN0000044140	CNGA1
TRCN0000195066	BRAF	TRCN0000195066	BRAF
TRCN0000000861	PRKAA1	TRCN0000000861	PRKAA1
TRCN0000121193	IGF1R	TRCN0000121193	IGF1R
TRCN0000195487	FRK	TRCN0000195487	FRK
TRCN0000053951	S100A13	TRCN0000047354	RASD1
TRCN0000002814	PPP3CB	TRCN0000002814	PPP3CB
TRCN0000001625	STK4	TRCN0000001625	STK4
TRCN0000196538	ATR	TRCN0000196538	ATR
TRCN0000078448	HDC	TRCN0000058753	IL2RB
TRCN0000009065	HRH1	TRCN0000221544	MTOR
TRCN0000222166	MYST4	TRCN0000003671	CTSG
TRCN0000074361	TUBB	TRCN0000003091	CPT2
TRCN0000121067	EGFR	TRCN0000078448	HDC
TRCN0000051342	GGH		HRH1
TRCN0000155335	ZW10	TRCN0000056619	TH
TRCN0000021604	NR1H4	TRCN0000121067	EGFR
TRCN0000000501	CHEK1	TRCN0000044138	CNGA1
TRCN0000056848	TLR3	TRCN0000039634	EGFR
TRCN0000060003	TCN2	TRCN0000051342	GGH
TRCN0000047354	RASD1	TRCN0000155335	ZW10
TRCN0000152366	B3GAT1	TRCN0000199796	CHEK1
TRCN0000160294	NPC1L1	TRCN0000199993	ERBB3
TRCN0000003671	CTSG	TRCN0000003755	TP53
TRCN0000036544	AKR1C1	TRCN0000074359	TUBB
TRCN0000046744	DPYS	TRCN0000008816	GNRHR
		TRCN0000014135	PTGER1
**Total**	**30**	TRCN0000083188	SFTPC
		TRCN0000038972	VKORC1
		TRCN0000014134	PTGER1
		TRCN0000046419	AKR1A1
		TRCN0000014451	LTB4R
		TRCN0000009271	OPRL1
		TRCN0000018917	IKBKB
		TRCN0000003753	TP53
		TRCN0000033500	BCL2L1
		TRCN0000152366	B3GAT1
		TRCN0000057762	ITGA2B
		TRCN0000046856	ACHE
		TRCN0000009971	HCK
		TRCN0000039747	CDK6
		TRCN0000051244	ARSE
		TRCN0000004842	HDAC6
		TRCN0000005956	MLL
		TRCN0000083190	SFTPC
		TRCN0000199622	INSR
		TRCN0000034909	NAT2
		TRCN0000004905	BLM
		TRCN0000008092	AGTR1
		TRCN0000222166	MYST4
		TRCN0000166735	MOAP1
		TRCN0000161240	NISCH
		TRCN0000018331	IGF1R
		TRCN0000150938	ZW10
		TRCN0000199601	CAMKK1
		TRCN0000001994	NTRK1
		TRCN0000004828	HDAC3
		TRCN0000197158	HDAC11
		TRCN0000056569	ALOX5
		TRCN0000199323	MTOR
		TRCN0000003234	ITGB3
		TRCN0000010369	KRAS
		TRCN0000009459	OXTR
		**Total**	**67**

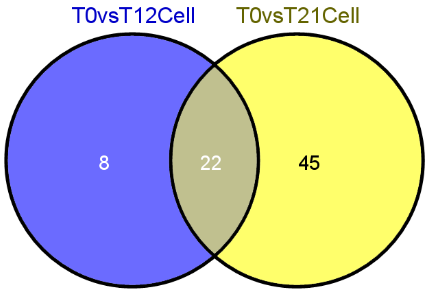

Cells + DMSO

T0 vs T12		T0 vs T21	
hpid	*gene*	*hpid*	*gene*
TRCN0000045929	FPGS	TRCN0000045929	FPGS
TRCN0000004828	HDAC3	TRCN0000004828	HDAC3
TRCN0000056848	TLR3	TRCN0000056848	TLR3
TRCN0000117932	KNTC1	TRCN0000117932	KNTC1
TRCN0000044140	CNGA1	TRCN0000006988	STK36
TRCN0000195066	BRAF	TRCN0000044140	CNGA1
TRCN0000000861	PRKAA1	TRCN0000000861	PRKAA1
TRCN0000197098	CDK4	TRCN0000197098	CDK4
TRCN0000121193	IGF1R	TRCN0000121193	IGF1R
TRCN0000060003	TCN2	TRCN0000060003	TCN2
TRCN0000083189	SFTPC	TRCN0000195487	FRK
TRCN0000195487	FRK	TRCN0000057762	ITGA2B
TRCN0000047354	RASD1	TRCN0000001028	HSP90AA1
TRCN0000002814	PPP3CB	TRCN0000047354	RASD1
TRCN0000036544	AKR1C1	TRCN0000053951	S100A13
TRCN0000196538	ATR	TRCN0000051244	ARSE
TRCN0000058753	IL2RB	TRCN0000002814	PPP3CB
TRCN0000221544	MTOR	TRCN0000001625	STK4
TRCN0000003671	CTSG	TRCN0000196538	ATR
TRCN0000078448	HDC	TRCN0000058753	IL2RB
TRCN0000046419		TRCN0000221544	MTOR
TRCN0000044138	CNGA1	TRCN0000003671	CTSG
TRCN0000159815	NPC1L1	TRCN0000009065	HRH1
TRCN0000051342	GGH	TRCN0000074361	TUBB
TRCN0000004905	BLM	TRCN0000121067	EGFR
TRCN0000196844	BRAF	TRCN0000046419	AKR1A1
TRCN0000039916	MAP2K4	TRCN0000044138	CNGA1
TRCN0000083188	SFTPC	TRCN0000159815	NPC1L1
TRCN0000053951	S100A13	TRCN0000051342	GGH
TRCN0000034818	UMPS	TRCN0000155335	ZW10
TRCN0000001028	HSP90AA1	TRCN0000036243	COMT
TRCN0000152366	B3GAT1	TRCN0000001994	NTRK1
TRCN0000045664	TYMS	TRCN0000043299	SLC15A1
TRCN0000199622	INSR	TRCN0000221543	MTOR
TRCN0000008092	AGTR1	TRCN0000063370	GRIN1
TRCN0000050453	PPAP2B	TRCN0000018331	IGF1R
TRCN0000064757	FADS2	TRCN0000039748	CDKN2A
TRCN0000014134	PTGER1	TRCN0000033500	BCL2L1
TRCN0000009271	OPRL1	TRCN0000005956	MLL
		TRCN0000014134	PTGER1
**Total**	**39**	TRCN0000046744	DPYS
		TRCN0000036324	FDFT1
		TRCN0000003755	TP53
		TRCN0000034818	UMPS
		TRCN0000003234	ITGB3
		TRCN0000083188	SFTPC
		TRCN0000195066	BRAF
		TRCN0000009271	OPRL1
		TRCN0000199323	MTOR
		TRCN0000039003	DHFR
		TRCN0000000916	BMP2K
		TRCN0000161240	NISCH
		TRCN0000039916	MAP2K4
		TRCN0000199622	INSR
		TRCN0000166735	MOAP1
		TRCN0000219684	MAP2K1
		TRCN0000014135	PTGER1
		TRCN0000121325	PLK1
		TRCN0000009971	HCK
		TRCN0000038956	DCXR
		TRCN0000014451	LTB4R
		TRCN0000045664	TYMS
		TRCN0000003753	TP53
		TRCN0000018917	IKBKB
		TRCN0000008899	MLNR
		TRCN0000152366	B3GAT1
		TRCN0000056569	ALOX5
		TRCN0000036145	BHMT
		TRCN0000021617	RXRA
		TRCN0000040093	APC
		TRCN0000219689	FRK
		TRCN0000150938	ZW10
		TRCN0000004842	HDAC6
		**Total**	**73**

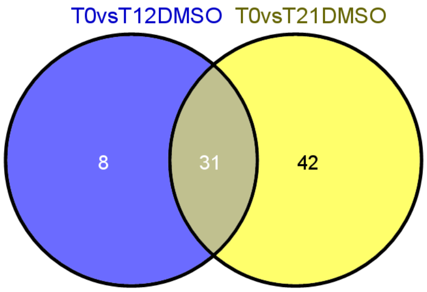

Cells + methanol

T0 vs T12		T0 vs T21	
hpid	*gene*	*hpid*	*gene*
TRCN0000045929	FPGS	TRCN0000056848	TLR3
TRCN0000117932	KNTC1	TRCN0000117932	KNTC1
TRCN0000195066	BRAF	TRCN0000006988	STK36
TRCN0000000861	PRKAA1	TRCN0000044140	CNGA1
TRCN0000083189	SFTPC	TRCN0000000861	PRKAA1
TRCN0000195487	FRK	TRCN0000121193	IGF1R
TRCN0000001028	HSP90AA1	TRCN0000083189	SFTPC
TRCN0000047354	RASD1	TRCN0000195487	FRK
TRCN0000002814	PPP3CB	TRCN0000001028	HSP90AA1
TRCN0000001625	STK4	TRCN0000002814	PPP3CB
TRCN0000058753	IL2RB	TRCN0000058753	IL2RB
TRCN0000078448	HDC	TRCN0000221544	MTOR
TRCN0000044138	CNGA1	TRCN0000003671	CTSG
TRCN0000003100	FYN	TRCN0000078448	HDC
TRCN0000083188	SFTPC	TRCN0000121067	EGFR
TRCN0000199796	CHEK1	TRCN0000046419	AKR1A1
TRCN0000001994	NTRK1	TRCN0000044138	CNGA1
TRCN0000056848	TLR3	TRCN0000051342	GGH
TRCN0000155335	ZW10	TRCN0000197222	CDK16
TRCN0000152366	B3GAT1	TRCN0000003755	TP53
TRCN0000121193	IGF1R	TRCN0000034818	UMPS
TRCN0000006076	DGUOK	TRCN0000073304	HUWE1
TRCN0000221544	MTOR	TRCN0000083188	SFTPC
TRCN0000006988	STK36	TRCN0000195066	BRAF
TRCN0000045664	TYMS	TRCN0000197236	CDK2
TRCN0000001025	HSP90AA1	TRCN0000003091	CPT2
TRCN0000060003	TCN2	TRCN0000033500	BCL2L1
TRCN0000121067	EGFR	TRCN0000045664	TYMS
		TRCN0000166735	MOAP1
		TRCN0000001593	FGR
**Total**	**29**	TRCN0000003991	TOP1
		TRCN0000197098	CDK4
		TRCN0000004905	BLM
		TRCN0000152366	B3GAT1
		TRCN0000050453	PPAP2B
		TRCN0000005956	MLL
		TRCN0000057762	ITGA2B
		TRCN0000199323	MTOR
		TRCN0000074361	TUBB
		TRCN0000121325	PLK1
		TRCN0000009971	HCK
		TRCN0000034909	NAT2
		TRCN0000221543	MTOR
		TRCN0000010369	KRAS
		TRCN0000046744	DPYS
		TRCN0000047354	RASD1
		TRCN0000199993	ERBB3
		TRCN0000051244	ARSE
		TRCN0000014134	PTGER1
		TRCN0000155335	ZW10
		TRCN0000038956	DCXR
		TRCN0000039634	EGFR
		**Total**	**52**

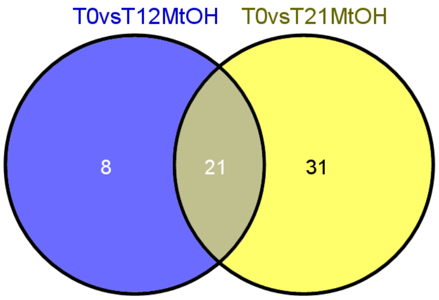

Figure S4 List of toxic hairpins. Based on the scatter plots represented in Figure S3, a list of toxic hairpins (i.e. hairpins that drop out of the minipool overtime) was established. Venn diagrams show the overlap between hairpins missing when comparing T0vsT12 and T0vsT21, for the same culture conditions.

A.

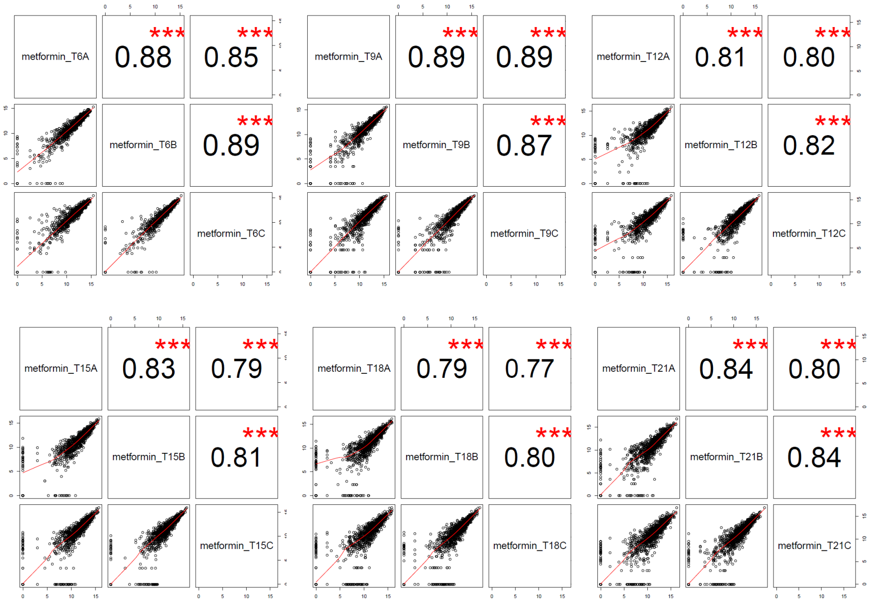

B.

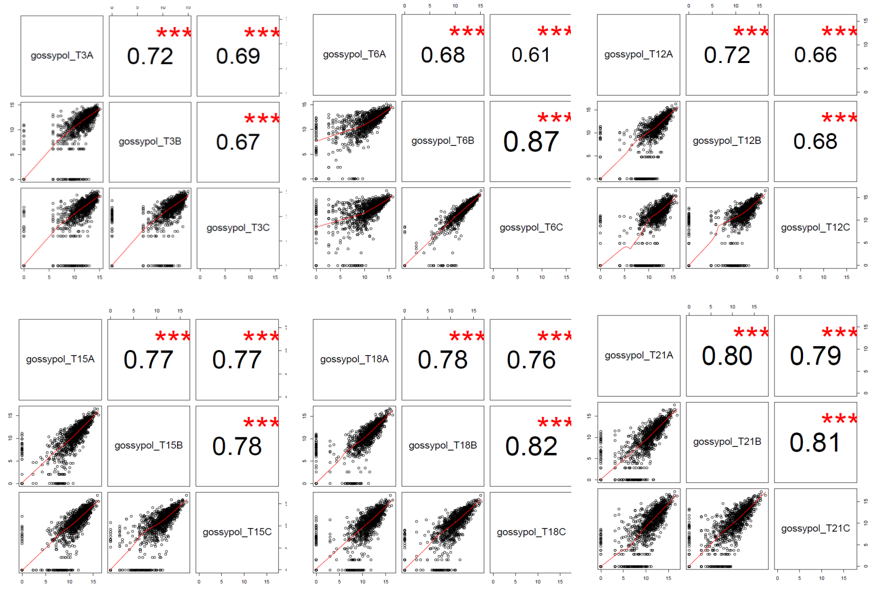

Figure S5 Correlation plots between triplicate (A, B, and C) of metformin (A.) and gossypol (B.) screen. Normalized Log2 of sequencing counts of each time point (T) has been stated. For each drug, the triplicate screens showed a high correlation (e.g. $r^2 \geq 0.8$, day 21) demonstrating a consistent hairpin drop out among triplicates and over time.

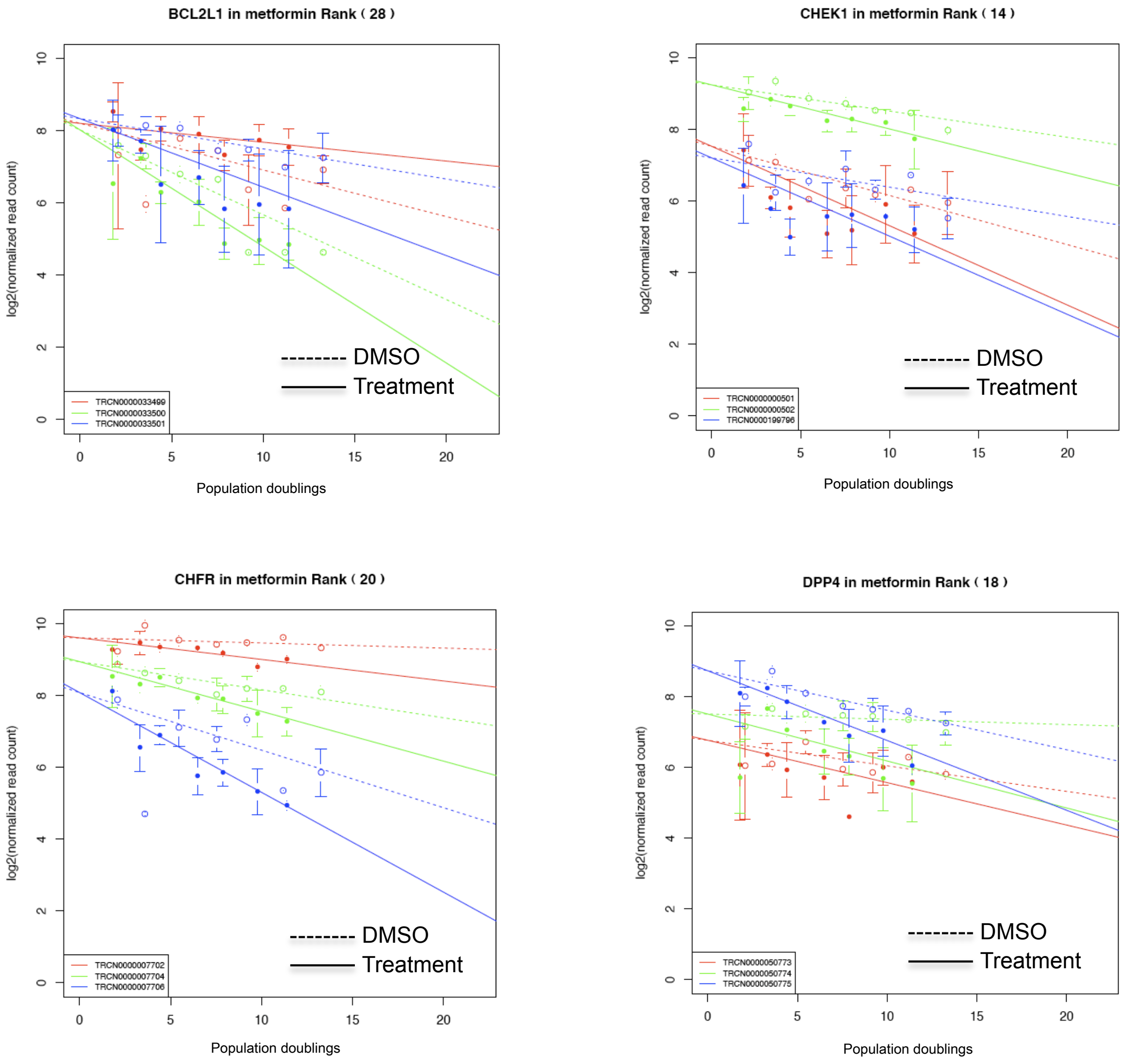

Figure S6 Linear plots of an individual gene that was knocking down by shRNA in A549 cells cultured in the presence of metformin are BCL2L1, CHEK1, CHFR and DPP4 respectively. Y-axis exhibits the log2 of normalization sequencing counts and X-axis demonstrates the doubling time in the screen. The combination of shRNAs and metformin treatment shows the decreasing in the signal read (solid line) compared to vehicle control (dash line).

	Q1: FL1-H–, FL2-H+	Q2: FL1-H+, FL2-H+	Q3: FL1-H+, FL2-H–	Q4: FL1-H–, FL2-H–
A549_cellonly	0.397	0	0	99.6
A549_annexinV	0.705	2.68	1.41	95.2
A549_PI	3.74	0	0	96.3
A549_NoDrug	3.5	0.937	0.134	95.4
A549+5uM_gossypol	10.4	2.28	0.178	87.2
RFP	4.7	5.01	1.29	89
RFP+5uM_gossypol	12	5.6	0.298	82.1
KCND3	9.11	7.59	0.623	82.7
KCND3+5uM_gossypol	22.9	17.1	0.299	59.7
DBI_hp1	11.1	7.14	0.848	80.9
DBI_hp1+5uM_gossypol	19.7	5.71	0.327	74.2
**DBI_hp2**	**16.6**	**8.58**	**0.644**	**74.2**
**DBI_hp2+5uM_gossypol**	**27.5**	**40.4**	**0.224**	**31.8**
**CRTAM**	**9.97**	**8.13**	**0.657**	**81.2**
**CRTAM+5uM_gossypol**	**13.2**	**30.4**	**0.454**	**55.9**
CHAT_hp1	4.63	4.38	0.67	90.3
CHAT_hp1+5uM_gossypol	12.5	10.7	0.617	76.1
**CHAT_hp2**	**8.84**	**9.67**	**1.24**	**80.3**
**CHAT_hp2+5uM_gossypol**	**20.7**	**21**	**0.453**	**57.8**
**ErbB2_hp1**	**10.6**	**6.02**	**0.385**	**83**
**ErbB2_hp1+5uM_gossypol**	**18.7**	**16.7**	**0.309**	**64.3**
**ErbB2_hp2**	**13.3**	**12**	**1.01**	**73.7**
**ErbB2_hp2+5uM_gossypol**	**27.1**	**26.6**	**0.415**	**45.9**
**PTK2_hp1**	**10.9**	**4.37**	**0.283**	**84.4**
**PTK2_hp1+5uM_gossypol**	**18.9**	**15.9**	**0.221**	**65.1**
**PTK2_hp2**	**9.12**	**4.57**	**0.114**	**86.2**
**PTK2_hp2+5uM_gossypol**	**15.8**	**19.8**	**0.824**	**63.7**
**PTK2_hp3**	**11.3**	**16.1**	**1.65**	**70.9**
**PTK2_hp3+5uM_gossypol**	**23.2**	**27**	**0.712**	**49**
**BRCA2_hp1**	**9.1**	**8.5**	**0.447**	**82**
**BRCA2_hp1+5uM_gossypol**	**13.1**	**28.7**	**0.876**	**57.3**
**BRCA2_hp2**	**7.58**	**4.56**	**0.383**	**87.5**
**BRCA2_hp2+5uM_gossypol**	**12.5**	**17.1**	**0.918**	**69.5**

	Q1: FL1-H–, FL2-H+	Q2: FL1-H+, FL2-H+	Q3: FL1-H+, FL2-H–	Q4: FL1-H–, FL2-H–
A549cell only	0.183	0.0203	0	99.8
A549_PI	2.66	0	0	97.3
A549_AnnexinV	0.258	2.68	1.15	95.9
A549_NoDrug	8.45	3.84	0.736	87
A549+5uM_gossypol	21.9	3.97	0.119	74
LacZ_NoDrug	13.6	2.91	0.299	83.2
LacZ+5uM_gossypol	14.3	5.62	0.913	79.1
Luciferase_NoDrug	12.7	10.3	1.71	75.3
Luciferase+5uM_gossypol	27.3	8.2	0.413	64.1
RFP_NoDrug	12.7	12.1	2.19	73
RFP+5uM_gossypol	17.8	10.2	1.22	70.8
**CHEK1**	**9.54**	**9.39**	**0.835**	**80.2**
**CHEK1+5uM_gossypol**	**13.8**	**29.4**	**0.881**	**55.9**
MCL1	18.7	19.9	2.21	59.2
MCL1+5uM_gossypol	20.4	19.1	1.94	58.6
**HSPA8_hp1**	**11.1**	**19.4**	**3.95**	**65.6**
**HSPA8_hp1+5uM_gossypol**	**22.8**	**10.8**	**0.771**	**65.6**
**HSPA8_hp2**	**11**	**7.85**	**0.972**	**80.2**
**HSPA8_hp2+5uM_gossypol**	**16.3**	**23.8**	**0.841**	**59.1**
CHFR_hp1	11.5	7.45	1.36	79.7
CHFR_hp1+5uM_gossypol	18.9	13.2	0.674	67.1
CHFR_hp2	16	10.5	0.811	72.7
CHFR_hp2+5uM_gossypol	29.2	21.2	0.432	49.1
**CHFR_hp3**	**17.1**	**7.15**	**0.435**	**75.3**
**CHFR_hp3+5uM_gossypol**	**23.5**	**23.7**	**0.495**	**52.3**
**HUWE1_hp1**	**8.1**	**9.37**	**1.05**	**81.5**
**HUWE1_hp1+5uM_gossypol**	**12.6**	**19.8**	**0.567**	**67**
**HUWE1_hp2**	**11.5**	**7.95**	**0.887**	**79.7**
**HUWE1_hp2+5uM_gossypol**	**12.2**	**22.7**	**0.877**	**64.2**
**HUWE1_hp3**	**14.2**	**21.9**	**2.74**	**61.2**
**HUWE1_hp3+5uM_gossypol**	**24.1**	**36.6**	**0.416**	**38.9**
**HDAC2_hp1**	**18.1**	**27.5**	**2.44**	**51.9**
**HDAC2_hp1+5uM_gossypol**	**20.6**	**40**	**1.11**	**38.3**
**HDAC2_hp2**	**10.6**	**9.6**	**0.583**	**79.3**
**HDAC2_hp2+5uM_gossypol**	**19.3**	**16.2**	**0.584**	**63.9**
WRN_hp1	13.7	8.71	0.672	77
WRN_hp1+5uM_gossypol	15	12.4	0.627	71.9
**WRN_hp2**	**11**	**6.89**	**0.562**	**81.5**
**WRN_hp2+5uM_gossypol**	**15.9**	**15.6**	**0.768**	**67.7**
BRCA1_hp1	18.3	11.1	1.24	69.4
BRCA1_hp1+5uM_gossypol	27.9	12.2	0.828	59.1
BRCA1_hp2	11.9	13.8	1.38	73
BRCA1_hp2+5uM_gossypol	19.2	19.4	0.627	60.8

Figure S7 After 3 days of gossypol treatment, cells were trypsinized and stained with FITCconjugated annexin V (FL1 detection, 488nm/515-545nm) and propidium iodide (FL2 detection, 488nm/564-606nm) in order to assess cell viability by flow cytometry. Percentage of dead/dying (FL1-/FL2+ and FL1+/FL2+), apoptotic (FL1+/FL2-) and living cells (FL1-/FL2-) in the presence of gossypol in the A549 cell lines silenced for the indicated candidate genes. Cells without hairpin or with a hairpin directed against luciferase, lacZ and RFP are used as negative controls. Individual shRNAs for selected genes are indicated (1 to 3 hairpins per genes). Hairpins in bold confere an increased of toxicity superior to the controls when in combination with gossypol.

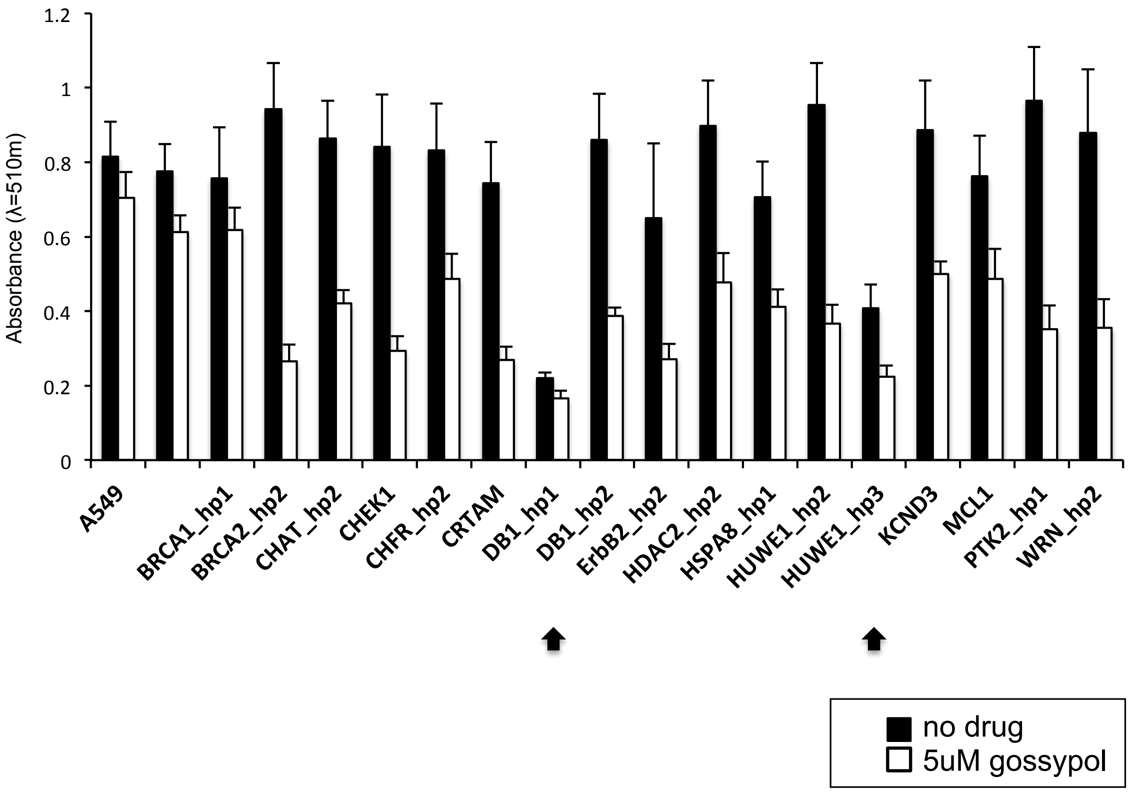

Figure S8 Effect of the hairpins targeting the gossypol potential hit genes on A549 viability. Infected A549 cells were seeded in 96-well plates at 2,200 cells/well in 200μL of medium. Gossypol was diluted first in DMSO then in RPMI, with the final DMSO concentration not exceeding 1%. After 72 hours of incubation, cell viability was measured by sulforhodamine B (SRB) viability assay. Dye concentration was determined using a microplate reader at a wavelength of 510 nm (Bio Tek Synergy 2). Intensities of the signal are proportional to the amount of stained protein and reflect the relatives cell densities. Arrows indicate toxic hairpins in the absence of drug. Errors bars show s.e.m. (n=3).

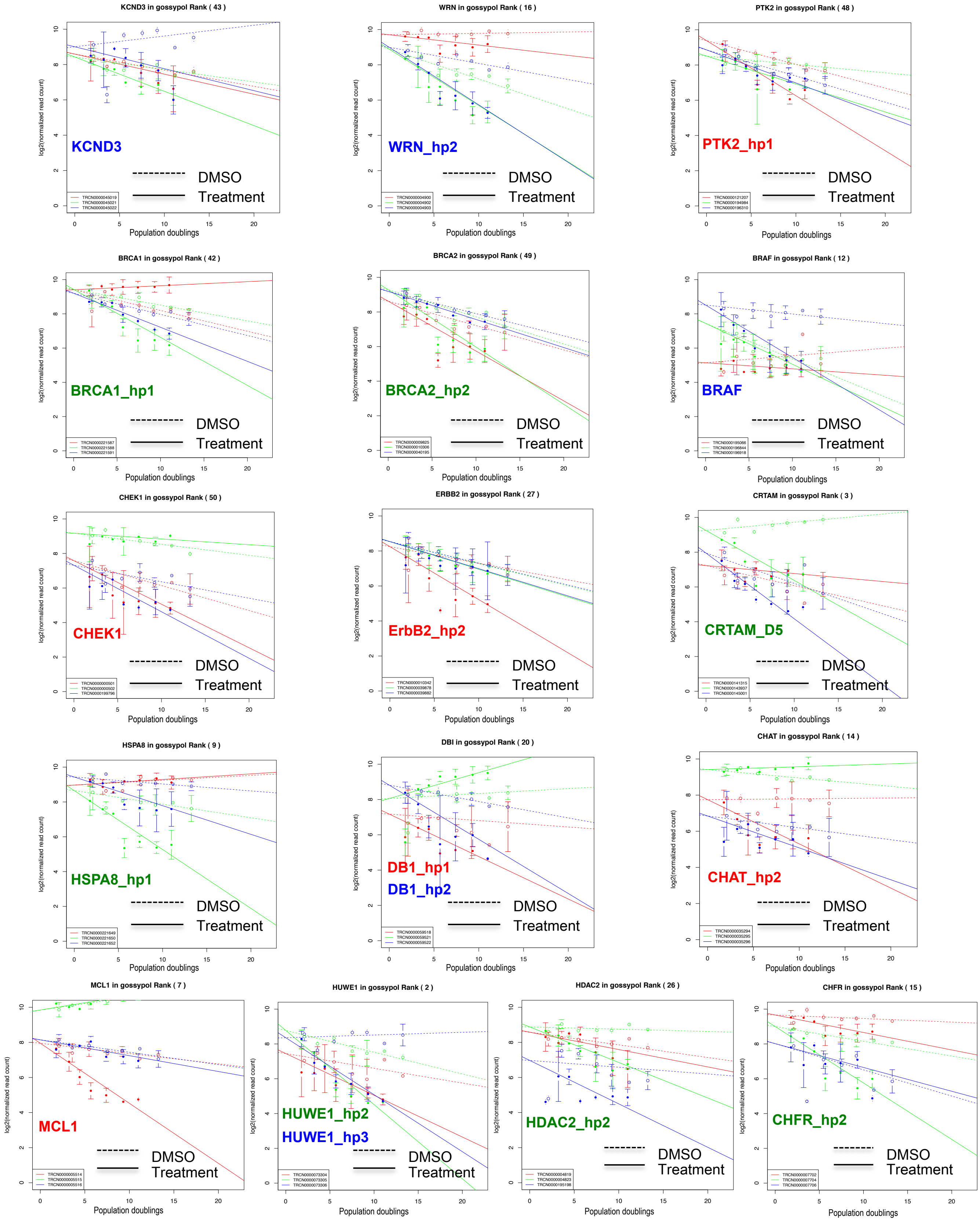

Figure S9 Potential gossypol targets are shown in sixteen linear plots. As in Figure S6, depletion of the following genes, KCND3, WRN, PTK2, BRCA1, BRCA2, BRAF, CHEK1, ERBB2, CRTAM, HSPA8, DBI, CHAT, MCL1, HUWE1, HDAC2 and CHFR confer sensitivity to gossypol treatment in A549 as shown solid line.

Class	Compound	Screening concentration (uM)	GI50	Reference	Clinical application
Antiproliferative	hydroxy urea	90	526.8uM in A549	NCI-60	antineoplastic
	etoposide	0.35	0.951uM in A549	NCI-60	antineoplastic
	camptothecin (nM)	5	19.4uM in A549	NCI-60	antineoplastic
	doxorubicin (nM)	6	0.049uM in A549	NCI-60	antineoplastic
	vincristine (nM)	6.5	10.8uM in A549	NCI-60	antineoplastic
	amsacrine	12.5	17.6uM in A549	NCI-60	antineoplastic
	methotrexate	0.03	0.023uM in A549	[1]	antineoplastic
	taxol (nM)	1	0.789uM in A549	NCI-60	antineoplastic
	gossypol	5	40uM in K562 (leukaemia), 44uM in H69 (small cell lung), 26uM in SK-mel-19 (melanoma)	[2]	antineoplastic
	methyl methanesulfonate (%)	0.0012			antineoplastic
	vorinostat	1.25	2.5 to 7.5uM in prostate cell lines LNCaP, PC-3 and TSU-Pr1	[4]	antineoplastic
	gefitinib	4	4.5uM in A549	[5]	antineoplastic
	mitomycin C	9	0.196uM in A549	NCI-60	antineoplastic
	imatinib	10	15uM in SMS-KCRN (neuroblastoma)	[6]	antineoplastic
	marimastat (BB-2516)	16	0.2uM in HT-1080/MT1 (fibrosarcoma)	[7]	antineoplastic
	digoxin (nM)	12	70nM in MDA-MB-231 (breast cancer)	[9]	heart treatment
	cyclosporin A	0.45	8.29uM in A549	NCI-60	immunosuppressive
	mycophenolic acid	0.4	41uM in A549	NCI-60	immunosuppressive
	rapamycin (nM)	1	2.93uM in A549	NCI-60	immunosuppressive
	tacrolimus	22	30uM in HEK293	[10]	immunosuppressive
	rotenone	0.1	10uM in SH-Sy5Y (neuroblastoma)	[11]	insecticide, and pesticide
	roscovitine	3	Between 13 to 36uM in various diffuse large B-cell lymphoma cell lines	[12]	treatment of non-small cell lung cancer (NSCLC), leukemia, HIV infection, herpes simplex infection
	mitaplatin	1	3.47uM in KB-3-1 (epidermoid tumor) , 6.84uM in BEL 7404 (hepatoma cell line)	[3]	antineoplastic
	retinoic acid	25	Non-small cell lung cancer: 70uM in A549, 65uM in H460, 80uM in H157	[8]	antineoplastic
Non antiproliferative	racecadotril	90			antidiarrhea
	artemisinin	22.5	16.8uM (Human Foreskin Fibroblasts)	[13]	anti-infective
	sulfasalazine	900	500uM in U87 (glioblastoma)	[14]	anti-inflammatory
	indomethacin	95	92.15uM (U138-MG glioma)	[15]	anti-inflammatory (NSAID)
	naproxen	96	1450uM in HCA-7 (colon cancer)	[16]	anti-inflammatory (NSAID)
	ibuprofen	450	900uM in HCT-116 (colon cancer)	[17]	anti-inflammatory (NSAID)
	salicylate	700	1867uM HT-29 (colon cancer)	[18]	anti-inflammatory (NSAID)
	verapamil	35	45uM Te671 (medulloblastoma)	[19]	antiarrhythmic, treatment of angina, hypertension
	tigecycline	50	3 to 8uM in leukemia cell lines	[20]	antibiotic
	erythromycin	200	712uM in CCL13 (human liver cell line)	[21]	antibiotic
	warfarin	120	184uM in mouse D3 embryonic stem cells	[22]	anticoagulant
	metformin	85	2000 to 3000uM in thyroid cancer cell lines	[23]	antihyperglycemic
	orlistat	11.2	7.5 to 21.5uM in breast cancer cell lines	[24]	antilipemic
	lovastatin	16.5	10uM in mesothelioma cell line	[25]	antilipemic
	trifluoperazine	10	Non-small cell lung cancer: 14uM in CL83, 8.5uM in CL152, 15uM in H1975	[26]	antipsychotic
	haloperidol	17.5	Prostate cancer cell lines: 177uM for LNCaP, 208uM for PC3	[27]	antipsychotic
	clozapine	16.5	250uM in hepatocytes	[28]	antipsychotic
	methimazole	400			antithyroid
	isoproterenol	27.5			asthma and bronchospasm
	aminophylline	225	428uM CEM-GH (leukemia)	[29]	asthma and bronchospasm
	propanolol	33	11.6uM in A549	NCI-60	bronchospasm and heart treatment
	mancozeb	42.5	Colon cancer cell lines: 200uM in HT-29, 80uM in Caco2	[30]	fungicide
	allopurinol	250			hyperuricemia treatment
	sildenafil	46	4.1uM in B-cell from CLL patient	[32]	pulmonary hypertension
	naltrexone	80			treatment of alcohol dependence
	MPTP	210	400uM in N2a (mouse neuroblastoma)	[31]	neurotoxin, parkinson disease

In gray: non FDA approved drugs (preclinicals, tools,…)

Table S1 Compounds used in the screens and their published GI_{50}s (drug concentrations that causes 50% cell growth inhibition). Values from the NCI-60 where retrieved from the NCI/NIH Developmental Therapeutics Program website (dtp.nci.nih.gov).

Table S2 List of the calculated hairpin interaction values for the 50 tested drugs

Table S3 Linear plots of the top hairpins for the 50 tested drugs. Based on their interaction values, we compiled the top shRNAs that rendered the cell hypersensitive to one of the 50 tested drugs.

Tables S2 and S3 are available for download at http://www.g3journal.org/lookup/suppl/doi:10.1534/g3.113.006437/-/DC1.

Class	Chemical	Primary hits	Reported known target	Rank #	TRC_hpID	Target Sequence	Percentage transcript remaining
Antiproliferative	etoposide	top 31	TOP1	28	TRCN0000003990	CAGAGTTGGATGGTCAGGAAT	4
			TOP2B	154	TRCN0000049284	GCAGCCTCTAATTGTGGCATT	83
			ABCB1	180	TRCN0000059683	CCGAACACATTGGAAGGAAAT	ND
		bot 29	TOP2A	1078	TRCN0000049278	GCCCAAGTGTTCTTTAGCTTT	ND
	camptothecin	top 54	TOP1	1039	TRCN0000003987	TGAAGGGCGAGTGAATCTAAG	6
				1057	TRCN0000003990	CAGAGTTGGATGGTCAGGAAT	4
		bot 15		1066	TRCN0000003991	GCTTCTCTAGTCCACCACAAA	24
	doxorubicin	top 45	TOP1	216	TRCN0000003991	GCTTCTCTAGTCCACCACAAA	24
	amsacrine	top 46	TOP1	6	TRCN0000003990	CAGAGTTGGATGGTCAGGAAT	4
				7	TRCN0000003987	TGAAGGGCGAGTGAATCTAAG	ND
			TOP2B	120	TRCN0000134040	CCAGAAACTTTCCTTGCTAAT	27
		bot 17	TOP2A	1098	TRCN0000049278	GCCCAAGTGTTCTTTAGCTTT	21
				1097	TRCN0000049279	GCCTGATTTGTCTAAGTTTAA	19
				1083	TRCN0000049280	GCTCCAAATCAATATGTGATT	21
	methotrexate	top 44	FPGS	15	TRCN0000045928	CCAGTTTGACTATGCCGTCTT	37
			FPGS	73	TRCN0000045930	TCAGACACAGTTGGAAGCCAT	68
			SLC22A6	116	TRCN0000043254	CCCTTTCTCAATGGCACAGAA	ND
			ABCC3	219	TRCN0000059406	GCACTGCTGCACAACAAGATA	ND
	taxol	top 40	ITGB3	70	TRCN0000003236	CCTTAGCCTTTGTCCCAGAAT	11
	gossypol	top 68	MCL1	7	TRCN0000005514	GCTGTGTTAAACCTCAGAGTT	ND
			BCL2L11	123	TRCN0000001051	ATGGTTATCTTACGACTGTTA	33
			BCL2	189	TRCN0000010303	TGGATGACTGAGTACCTGAAC	33
	vorinostat	top 65	HDAC9	16	TRCN0000196384	GAGCAGTTAATAGGCTTTAAA	10
			HDAC11	43	TRCN0000197158	GTTTCTGTTTGAGCGTGTGGA	17
			HDAC4	61	TRCN0000004830	CGTGGGTTTCAACGTCAACAT	57
			HDAC5	63	TRCN0000004838	GCCGGGTTTGATGCTGTTGAA	41
			HDAC2	159	TRCN0000004823	GCAAATACTATGCTGTCAATT	13
	gefitinib	top 61	RIPK2	51	TRCN0000006349	GCACCATTTCTGGATCTCAAA	92
				69	TRCN0000006347	CCAGGCTTAATTGCCCTACAA	98
			SRC	112	TRCN0000195339	CATCCTCAGGAACCAACAATT	18
	imatinib	top 27	MAPK8	8	TRCN0000194860	CAGTAAGGACTTACGTTGAAA	13
			FYN	18	TRCN0000003100	CTTACCGATCTGTCTGTCAAA	57
			FIP1L1	41	TRCN0000074422	CGAATGGGACTTGAAGTTATA	38
			ABL1	48	TRCN0000121279	CCTCAGTTCGGTGAAGGAAAT	10
				113	TRCN0000121281	ACTTGGTGAAGGTAGCTGATT	15
			ETV6	89	TRCN0000003854	GTTGTTAGTATCATGGTGTTT	12
			BCR	215	TRCN0000195722	CCCTCACTGTTGTATCTTGAA	15
	marimastat	top 34	ADAM17	109	TRCN0000052168	CCAGCAGCATTCGGTAAGAAA	23
	digoxin	top 70	SLCO4C1	170	TRCN0000038309	GCCCTGCTATTGGCTATGTAT	ND
	cyclosporinA	top 30	ABCB1	46	TRCN0000059683	CCGAACACATTGGAAGGAAAT	ND
	mycophenolic	top 18	IMPDH1	204	TRCN0000026570	CCTGAAGAAGAACCGAGACTA	ND
	rapamycin	top 27	MTOR	48	TRCN0000221544	GCTGTGCTACACTACAAACAT	ND
			ABCB1	97	TRCN0000059685	CGACAGAATAGTAACTTGTTT	ND
			FKBP3	72	TRCN0000063539	CCACTTGGTTACAGCCTATAA	2
			FKBP4	131	TRCN0000152554	GCATGGAGAAAGGAGAACATT	0
			FKBP5	225	TRCN0000000235	CGAAGGAGCAACAGTAGAAAT	5
		bot 32	FKBP1A	1,096	TRCN0000005951	GCCAAACTGACTATATCTCCA	5
				1,097	TRCN0000005953	GAGAGCCAAACTGACTATATC	15
				1,098	TRCN0000005949	AGAGAGCCAAACTGACTATAT	4
	tacrolimus	top 31	IL2RG	29	TRCN0000058470	ACGACAATTCTGACGCCCAAT	ND
			PPP3CB	138	TRCN0000002814	CCCGGAAAGAAATCATAAGAA	8
			MTOR	141	TRCN0000221543	GCAACCCTTCTTTGACAACAT	ND
			FKBP1A	153	TRCN0000005951	GCCAAACTGACTATATCTCCA	5
			IL2RA	204	TRCN0000059165	GCCACAGAGAGAATTTATCAT	ND
Non antiproliferative	racecadotril	top 53	MME	217	TRCN0000046823	CCAGGCAATTTCAGGATTATT	8
	salfasalazine	top 57	NAT2	146	TRCN0000034912	CTGGTGATGGATCCCTTACTA	ND
	ibuprofen	top 53	CXCR1	129	TRCN0000009132	GCCACTGAGATTCTGGGATTT	ND
	salicylate	top 33	SLC22A6	78	TRCN0000043253	GCAGGGATATATCCCAGGAAA	ND
	verapamil	top 62	CYP3A4	41	TRCN0000064225	CCCTGAAAGATTCAGCAAGAA	ND
			ABCB1	228	TRCN0000059684	GCAGCAATTAGAACTGTGATT	ND
	warfarin	top 41	VKORC1	2	TRCN0000038972	GCTCTCGCTCTACGCGCTGCA	7
			CYP3A4	22	TRCN0000064225	CCCTGAAAGATTCAGCAAGAA	ND
	lovastatin	top 49	CYP3A4	126	TRCN0000064225	CCCTGAAAGATTCAGCAAGAA	ND
			HMGCR	174	TRCN0000046448	GCAGTGATAAAGGAGGCATTT	79
	clozapine	top 50	CYP3A4	90	TRCN0000064225	CCCTGAAAGATTCAGCAAGAA	ND
			HRH1	119	TRCN0000011675	CCCTGGAAATTCTGCCTTATT	34
			DRD4	206	TRCN0000014453	GCCCGTACTGTGCGGCCTCAA	ND
	methimazole	top 34	TPO	138	TRCN0000045969	CGACAAGATCCTGGACTTGTA	91
	isoproterenol	top 63	ADCY10	182	TRCN0000078371	CCTGTTCAAGTATTCCATTAA	58
	aminophylline	top 48	ADORA2A	91	TRCN0000008042	CCACACCAATTCGGTTGTGAA	51
			ADORA1	145	TRCN0000008038	CCTTACCTACATTGCCATCTT	ND
	allopurinol	top 34	XDH	204	TRCN0000028098	GCAACTTTACTGTTTCAGAAA	ND

Table S4 List of potential targets for each of the 28 drugs with reported targets. In grey are the reported targets belonging to the top hits. Percentage of remaining transcript calculated by qPCR in A549, MCF7 or HEK293T/17 are also reported (source CCBR-OICR Lentiviral Technology Cancer).

Tables S5 and S6   Normalized count number for individual hairpin in the A549s cultured in presence of indicated drugs. In order to compare control and drug experiments, the total sum of all counts for each sample was normalized to be the same. A count number equal to zero represents a missing hairpin. Results for each triplicate experiments (as replicates A, B and C) at all time points (T0, 3, 6, 9, 12, 15, 18, 21) are shown.

Table S7   Percentage of remaining gene expression assessed by qPCR in A549, MCF7 or HEK293T/17 for each hairpins of the minipool collection (source CCBR-OICR Lentiviral Technology Cancer).

Tables S5-S7 are available for download at http://www.g3journal.org/lookup/suppl/doi:10.1534/g3.113.006437/-/DC1.

1. Rosowsky, A., et al., *Analogues of N alpha-(4-amino-4-deoxypteroyl)-N delta-hemiphthaloyl-L-ornithine (PT523) modified in the side chain: synthesis and biological evaluation.* J Med Chem, 1997. **40**(3): p. 286-99.
2. Shelley, M.D., et al., *Structure-activity studies on gossypol in tumor cell lines.* Anticancer Drugs, 2000. **11**(3): p. 209-16.
3. Xue, X., et al., *Mitaplatin increases sensitivity of tumor cells to cisplatin by inducing mitochondrial dysfunction.* Mol Pharm, 2012. **9**(3): p. 634-44.
4. Butler, L.M., et al., *Suberoylanilide hydroxamic acid, an inhibitor of histone deacetylase, suppresses the growth of prostate cancer cells in vitro and in vivo.* Cancer Res, 2000. **60**(18): p. 5165-70.
5. Van Schaeybroeck, S., et al., *Chemotherapy-induced epidermal growth factor receptor activation determines response to combined gefitinib/chemotherapy treatment in non-small cell lung cancer cells.* Mol Cancer Ther, 2006. **5**(5): p. 1154-65.
6. Beppu, K., et al., *Effect of imatinib mesylate on neuroblastoma tumorigenesis and vascular endothelial growth factor expression.* J Natl Cancer Inst, 2004. **96**(1): p. 46-55.
7. Wolf, K., et al., *Compensation mechanism in tumor cell migration: mesenchymal-amoeboid transition after blocking of pericellular proteolysis.* J Cell Biol, 2003. **160**(2): p. 267-77.
8. Soriano, A.F., et al., *Synergistic effects of new chemopreventive agents and conventional cytotoxic agents against human lung cancer cell lines.* Cancer Res, 1999. **59**(24): p. 6178-84.
9. Prassas, I. and E.P. Diamandis, *Novel therapeutic applications of cardiac glycosides.* Nat Rev Drug Discov, 2008. **7**(11): p. 926-35.
10. Lamoureux, F., et al., *Quantitative proteomic analysis of cyclosporine-induced toxicity in a human kidney cell line and comparison with tacrolimus.* J Proteomics, 2011. **75**(2): p. 677-94.
11. Kim, H.J., et al., *Naringin Protects against Rotenone-induced Apoptosis in Human Neuroblastoma SH-SY5Y Cells.* Korean J Physiol Pharmacol, 2009. **13**(4): p. 281-5.
12. Lacrima, K., et al., *Cyclin-dependent kinase inhibitor seliciclib shows in vitro activity in diffuse large B-cell lymphomas.* Leuk Lymphoma, 2007. **48**(1): p. 158-67.
13. Arav-Boger, R., et al., *Artemisinin-derived dimers have greatly improved anti-cytomegalovirus activity compared to artemisinin monomers.* PloS one, 2010. **5**(4): p. e10370.
14. Robe, P.A., et al., *In vitro and in vivo activity of the nuclear factor-kappaB inhibitor sulfasalazine in human glioblastomas.* Clin Cancer Res, 2004. **10**(16): p. 5595-603.
15. Bernardi, A., et al., *Selective cytotoxicity of indomethacin and indomethacin ethyl ester-loaded nanocapsules against glioma cell lines: an in vitro study.* Eur J Pharmacol, 2008. **586**(1-3): p. 24-34.
16. Tavolari, S., et al., *Licofelone, a dual COX/5-LOX inhibitor, induces apoptosis in HCA-7 colon cancer cells through the mitochondrial pathway independently from its ability to affect the arachidonic acid cascade.* Carcinogenesis, 2008. **29**(2): p. 371-80.
17. Khwaja, F., et al., *Ibuprofen inhibits survival of bladder cancer cells by induced expression of the p75NTR tumor suppressor protein.* Cancer Res, 2004. **64**(17): p. 6207-13.
18. Hixson, L.J., et al., *Antiproliferative effect of nonsteroidal antiinflammatory drugs against human colon cancer cells.* Cancer Epidemiol Biomarkers Prev, 1994. **3**(5): p. 433-8.
19. Schmidt, W.F., et al., *Antiproliferative effect of verapamil alone on brain tumor cells in vitro.* Cancer Res, 1988. **48**(13): p. 3617-21.
20. Skrtic, M., et al., *Inhibition of mitochondrial translation as a therapeutic strategy for human acute myeloid leukemia.* Cancer Cell, 2011. **20**(5): p. 674-88.
21. Viluksela, M., P.J. Vainio, and R.K. Tuominen, *Cytotoxicity of macrolide antibiotics in a cultured human liver cell line.* J Antimicrob Chemother, 1996. **38**(3): p. 465-73.
22. Groebe, K., et al., *Unexpected common mechanistic pathways for embryotoxicity of warfarin and lovastatin.* Reprod Toxicol, 2010. **30**(1): p. 121-30.
23. Chen, G., et al., *Metformin inhibits growth of thyroid carcinoma cells, suppresses self-renewal of derived cancer stem cells, and potentiates the effect of chemotherapeutic agents.* J Clin Endocrinol Metab, 2012. **97**(4): p. E510-20.
24. Menendez, J.A., L. Vellon, and R. Lupu, *Antitumoral actions of the anti-obesity drug orlistat (XenicalTM) in breast cancer cells: blockade of cell cycle progression, promotion of apoptotic cell death and PEA3-mediated transcriptional repression of Her2/neu (erbB-2) oncogene.* Ann Oncol, 2005. **16**(8): p. 1253-67.
25. Rubins, J.B., et al., *Lovastatin induces apoptosis in malignant mesothelioma cells.* Am J Respir Crit Care Med, 1998. **157**(5 Pt 1): p. 1616-22.
26. Yeh, C.T., et al., *Trifluoperazine, an antipsychotic agent, inhibits cancer stem cell growth and overcomes drug resistance of lung cancer.* Am J Respir Crit Care Med, 2012. **186**(11): p. 1180-8.

27. Marrazzo, A., et al., *Antiproliferative activity of phenylbutyrate ester of haloperidol metabolite II [(+/-)-MRJF4] in prostate cancer cells.* Eur J Med Chem, 2011. **46**(1): p. 433-8.
28. Li, A.P., et al., *Cryopreserved human hepatocytes: characterization of drug-metabolizing enzyme activities and applications in higher throughput screening assays for hepatotoxicity, metabolic stability, and drug-drug interaction potential.* Chem Biol Interact, 1999. **121**(1): p. 17-35.
29. Ogawa, R., et al., *Inhibition of PDE4 phosphodiesterase activity induces growth suppression, apoptosis, glucocorticoid sensitivity, p53, and p21(WAF1/CIP1) proteins in human acute lymphoblastic leukemia cells.* Blood, 2002. **99**(9): p. 3390-7.
30. Hoffman, L. and D. Hardej, *Ethylene bisdithiocarbamate pesticides cause cytotoxicity in transformed and normal human colon cells.* Environ Toxicol Pharmacol, 2012. **34**(2): p. 556-73.
31. De Girolamo, L.A., A.J. Hargreaves, and E.E. Billett, *Protection from MPTP-induced neurotoxicity in differentiating mouse N2a neuroblastoma cells.* J Neurochem, 2001. **76**(3): p. 650-60.
32. Sarfati, M., et al., *Sildenafil and vardenafil, types 5 and 6 phosphodiesterase inhibitors, induce caspase-dependent apoptosis of B-chronic lymphocytic leukemia cells.* Blood, 2003. **101**(1): p. 265-9.
