# Supplementary material for: Miniature Short Hairpin RNA Screens to Characterize Antiproliferative Drugs
Source: G3 (Bethesda). 2013 Aug 1;3(8):1375–87. doi: 10.1534/g3.113.006437 (PMC3737177; doi:10.1534/g3.113.006437)
Supplement: Supporting Information [file supp_g3.113.006437_FigureS1.pdf]

	Symbol	GeneID	Approved Name
Carbohydrate metabolism	ACE	1636	angiotensin I converting enzyme (peptidyl-dipeptidase A) 1
	AKR1B1	231	aldo-keto reductase family 1, member B1 (aldose reductase)
	AKT1	207	v-akt murine thymoma viral oncogene homolog 1
	B3GAT1	27087	beta-1,3-glucuronyltransferase 1 (glucuronosyltransferase P)
	DCXR	51181	dicarbonyl/L-xylulose reductase
	INSR	3643	insulin receptor
	MGAM	8972	maltase-glucoamylase (alpha-glucosidase)
	MMP13	4322	matrix metallopeptidase 13 (collagenase 3)
	MMP3	4314	matrix metallopeptidase 3 (stromelysin 1, progelatinase)
	MMP7	4316	matrix metallopeptidase 7 (matrilysin, uterine)
Cell cycle	CCND2	894	cyclin D2
	KNTC1	9735	kinetochore associated 1
	MAD1L1	8379	MAD1 mitotic arrest deficient-like 1 (yeast)
	BRIP1	83990	BRCA1 interacting protein C-terminal helicase 1
	CDKN1A	1026	cyclin-dependent kinase inhibitor 1A (p21, Cip1)
Cell organization and biogenesis	ABL1	25	c-abl oncogene 1, receptor tyrosine kinase
	BBC3	27113	BCL2 binding component 3
	BCL2	596	B-cell CLL/lymphoma 2
	BCL2L1	598	BCL2-like 1
	BCL2L7	578	BCL2-antagonist/killer 1
	BLM	641	Bloom syndrome, RecQ helicase-like
	BRCA1	672	breast cancer 1, early onset
	BRCA2	675	breast cancer 2, early onset
	BUB1B	701	budding uninhibited by benzimidazoles 1 homolog beta (yeast)
	CDKN2A	1029	cyclin-dependent kinase inhibitor 2A (melanoma, p16, inhibits CDK4)
	CFTR	1080	cystic fibrosis transmembrane conductance regulator (ATP-binding cas
	KRAS	3845	v-Ki-ras2 Kirsten rat sarcoma viral oncogene homolog
	MARK2	2011	MAP/microtubule affinity-regulating kinase 2
	MOAP1	64112	modulator of apoptosis 1
	NEK2	4751	NIMA (never in mitosis gene a)-related kinase 2
	PIM2	11040	pim-2 oncogene
	PTK2	5747	PTK2 protein tyrosine kinase 2
	SEC23IP	11196	SEC23 interacting protein
	STK36	27148	serine/threonine kinase 36
	TNK2	10188	tyrosine kinase, non-receptor, 2
	TOP2A	7153	topoisomerase (DNA) II alpha 170kDa
	TP53	7157	tumor protein p53
	TP63	8626	tumor protein p63
	ZW10	9183	ZW10, kinetochore associated, homolog (Drosophila)
	ZWINT	11130	ZW10 interactor
	WRN	7486	Werner syndrome ATP-dependent helicase
	PMAIP1	5366	phorbol-12-myristate-13-acetate-induced protein 1
	PML	5371	promyelocytic leukemia
	TTK	7272	TTK protein kinase
DNA damage response	ATM	472	ataxia telangiectasia mutated
	CSNK1E	1454	casein kinase 1, epsilon
	FANCA	2175	Fanconi anemia, complementation group A
	MLL	4297	myeloid/lymphoid or mixed-lineage leukemia (trithorax homolog, Droso
	MTOR	2475	mechanistic target of rapamycin (serine/threonine kinase)
	NSMCE1	197370	non-SMC element 1 homolog (S. cerevisiae)
	SMG1	23049	SMG1 homolog, phosphatidylinositol 3-kinase-related kinase (C. elega
	TP73	7161	tumor protein p73
	TYMS	7298	thymidylate synthetase
	XPC	7508	xeroderma pigmentosum, complementation group C
	CHEK1	1111	CHK1 checkpoint homolog (S. pombe)
	CHEK2	11200	CHK2 checkpoint homolog (S. pombe)
	RAD17	5884	RAD17 homolog (S. pombe)
	APC	324	adenomatous polyposis coli
	POLH	5429	POLH polymerase (DNA directed), eta

DNA metabolism	DAPK3	1613	death-associated protein kinase 3
	ERCC2	2068	excision repair cross-complementing rodent repair deficiency, complementation group
	ERCC3	2071	excision repair cross-complementing rodent repair deficiency, complementation group
	HDAC1	3065	histone deacetylase 1
	HDAC10	83933	histone deacetylase 10
	HDAC11	79885	histone deacetylase 11
	HDAC2	3066	histone deacetylase 2
	HDAC3	8841	histone deacetylase 3
	HDAC4	9759	histone deacetylase 4
	HDAC5	10014	histone deacetylase 5
	HDAC6	10013	histone deacetylase 6
	HDAC8	55869	histone deacetylase 8
	HDAC9	9734	histone deacetylase 9
	MYST4	23522	MYST histone acetyltransferase (monocytic leukemia) 4
	NR3C1	2908	nuclear receptor subfamily 3, group C, member 1 (glucocorticoid receptor)
	RB1	5925	retinoblastoma 1
	RECQL4	9401	RecQ protein-like 4
	TOP1	7150	topoisomerase (DNA) I
	TOP2B	7155	topoisomerase (DNA) II beta 180kDa
	HDAC7	51564	histone deacetylase 7
	CAT	847	catalase
	TUBA4A	7277	tubulin, alpha 4a
	TUBB	203068	tubulin, beta
	UGT2B11	10720	UGT2B11 UDP glucuronosyltransferase 2 family, polypeptide B11
	ACHE	43	acetylcholinesterase
	TCF3	6929	transcription factor 3 (E2A immunoglobulin enhancer binding factors E12/E47)
Metabolism	ADK	132	adenosine kinase
	AKR1A1	10327	aldo-keto reductase family 1, member A1 (aldehyde reductase)
	ALDH1A1	216	aldehyde dehydrogenase 1 family, member A1
	ALOX12	239	arachidonate 12-lipoxygenase
	ALOX15	246	arachidonate 15-lipoxygenase
	ALOX5	240	arachidonate 5-lipoxygenase
	BHMT	635	betaine--homocysteine S-methyltransferase
	CA1	759	carbonic anhydrase I
	CHDH	55349	choline dehydrogenase
	COMT	1312	catechol-O-methyltransferase
	DHFR	1719	dihydrofolate reductase
	DPYS	1807	dihydropyrimidinase
	FDFT1	2222	farnesyl-diphosphate farnesyltransferase 1
	FDPS	2224	farnesyl diphosphate synthase
	FKBP1A	2280	FK506 binding protein 1A, 12kDa
	FKBP2	2286	FK506 binding protein 2, 13kDa
	FKBP3	2287	FK506 binding protein 3, 25kDa
	FKBP5	2289	FK506 binding protein 5
	FPGS	2356	folylpolyglutamate synthase
	GGH	8836	gamma-glutamyl hydrolase (conjugase, folylpolygammaglutamyl hydrolase)
	GLUL	2752	glutamate-ammonia ligase
	HMGCR	3156	3-hydroxy-3-methylglutaryl-CoA reductase
	LACTB	114294	lactamase, beta
	LIPF	8513	lipase, gastric
	LSS	4047	lanosterol synthase (2,3-oxidosqualene-lanosterol cyclase)
	MAOB	4129	monoamine oxidase B
	MMAB	326625	methylmalonic aciduria (cobalamin deficiency) cblB type
	MTHFS	10588	5,10-methenyltetrahydrofolate synthetase (5-formyltetrahydrofolate cyclo-ligase)
	NQO1	1728	NAD(P)H dehydrogenase, quinone 1
	PNPLA6	10908	patatin-like phospholipase domain containing 6
	PON1	5444	paraoxonase 1
	PPAP2B	8613	phosphatidic acid phosphatase type 2B
	PPIA	5478	peptidylprolyl isomerase A (cyclophilin A)
	S100B	6285	S100 calcium binding protein B

Metabolism	STS	412	steroid sulfatase (microsomal), isozyme S
	TH	7054	tyrosine hydroxylase
	TPMT	7172	thiopurine S-methyltransferase
	UGDH	7358	UDP-glucose 6-dehydrogenase
	UMPS	7372	uridine monophosphate synthetase
	XDH	7498	xanthine dehydrogenase
	ARSE	415	arylsulfatase E (chondrodysplasia punctata 1)
	GSTA1	2938	glutathione S-transferase alpha 1
	HPRT1	3251	hypoxanthine phosphoribosyltransferase 1
	NMNAT1	64802	nicotinamide nucleotide adenylyltransferase 1
	PTGS2	5743	prostaglandin-endoperoxide synthase 2 (prostaglandin G/H synthase a
	SQLE	6713	squalene epoxidase
	DCK	1633	deoxycytidine kinase
	TYR	7299	tyrosinase (oculocutaneous albinism IA)
	OAT	4942	ornithine aminotransferase
	ABP1	26	amiloride binding protein 1 (amine oxidase (copper-containing))
	CES2	8824	carboxylesterase 2 (intestine, liver)
	CHAT	1103	choline O-acetyltransferase
	CPA6	57094	CPA6 carboxypeptidase A6
	EBP	10682	EBP emopamil binding protein (sterol isomerase)
	FH	2271	fumarate hydratase
	FMO1	2326	flavin containing monooxygenase 1
	GRIN1	2902	GRIN1 glutamate receptor, ionotropic, N-methyl D-aspartate 1
	HDC	3067	histidine decarboxylase
	IMPDH1	3614	IMP (inosine 5'-monophosphate) dehydrogenase 1
	MME	4311	membrane metallo-endopeptidase
	NAT2	10	NAT2 N-acetyltransferase 2 (arylamine N-acetyltransferase)
	NR1H4	9971	nuclear receptor subfamily 1, group H, member 4
	PAM	5066	PAM peptidylglycine alpha-amidating monooxygenase
	REN	5972	renin
	SHBG	6462	sex hormone-binding globulin
	TPO	7173	TPO thyroid peroxidase
	PPP3CB	5532	protein phosphatase 3, catalytic subunit, beta isozyme
	PTPN1	5770	protein tyrosine phosphatase, non-receptor type 1
Protein amino acid phosphorylation	ALK	238	anaplastic lymphoma receptor tyrosine kinase
	AURKB	9212	aurora kinase B
	AURKC	6795	aurora kinase C
	CAMK1	8536	calcium/calmodulin-dependent protein kinase I
	CAMKK1	84254	calcium/calmodulin-dependent protein kinase kinase 1, alpha
	CCND1	595	cyclin D1
	CDK2	1017	cyclin-dependent kinase 2
	CDK4	1019	cyclin-dependent kinase 4
	CDK5	1020	cyclin-dependent kinase 5
	CDK6	1021	cyclin-dependent kinase 6
	DAPK2	23604	death-associated protein kinase 2
	DGUOK	1716	deoxyguanosine kinase
	F2	2147	coagulation factor II (thrombin)
	FER	2241	fer (fps/fes related) tyrosine kinase
	FGFR1	2260	fibroblast growth factor receptor 1
	FGR	2268	Gardner-Rasheed feline sarcoma viral (v-fgr) oncogene homolog
	FYN	2534	FYN oncogene related to SRC, FGR, YES
	GAK	2580	cyclin G associated kinase
	HCK	3055	hemopoietic cell kinase
	IKBKB	3551	inhibitor of kappa light polypeptide gene enhancer in B-cells, kinase be
	JAK1	3716	Janus kinase 1
	JAK2	3717	Janus kinase 2
	LYN	4067	v-yes-1 Yamaguchi sarcoma viral related oncogene homolog
	MKNK2	2872	MAP kinase interacting serine/threonine kinase 2
	MYLK2	85366	myosin light chain kinase 2
	NTRK1	4914	neurotrophic tyrosine kinase, receptor, type 1

Protein amino acid phosphorylation	PDGFRB	5159	platelet-derived growth factor receptor, beta polypeptide
	PIK3CA	5290	phosphoinositide-3-kinase, catalytic, alpha polypeptide
	PLK1	5347	polo-like kinase 1
	PLK4	10733	polo-like kinase 4
	PRKAA1	5562	protein kinase, AMP-activated, alpha 1 catalytic subunit
	RIPK2	8767	receptor-interacting serine-threonine kinase 2
	RPS6KA2	6196	ribosomal protein S6 kinase, 90kDa, polypeptide 2
	SRC	6714	v-src sarcoma (Schmidt-Ruppin A-2) viral oncogene homolog (avian)
	STK17B	9262	serine/threonine kinase 17b
	STK38L	23012	serine/threonine kinase 38 like
	YES1	7525	v-yes-1 Yamaguchi sarcoma viral oncogene homolog 1
	ERBB2	2064	Receptor tyrosine-protein kinase erbB-2
	ERBB3	2065	Receptor tyrosine-protein kinase erbB-3
	ERBB4	2066	Receptor tyrosine-protein kinase erbB-4
	IGF1R	3480	Insulin-like growth factor 1 receptor
	BRAF	673	v-raf murine sarcoma viral oncogene homolog B1
	MAP2K1	5604	mitogen-activated protein kinase kinase 1
	MAP2K4	6416	mitogen-activated protein kinase kinase 4
	MAPK8	5599	mitogen-activated protein kinase 8
	MAPK9	5601	mitogen-activated protein kinase 9
	EPHA2	1969	EPH receptor A2
	LCK	3932	lymphocyte-specific protein tyrosine kinase
	STK10	6793	serine/threonine kinase 10
	STK16	8576	serine/threonine kinase 16
	STK4	6789	serine/threonine kinase 4
	BCR	613	breakpoint cluster region
	ATR	545	ataxia telangiectasia and Rad3 related
	BMP2K	55589	BMP2 inducible kinase
	BMPR1A	657	bone morphogenetic protein receptor, type IA
	CLK1	1195	CDC-like kinase 1
	FRK	2444	fyn-related kinase
	PRKCA	5578	protein kinase C, alpha
	SLK	9748	SLK STE20-like kinase
Protein degradation	ADAM17	6868	ADAM metallopeptidase domain 17
	CAPN1	823	calpain 1, (mu/I) large subunit
	CHFR	55743	checkpoint with forkhead and ring finger domains
	CTSG	1511	cathepsin G
	DPP4	1803	dipeptidyl-peptidase 4
	LTA4H	4048	leukotriene A4 hydrolase
	MGEA5	10724	meningioma expressed antigen 5 (hyaluronidase)
	PGA4	643847	pepsinogen 4, group I (pepsinogen A)
	PLG	5340	plasminogen
	PRCP	5547	prolylcarboxypeptidase (angiotensinase C)
	PRSS1	5644	protease, serine, 1 (trypsin 1)
	PYCARD	29108	PYD and CARD domain containing
	VKORC1	79001	vitamin K epoxide reductase complex, subunit 1
	HSPCA	3320	heat shock protein 90kDa alpha (cytosolic), class A member 2
Protein transport	SLC15A1	6564	solute carrier family 15 (oligopeptide transporter), member 1
	ARHH	399	ras homolog gene family, member H
	RASD1	51655	RAS, dexamethasone-induced 1
	TSPO	706	translocator protein (18kDa)
	CD24	1E+08	Signal transducer CD24
	DICER1	23405	dicer 1, ribonuclease type III
	FIP1L1	81608	FIP1 like 1 (S. cerevisiae)

Signal transduction	ADORA1	134	adenosine A1 receptor
	ADORA2A	135	adenosine A2a receptor
	ADRA1B	147	adrenergic, alpha-1B-, receptor
	ADRB1	153	adrenergic, beta-1-, receptor
	AGTR1	185	angiotensin II receptor, type 1
	AGTR2	186	angiotensin II receptor, type 2
	AVPR1A	552	arginine vasopressin receptor 1A
	BDKRB2	624	bradykinin receptor B2
	CCND3	896	cyclin D3
	CRABP1	1381	cellular retinoic acid binding protein 1
	CREBBP	1387	CREB binding protein
	CXCR4	7852	chemokine (C-X-C motif) receptor 4
	CYSLTR1	10800	cysteinyl leukotriene receptor 1
	DRD4	1815	dopamine receptor D4
	EDNRA	1909	endothelin receptor type A
	GNRHR	2798	gonadotropin-releasing hormone receptor
	GPBAR1	151306	G protein-coupled bile acid receptor 1
	GPR109A	338442	G protein-coupled receptor 109A
	GRB2	2885	growth factor receptor-bound protein 2
	ITGA2B	3674	integrin, alpha 2b (platelet glycoprotein IIb of IIb/IIIa complex, antigen C
	LTB4R	1241	leukotriene B4 receptor
	MLNR	2862	motilin receptor
	MTNR1A	4543	melatonin receptor 1A
	NCOA2	10499	nuclear receptor coactivator 2
	NISCH	11188	nischarin
	NR1I2	8856	nuclear receptor subfamily 1, group I, member 2
	OXTR	5021	oxytocin receptor
	P2RX1	5023	purinergic receptor P2X, ligand-gated ion channel, 1
	P2RY1	5028	purinergic receptor P2Y, G-protein coupled, 1
	P2RY4	5030	pyrimidinergic receptor P2Y, G-protein coupled, 4
	PDE11A	50940	phosphodiesterase 11A
	PDE1A	5136	phosphodiesterase 1A, calmodulin-dependent
	PDE3A	5139	phosphodiesterase 3A, cGMP-inhibited
	PPARG	5468	peroxisome proliferator-activated receptor gamma
	RARA	5914	retinoic acid receptor, alpha
	RARB	5915	retinoic acid receptor, beta
	RXRA	6256	retinoid X receptor, alpha
	RYR1	6261	ryanodine receptor 1 (skeletal)
	SSTR1	6751	somatostatin receptor 1
	TACR1	6869	tachykinin receptor 1
	TBXA2R	6915	thromboxane A2 receptor
	TLR3	7098	toll-like receptor 3
	TLR7	51284	toll-like receptor 7
	MCL1	4170	myeloid cell leukemia sequence 1 (BCL2-related)
	ARHGEF12	23365	Rho guanine nucleotide exchange factor (GEF) 12
	CXCR2	3579	chemokine (C-X-C motif) receptor 2
	IL2RA	3559	interleukin 2 receptor, alpha
	IL2RB	3560	interleukin 2 receptor, beta
	IL2RG	3561	interleukin 2 receptor, gamma
	LOX	4015	lysyl oxidase
	NR1I3	9970	nuclear receptor subfamily 1, group I, member 3
	OPRL1	4987	opiate receptor-like 1
	PGR	5241	progesterone receptor
	PTAFR	5724	platelet-activating factor receptor
	PTGER1	5731	prostaglandin E receptor 1 (subtype EP1), 42kDa
	TRHR	7201	thyrotropin-releasing hormone receptor
	CXCR1	3577	chemokine (C-X-C motif) receptor 1
	GPR44	11251	G protein-coupled receptor 44
	HRH1	3269	HRH1 histamine receptor H1
	HRH2	3274	HRH2 histamine receptor H2
	CNR1	1268	cannabinoid receptor 1 (brain)
	DBI	1622	DBI diazepam binding inhibitor (GABA receptor modulator, acyl-CoA bi
	EGFR	1956	epidermal growth factor receptor
	FPR1	2357	formyl peptide receptor 1
	ITGB3	3690	integrin, beta 3 (platelet glycoprotein IIIa, antigen CD61)
	NPC1L1	29881	NPC1 (Niemann-Pick disease, type C1, gene)-like 1
	VDR	7421	vitamin D (1,25- dihydroxyvitamin D3) receptor
	INSR	3643	Insulin receptor

Stress response	GSR	2936	glutathione reductase
	HSPA4	3308	heat shock 70kDa protein 4
	HSPA8	3312	heat shock 70kDa protein 8
	PPARA	5465	peroxisome proliferator-activated receptor alpha
Transcription	ETV6	2120	ets variant 6
	HUWE1	10075	HECT, UBA and WWE domain containing 1
	PPARD	5467	peroxisome proliferator-activated receptor delta
	RXRB	6257	retinoid X receptor, beta
	WT1	7490	Wilms tumor 1
Transport	CYP2A6	1548	cytochrome P450, family 2, subfamily A, polypeptide 6
	CYP2C9	1559	cytochrome P450, family 2, subfamily C, polypeptide 9
	CYP3A4	1576	cytochrome P450, family 3, subfamily A, polypeptide 4
	SLC12A2	6558	solute carrier family 12 (sodium/potassium/chloride transporters), mem
	SLC18A1	6570	solute carrier family 18 (vesicular monoamine), member 1
	SLC19A1	6573	solute carrier family 19 (folate transporter), member 1
	SLC22A6	9356	solute carrier family 22 (organic anion transporter), member 6
	SLC29A1	2030	solute carrier family 29 (nucleoside transporters), member 1
	SLC35A2	7355	solute carrier family 35 (UDP-galactose transporter), member A2
	SLCO1A2	6579	solute carrier organic anion transporter family, member 1A2
	SLCO1B1	10599	solute carrier organic anion transporter family, member 1B1
	SLCO1B3	28234	solute carrier organic anion transporter family, member 1B3
	SLCO4A1	28231	solute carrier organic anion transporter family, member 4A1
	SLCO4C1	353189	solute carrier organic anion transporter family, member 4C1
	ABCC3	8714	ATP-binding cassette, sub-family C (CFTR/MRP), member 3
	ABCG2	9429	ATP-binding cassette, sub-family G (WHITE), member 2
	ACCN1	40	amiloride-sensitive cation channel 1, neuronal
	AKR1C1	1645	aldo-keto reductase family 1, member C1 (dihydrodiol dehydrogenase
	AMD1	262	adenosylmethionine decarboxylase 1
	CACNA1G	8913	calcium channel, voltage-dependent, T type, alpha 1G subunit
	CACNA1S	779	calcium channel, voltage-dependent, L type, alpha 1S subunit
	CACNA2D1	781	calcium channel, voltage-dependent, alpha 2/delta subunit 1
	CNGA1	1259	cyclic nucleotide gated channel alpha 1
	CPT2	1376	carnitine palmitoyltransferase 2
	FADS2	9415	fatty acid desaturase 2
	FKBP4	2288	FK506 binding protein 4, 59kDa
	GC	2638	group-specific component (vitamin D binding protein)
	KCNA5	3741	potassium voltage-gated channel, shaker-related subfamily, member 5
	KCND3	3752	potassium voltage-gated channel, Shal-related subfamily, member 3
	PCTP	58488	phosphatidylcholine transfer protein
	SCN2A	6326	sodium channel, voltage-gated, type II, alpha subunit
	SIGMAR1	10280	sigma non-opioid intracellular receptor 1
	TCN2	6948	transcobalamin II
	TRPV3	162514	transient receptor potential cation channel, subfamily V, member 3
	ABCB1	5243	ATP-binding cassette, sub-family B (MDR/TAP), member 1
	ALO17	57674	RNF213 ring finger protein 213
Unknown	BCL2L10	10017	BCL2-like 10 (apoptosis facilitator)
	BCL2L11	10018	BCL2-like 11 (apoptosis facilitator)
	BCL2L13	23786	BCL2-like 13 (apoptosis facilitator)
	BCL2L14	79370	BCL2-like 14 (apoptosis facilitator)
	BCL2L2	599	BCL2-like 2
	BCL2L4	581	BCL2-associated X protein
	BCL2L9	666	BCL2-related ovarian killer
	CDK16	5127	cyclin-dependent kinase 16
	CRTAM	56253	cytotoxic and regulatory T cell molecule
	ELANE	1991	elastase, neutrophil expressed
	EPYC	1833	epiphycan
	HIST1H4I	8294	histone cluster 1, H4i
	KSP	3832	KIF11 kinesin family member 11
	MAD2L1BP	9587	MAD2L1 binding protein
	PEBP1	5037	phosphatidylethanolamine binding protein 1
	S100A13	6284	S100 calcium binding protein A13
	SFTPC	6440	surfactant protein C
	ZWILCH	55055	Zwilch, kinetochore associated, homolog (Drosophila)
	CD44	960	CD44 antigen
	CD133	8842	Prominin-1
	OGFR	11054	opioid growth factor receptor
	ADCY10	55811	adenylate cyclase 10 (soluble)

Figure S1 List of human gene targets in the minipool grouped by gene ontology (gray box).
