# Supplementary material for: Miniature Short Hairpin RNA Screens to Characterize Antiproliferative Drugs
Source: G3 (Bethesda). 2013 Aug 1;3(8):1375–87. doi: 10.1534/g3.113.006437 (PMC3737177; doi:10.1534/g3.113.006437)
Supplement: Supporting Information [file supp_g3.113.006437_FigureS2.pdf]

A.

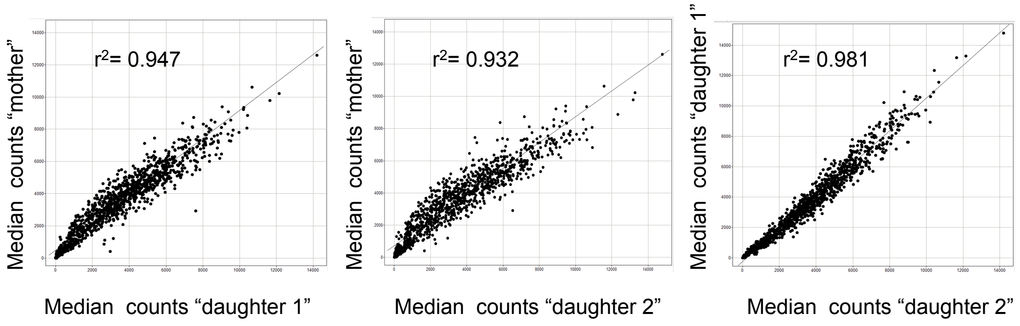

B.

	Mother	Daughter 1	Daughter 2
Hairpins missing	19.24 ± 1.70	24.5 ± 0.65	25.5 ± 1.04

Figure S2 Hairpin representation in the A549 cells used for the synthetic lethality drug screen. (A) Correlation of hairpin counts between freshly transduced A549s ("mother") and two derived independent cell stocks ("daughter 1" and "daughter 2"). Median counts were calculated from 4 technical replicates. Raw count ranged from 0 to 17,000. (B) Hairpins present in freshly transduced A549s and two derived independent cell stocks. After normalization, hairpins with a count number below 50 were considered missing from the minipool population. Median counts were calculated from 4 technical replicates.
