# Supplementary material for: Miniature Short Hairpin RNA Screens to Characterize Antiproliferative Drugs
Source: G3 (Bethesda). 2013 Aug 1;3(8):1375–87. doi: 10.1534/g3.113.006437 (PMC3737177; doi:10.1534/g3.113.006437)
Supplement: Supporting Information [file supp_g3.113.006437_FigureS3.pdf]

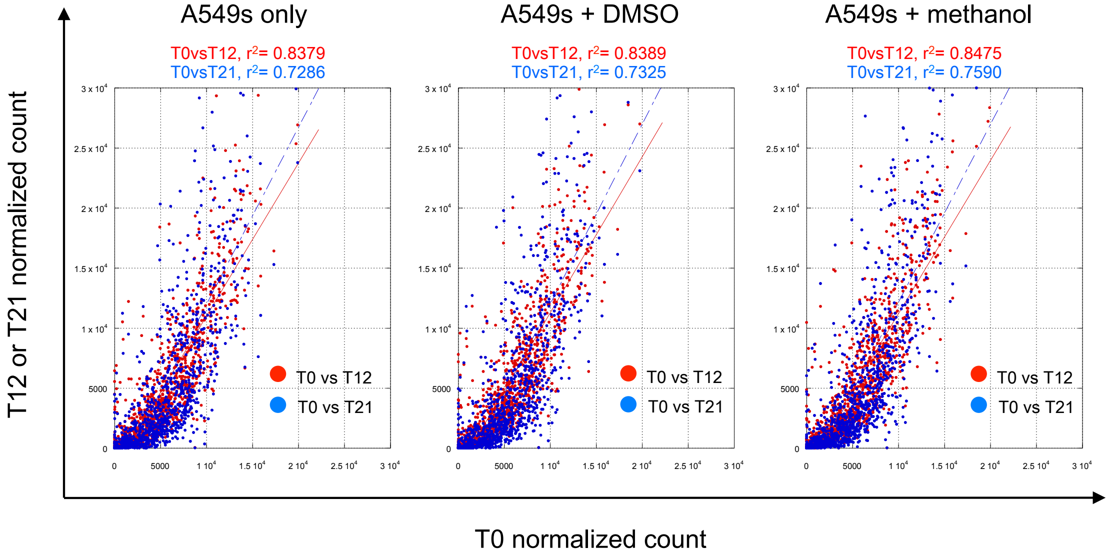

Figure S3 Hairpin representations in the A549 cells used for the synthetic lethality drug screen. Scatter plots comparing the hairpin representations at T0 vs T12 (red dots) and T0 vs. T21 (blue dots) of the A549s cultured in the absence of control vehicle (left panel), in the presence of DMSO (middle panel) or in the presence of methanol (right panel). Hairpin count was based on the results of experiments done in triplicate.
