# Supplementary material for: Miniature Short Hairpin RNA Screens to Characterize Antiproliferative Drugs
Source: G3 (Bethesda). 2013 Aug 1;3(8):1375–87. doi: 10.1534/g3.113.006437 (PMC3737177; doi:10.1534/g3.113.006437)
Supplement: Supporting Information [file supp_g3.113.006437_FigureS4.pdf]

Cells only			
T0 vs T12		T0 vs T21	
hpid	*gene*	*hpid*	*gene*
TRCN0000045929	FPGS	TRCN0000045929	FPGS
TRCN0000004828	HDAC3	TRCN0000056848	TLR3
TRCN0000006988	STK36	TRCN0000006988	STK36
TRCN0000117932	KNTC1	TRCN0000117932	KNTC1
TRCN0000044140	CNGA1	TRCN0000044140	CNGA1
TRCN0000195066	BRAF	TRCN0000195066	BRAF
TRCN0000000861	PRKAA1	TRCN0000000861	PRKAA1
TRCN0000121193	IGF1R	TRCN0000121193	IGF1R
TRCN0000195487	FRK	TRCN0000195487	FRK
TRCN0000053951	S100A13	TRCN0000047354	RASD1
TRCN0000002814	PPP3CB	TRCN0000002814	PPP3CB
TRCN0000001625	STK4	TRCN0000001625	STK4
TRCN0000196538	ATR	TRCN0000196538	ATR
TRCN0000078448	HDC	TRCN0000058753	IL2RB
TRCN0000009065	HRH1	TRCN0000221544	MTOR
TRCN0000222166	MYST4	TRCN0000003671	CTSG
TRCN0000074361	TUBB	TRCN0000003091	CPT2
TRCN0000121067	EGFR	TRCN0000078448	HDC
TRCN0000051342	GGH		HRH1
TRCN0000155335	ZW10	TRCN0000056619	TH
TRCN0000021604	NR1H4	TRCN0000121067	EGFR
TRCN0000000501	CHEK1	TRCN0000044138	CNGA1
TRCN0000056848	TLR3	TRCN0000039634	EGFR
TRCN0000060003	TCN2	TRCN0000051342	GGH
TRCN0000047354	RASD1	TRCN0000155335	ZW10
TRCN0000152366	B3GAT1	TRCN0000199796	CHEK1
TRCN0000160294	NPC1L1	TRCN0000199993	ERBB3
TRCN0000003671	CTSG	TRCN0000003755	TP53
TRCN0000036544	AKR1C1	TRCN0000074359	TUBB
TRCN0000046744	DPYS	TRCN0000008816	GNRHR
		TRCN0000014135	PTGER1
**Total**	**30**	TRCN0000083188	SFTPC
		TRCN0000038972	VKORC1
		TRCN0000014134	PTGER1
		TRCN0000046419	AKR1A1
		TRCN0000014451	LTB4R
		TRCN0000009271	OPRL1
		TRCN0000018917	IKBKB
		TRCN0000003753	TP53
		TRCN0000033500	BCL2L1
		TRCN0000152366	B3GAT1
		TRCN0000057762	ITGA2B
		TRCN0000046856	ACHE
		TRCN0000009971	HCK
		TRCN0000039747	CDK6
		TRCN0000051244	ARSE
		TRCN0000004842	HDAC6
		TRCN0000005956	MLL
		TRCN0000083190	SFTPC
		TRCN0000199622	INSR
		TRCN0000034909	NAT2
		TRCN0000004905	BLM
		TRCN0000008092	AGTR1
		TRCN0000222166	MYST4
		TRCN0000166735	MOAP1
		TRCN0000161240	NISCH
		TRCN0000018331	IGF1R
		TRCN0000150938	ZW10
		TRCN0000199601	CAMKK1
		TRCN0000001994	NTRK1
		TRCN0000004828	HDAC3
		TRCN0000197158	HDAC11
		TRCN0000056569	ALOX5
		TRCN0000199323	MTOR
		TRCN0000003234	ITGB3
		TRCN0000010369	KRAS
		TRCN0000009459	OXTR
		**Total**	**67**

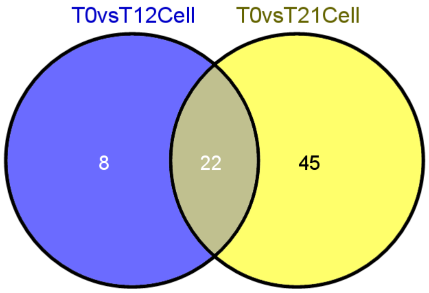

Cells + DMSO			
T0 vs T12		T0 vs T21	
hpid	*gene*	*hpid*	*gene*
TRCN0000045929	FPGS	TRCN0000045929	FPGS
TRCN0000004828	HDAC3	TRCN0000004828	HDAC3
TRCN0000056848	TLR3	TRCN0000056848	TLR3
TRCN0000117932	KNTC1	TRCN0000117932	KNTC1
TRCN0000044140	CNGA1	TRCN0000006988	STK36
TRCN0000195066	BRAF	TRCN0000044140	CNGA1
TRCN0000000861	PRKAA1	TRCN0000000861	PRKAA1
TRCN0000197098	CDK4	TRCN0000197098	CDK4
TRCN0000121193	IGF1R	TRCN0000121193	IGF1R
TRCN0000060003	TCN2	TRCN0000060003	TCN2
TRCN0000083189	SFTPC	TRCN0000195487	FRK
TRCN0000195487	FRK	TRCN0000057762	ITGA2B
TRCN0000047354	RASD1	TRCN0000001028	HSP90AA1
TRCN0000002814	PPP3CB	TRCN0000047354	RASD1
TRCN0000036544	AKR1C1	TRCN0000053951	S100A13
TRCN0000196538	ATR	TRCN0000051244	ARSE
TRCN0000058753	IL2RB	TRCN0000002814	PPP3CB
TRCN0000221544	MTOR	TRCN0000001625	STK4
TRCN0000003671	CTSG	TRCN0000196538	ATR
TRCN0000078448	HDC	TRCN0000058753	IL2RB
TRCN0000046419		TRCN0000221544	MTOR
TRCN0000044138	CNGA1	TRCN0000003671	CTSG
TRCN0000159815	NPC1L1	TRCN0000009065	HRH1
TRCN0000051342	GGH	TRCN0000074361	TUBB
TRCN0000004905	BLM	TRCN0000121067	EGFR
TRCN0000196844	BRAF	TRCN0000046419	AKR1A1
TRCN0000039916	MAP2K4	TRCN0000044138	CNGA1
TRCN0000083188	SFTPC	TRCN0000159815	NPC1L1
TRCN0000053951	S100A13	TRCN0000051342	GGH
TRCN0000034818	UMPS	TRCN0000155335	ZW10
TRCN0000001028	HSP90AA1	TRCN0000036243	COMT
TRCN0000152366	B3GAT1	TRCN0000001994	NTRK1
TRCN0000045664	TYMS	TRCN0000043299	SLC15A1
TRCN0000199622	INSR	TRCN0000221543	MTOR
TRCN0000008092	AGTR1	TRCN0000063370	GRIN1
TRCN0000050453	PPAP2B	TRCN0000018331	IGF1R
TRCN0000064757	FADS2	TRCN0000039748	CDKN2A
TRCN0000014134	PTGER1	TRCN0000033500	BCL2L1
TRCN0000009271	OPRL1	TRCN0000005956	MLL
		TRCN0000014134	PTGER1
**Total**	**39**	TRCN0000046744	DPYS
		TRCN0000036324	FDFT1
		TRCN0000003755	TP53
		TRCN0000034818	UMPS
		TRCN0000003234	ITGB3
		TRCN0000083188	SFTPC
		TRCN0000195066	BRAF
		TRCN0000009271	OPRL1
		TRCN0000199323	MTOR
		TRCN0000039003	DHFR
		TRCN0000000916	BMP2K
		TRCN0000161240	NISCH
		TRCN0000039916	MAP2K4
		TRCN0000199622	INSR
		TRCN0000166735	MOAP1
		TRCN0000219684	MAP2K1
		TRCN0000014135	PTGER1
		TRCN0000121325	PLK1
		TRCN0000009971	HCK
		TRCN0000038956	DCXR
		TRCN0000014451	LTB4R
		TRCN0000045664	TYMS
		TRCN0000003753	TP53
		TRCN0000018917	IKBKB
		TRCN0000008899	MLNR
		TRCN0000152366	B3GAT1
		TRCN0000056569	ALOX5
		TRCN0000036145	BHMT
		TRCN0000021617	RXRA
		TRCN0000040093	APC
		TRCN0000219689	FRK
		TRCN0000150938	ZW10
		TRCN0000004842	HDAC6
		**Total**	**73**

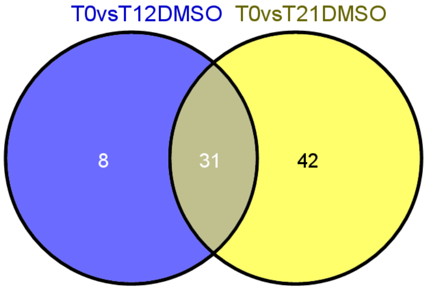

Cells + methanol			
T0 vs T12		T0 vs T21	
hpid	*gene*	*hpid*	*gene*
TRCN0000045929	FPGS	TRCN0000056848	TLR3
TRCN0000117932	KNTC1	TRCN0000117932	KNTC1
TRCN0000195066	BRAF	TRCN0000006988	STK36
TRCN0000000861	PRKAA1	TRCN0000044140	CNGA1
TRCN0000083189	SFTPC	TRCN0000000861	PRKAA1
TRCN0000195487	FRK	TRCN0000121193	IGF1R
TRCN0000001028	HSP90AA1	TRCN0000083189	SFTPC
TRCN0000047354	RASD1	TRCN0000195487	FRK
TRCN0000002814	PPP3CB	TRCN0000001028	HSP90AA1
TRCN0000001625	STK4	TRCN0000002814	PPP3CB
TRCN0000058753	IL2RB	TRCN0000058753	IL2RB
TRCN0000003671	CTSG	TRCN0000221544	MTOR
TRCN0000078448	HDC	TRCN0000003671	CTSG
TRCN0000044138	CNGA1	TRCN0000078448	HDC
TRCN0000003100	FYN	TRCN0000121067	EGFR
TRCN0000083188	SFTPC	TRCN0000046419	AKR1A1
TRCN0000199796	CHEK1	TRCN0000044138	CNGA1
TRCN0000001994	NTRK1	TRCN0000051342	GGH
TRCN0000056848	TLR3	TRCN0000197222	CDK16
TRCN0000155335	ZW10	TRCN0000003755	TP53
TRCN0000152366	B3GAT1	TRCN0000034818	UMPS
TRCN0000121193	IGF1R	TRCN0000073304	HUWE1
TRCN0000006076	DGUOK	TRCN0000083188	SFTPC
TRCN0000221544	MTOR	TRCN0000195066	BRAF
TRCN0000006988	STK36	TRCN0000197236	CDK2
TRCN0000045664	TYMS	TRCN0000003091	CPT2
TRCN0000001025	HSP90AA1	TRCN0000033500	BCL2L1
TRCN0000060003	TCN2	TRCN0000045664	TYMS
TRCN0000121067	EGFR	TRCN0000166735	MOAP1
		TRCN0000001593	FGR
**Total**	**29**	TRCN0000003991	TOP1
		TRCN0000197098	CDK4
		TRCN0000004905	BLM
		TRCN0000152366	B3GAT1
		TRCN0000050453	PPAP2B
		TRCN0000005956	MLL
		TRCN0000057762	ITGA2B
		TRCN0000199323	MTOR
		TRCN0000074361	TUBB
		TRCN0000121325	PLK1
		TRCN0000009971	HCK
		TRCN0000034909	NAT2
		TRCN0000221543	MTOR
		TRCN0000010369	KRAS
		TRCN0000046744	DPYS
		TRCN0000047354	RASD1
		TRCN0000199993	ERBB3
		TRCN0000051244	ARSE
		TRCN0000014134	PTGER1
		TRCN0000155335	ZW10
		TRCN0000038956	DCXR
		TRCN0000039634	EGFR
		**Total**	**52**

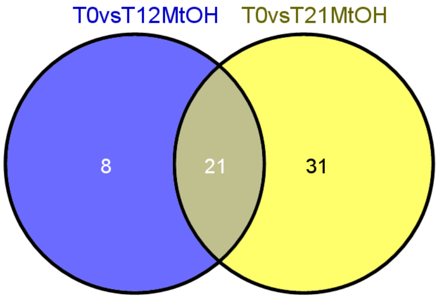

Figure S4 List of toxic hairpins. Based on the scatter plots represented in Figure S3, a list of toxic hairpins (i.e. hairpins that drop out of the minipool overtime) was established. Venn diagrams show the overlap between hairpins missing when comparing T0vsT12 and T0vsT21, for the same culture conditions.
