# Supplementary material for: Miniature Short Hairpin RNA Screens to Characterize Antiproliferative Drugs
Source: G3 (Bethesda). 2013 Aug 1;3(8):1375–87. doi: 10.1534/g3.113.006437 (PMC3737177; doi:10.1534/g3.113.006437)
Supplement: Supporting Information [file supp_g3.113.006437_FigureS5.pdf]

A.

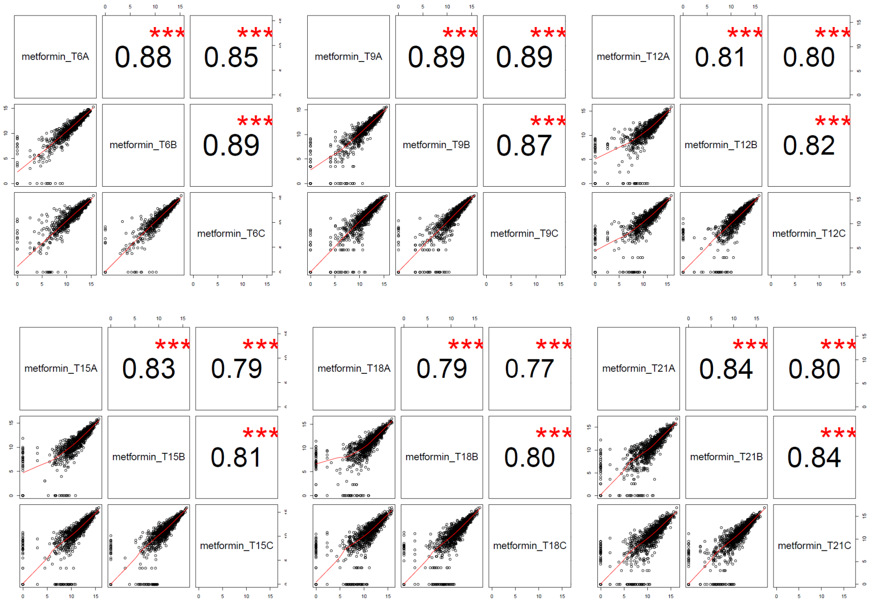

B.

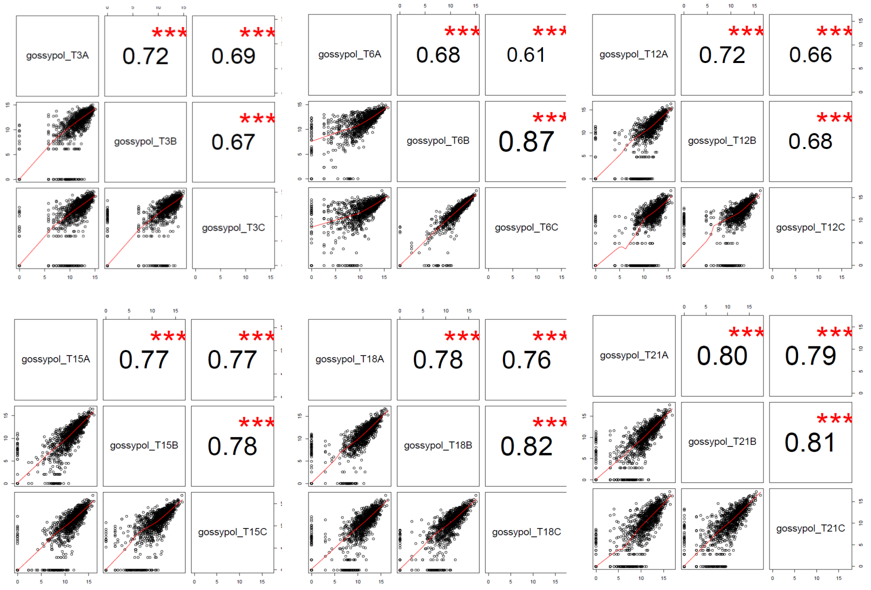

Figure S5 Correlation plots between triplicate (A, B, and C) of metformin (A.) and gossypol (B.) screen. Normalized Log2 of sequencing counts of each time point (T) has been stated. For each drug, the triplicate screens showed a high correlation (e.g. $r^2 \geq 0.8$, day 21) demonstrating a consistent hairpin drop out among triplicates and over time.
