# Supplementary material for: Miniature Short Hairpin RNA Screens to Characterize Antiproliferative Drugs
Source: G3 (Bethesda). 2013 Aug 1;3(8):1375–87. doi: 10.1534/g3.113.006437 (PMC3737177; doi:10.1534/g3.113.006437)
Supplement: Supporting Information [file supp_g3.113.006437_FigureS6.pdf]

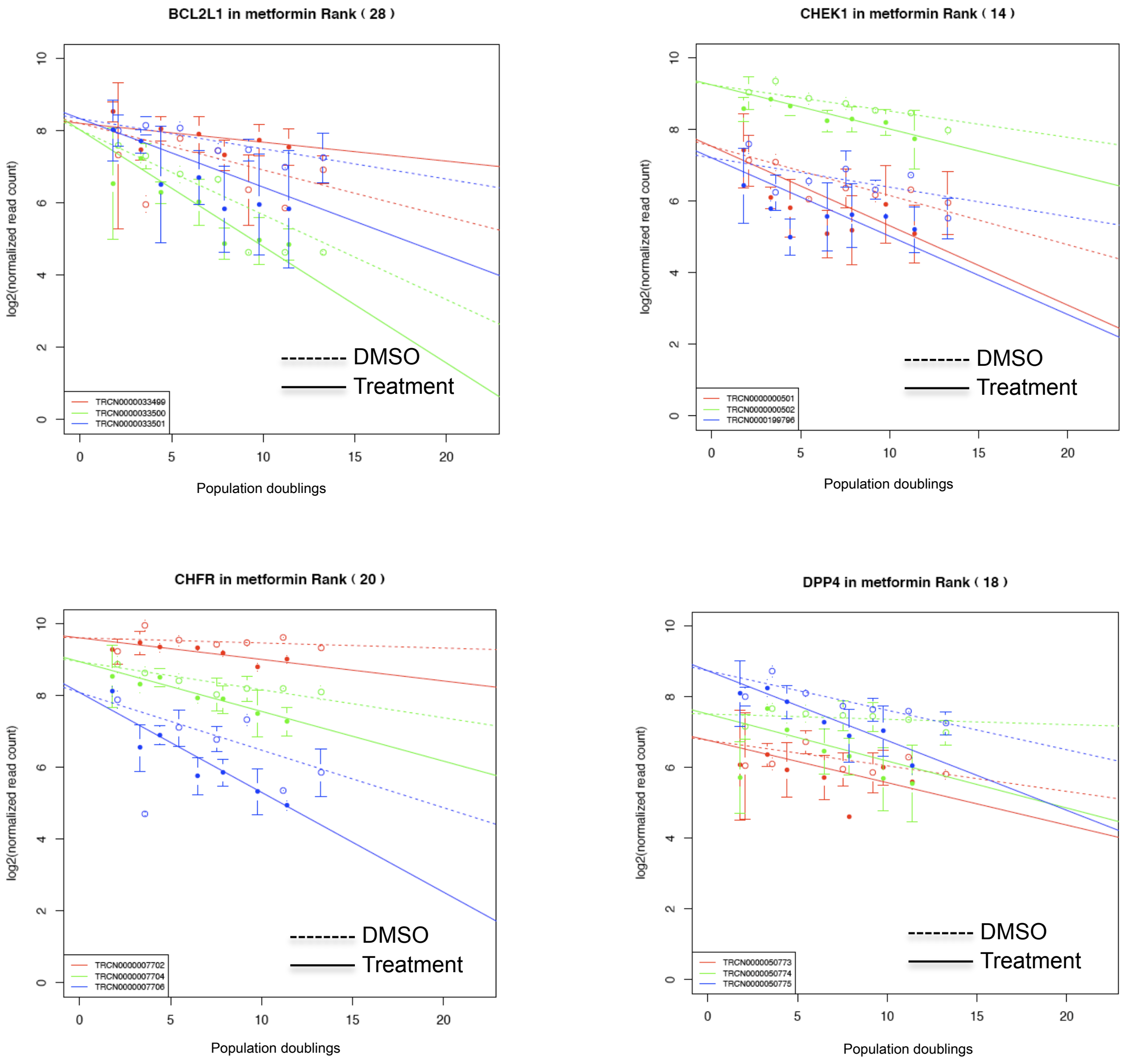

Figure S6 Linear plots of an individual gene that was knocking down by shRNA in A549 cells cultured in the presence of metformin are BCL2L1, CHEK1, CHFR and DPP4 respectively. Y-axis exhibits the log2 of normalization sequencing counts and X-axis demonstrates the doubling time in the screen. The combination of shRNAs and metformin treatment shows the decreasing in the signal read (solid line) compared to vehicle control (dash line).
