# Supplementary material for: Miniature Short Hairpin RNA Screens to Characterize Antiproliferative Drugs
Source: G3 (Bethesda). 2013 Aug 1;3(8):1375–87. doi: 10.1534/g3.113.006437 (PMC3737177; doi:10.1534/g3.113.006437)
Supplement: Supporting Information [file supp_g3.113.006437_FigureS7.pdf]

	Q1: FL1-H–, FL2-H+	Q2: FL1-H+, FL2-H+	Q3: FL1-H+, FL2-H–	Q4: FL1-H–, FL2-H–
A549_cellonly	0.397	0	0	99.6
A549_annexinV	0.705	2.68	1.41	95.2
A549_PI	3.74	0	0	96.3
A549_NoDrug	3.5	0.937	0.134	95.4
A549+5uM_gossypol	10.4	2.28	0.178	87.2
RFP	4.7	5.01	1.29	89
RFP+5uM_gossypol	12	5.6	0.298	82.1
KCND3	9.11	7.59	0.623	82.7
KCND3+5uM_gossypol	22.9	17.1	0.299	59.7
DBI_hp1	11.1	7.14	0.848	80.9
DBI_hp1+5uM_gossypol	19.7	5.71	0.327	74.2
**DBI_hp2**	**16.6**	**8.58**	**0.644**	**74.2**
**DBI_hp2+5uM_gossypol**	**27.5**	**40.4**	**0.224**	**31.8**
**CRTAM**	**9.97**	**8.13**	**0.657**	**81.2**
**CRTAM+5uM_gossypol**	**13.2**	**30.4**	**0.454**	**55.9**
CHAT_hp1	4.63	4.38	0.67	90.3
CHAT_hp1+5uM_gossypol	12.5	10.7	0.617	76.1
**CHAT_hp2**	**8.84**	**9.67**	**1.24**	**80.3**
**CHAT_hp2+5uM_gossypol**	**20.7**	**21**	**0.453**	**57.8**
**ErbB2_hp1**	**10.6**	**6.02**	**0.385**	**83**
**ErbB2_hp1+5uM_gossypol**	**18.7**	**16.7**	**0.309**	**64.3**
**ErbB2_hp2**	**13.3**	**12**	**1.01**	**73.7**
**ErbB2_hp2+5uM_gossypol**	**27.1**	**26.6**	**0.415**	**45.9**
**PTK2_hp1**	**10.9**	**4.37**	**0.283**	**84.4**
**PTK2_hp1+5uM_gossypol**	**18.9**	**15.9**	**0.221**	**65.1**
**PTK2_hp2**	**9.12**	**4.57**	**0.114**	**86.2**
**PTK2_hp2+5uM_gossypol**	**15.8**	**19.8**	**0.824**	**63.7**
**PTK2_hp3**	**11.3**	**16.1**	**1.65**	**70.9**
**PTK2_hp3+5uM_gossypol**	**23.2**	**27**	**0.712**	**49**
**BRCA2_hp1**	**9.1**	**8.5**	**0.447**	**82**
**BRCA2_hp1+5uM_gossypol**	**13.1**	**28.7**	**0.876**	**57.3**
**BRCA2_hp2**	**7.58**	**4.56**	**0.383**	**87.5**
**BRCA2_hp2+5uM_gossypol**	**12.5**	**17.1**	**0.918**	**69.5**

	Q1: FL1-H–, FL2-H+	Q2: FL1-H+, FL2-H+	Q3: FL1-H+, FL2-H–	Q4: FL1-H–, FL2-H–
A549cell only	0.183	0.0203	0	99.8
A549_PI	2.66	0	0	97.3
A549_AnnexinV	0.258	2.68	1.15	95.9
A549_NoDrug	8.45	3.84	0.736	87
A549+5uM_gossypol	21.9	3.97	0.119	74
LacZ_NoDrug	13.6	2.91	0.299	83.2
LacZ+5uM_gossypol	14.3	5.62	0.913	79.1
Luciferase_NoDrug	12.7	10.3	1.71	75.3
Luciferase+5uM_gossypol	27.3	8.2	0.413	64.1
RFP_NoDrug	12.7	12.1	2.19	73
RFP+5uM_gossypol	17.8	10.2	1.22	70.8
**CHEK1**	**9.54**	**9.39**	**0.835**	**80.2**
**CHEK1+5uM_gossypol**	**13.8**	**29.4**	**0.881**	**55.9**
MCL1	18.7	19.9	2.21	59.2
MCL1+5uM_gossypol	20.4	19.1	1.94	58.6
**HSPA8_hp1**	**11.1**	**19.4**	**3.95**	**65.6**
**HSPA8_hp1+5uM_gossypol**	**22.8**	**10.8**	**0.771**	**65.6**
**HSPA8_hp2**	**11**	**7.85**	**0.972**	**80.2**
**HSPA8_hp2+5uM_gossypol**	**16.3**	**23.8**	**0.841**	**59.1**
CHFR_hp1	11.5	7.45	1.36	79.7
CHFR_hp1+5uM_gossypol	18.9	13.2	0.674	67.1
CHFR_hp2	16	10.5	0.811	72.7
CHFR_hp2+5uM_gossypol	29.2	21.2	0.432	49.1
**CHFR_hp3**	**17.1**	**7.15**	**0.435**	**75.3**
**CHFR_hp3+5uM_gossypol**	**23.5**	**23.7**	**0.495**	**52.3**
**HUWE1_hp1**	**8.1**	**9.37**	**1.05**	**81.5**
**HUWE1_hp1+5uM_gossypol**	**12.6**	**19.8**	**0.567**	**67**
**HUWE1_hp2**	**11.5**	**7.95**	**0.887**	**79.7**
**HUWE1_hp2+5uM_gossypol**	**12.2**	**22.7**	**0.877**	**64.2**
**HUWE1_hp3**	**14.2**	**21.9**	**2.74**	**61.2**
**HUWE1_hp3+5uM_gossypol**	**24.1**	**36.6**	**0.416**	**38.9**
**HDAC2_hp1**	**18.1**	**27.5**	**2.44**	**51.9**
**HDAC2_hp1+5uM_gossypol**	**20.6**	**40**	**1.11**	**38.3**
**HDAC2_hp2**	**10.6**	**9.6**	**0.583**	**79.3**
**HDAC2_hp2+5uM_gossypol**	**19.3**	**16.2**	**0.584**	**63.9**
WRN_hp1	13.7	8.71	0.672	77
WRN_hp1+5uM_gossypol	15	12.4	0.627	71.9
**WRN_hp2**	**11**	**6.89**	**0.562**	**81.5**
**WRN_hp2+5uM_gossypol**	**15.9**	**15.6**	**0.768**	**67.7**
BRCA1_hp1	18.3	11.1	1.24	69.4
BRCA1_hp1+5uM_gossypol	27.9	12.2	0.828	59.1
BRCA1_hp2	11.9	13.8	1.38	73
BRCA1_hp2+5uM_gossypol	19.2	19.4	0.627	60.8

Figure S7 After 3 days of gossypol treatment, cells were trypsinized and stained with FITCconjugated annexin V (FL1 detection, 488nm/515-545nm) and propidium iodide (FL2 detection, 488nm/564-606nm) in order to assess cell viability by flow cytometry. Percentage of dead/dying (FL1-/FL2+ and FL1+/FL2+), apoptotic (FL1+/FL2-) and living cells (FL1-/FL2-) in the presence of gossypol in the A549 cell lines silenced for the indicated candidate genes. Cells without hairpin or with a hairpin directed against luciferase, lacZ and RFP are used as negative controls. Individual shRNAs for selected genes are indicated (1 to 3 hairpins per genes). Hairpins in bold confere an increased of toxicity superior to the controls when in combination with gossypol.
