# Supplementary material for: Miniature Short Hairpin RNA Screens to Characterize Antiproliferative Drugs
Source: G3 (Bethesda). 2013 Aug 1;3(8):1375–87. doi: 10.1534/g3.113.006437 (PMC3737177; doi:10.1534/g3.113.006437)
Supplement: Supporting Information [file supp_g3.113.006437_FigureS8.pdf]

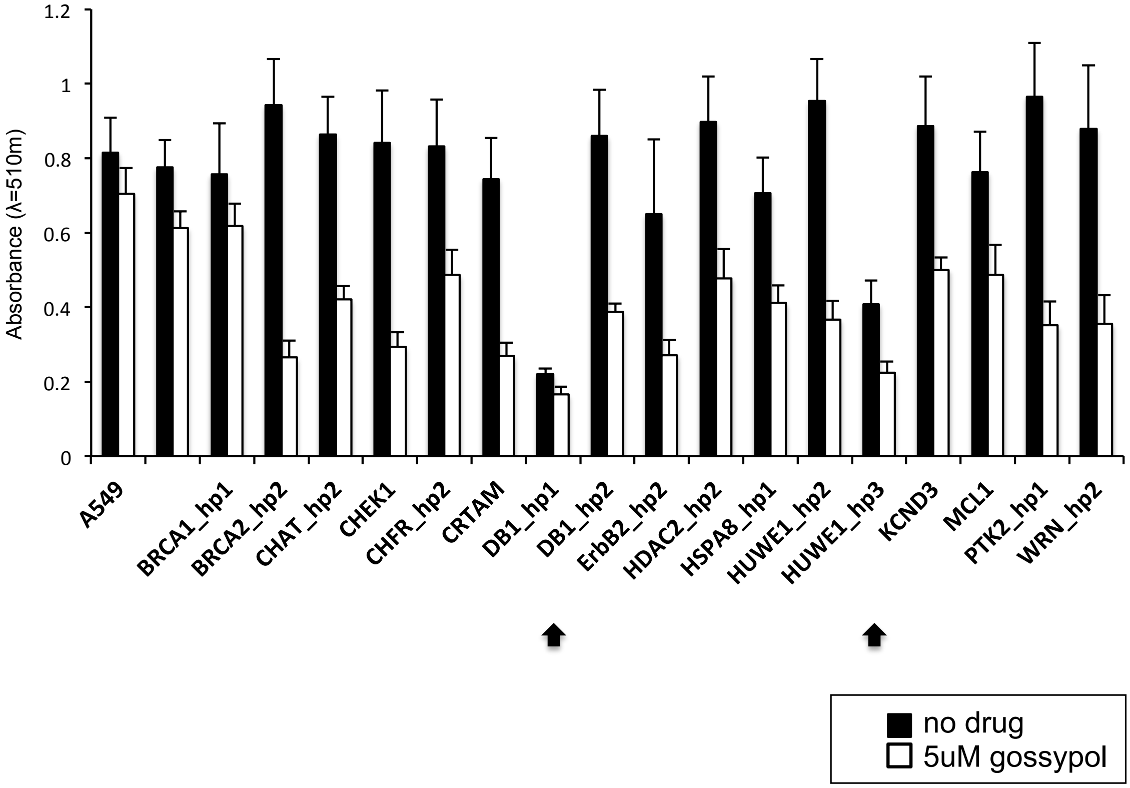

Figure S8 Effect of the hairpins targeting the gossypol potential hit genes on A549 viability. Infected A549 cells were seeded in 96-well plates at 2,200 cells/well in 200µL of medium. Gossypol was diluted first in DMSO then in RPMI, with the final DMSO concentration not exceeding 1%. After 72 hours of incubation, cell viability was measured by sulforhodamine B (SRB) viability assay. Dye concentration was determined using a microplate reader at a wavelength of 510 nm (Bio Tek Synergy 2). Intensities of the signal are proportional to the amount of stained protein and reflect the relatives cell densities. Arrows indicate toxic hairpins in the absence of drug. Errors bars show s.e.m. (n=3).
