# Supplementary material for: Miniature Short Hairpin RNA Screens to Characterize Antiproliferative Drugs
Source: G3 (Bethesda). 2013 Aug 1;3(8):1375–87. doi: 10.1534/g3.113.006437 (PMC3737177; doi:10.1534/g3.113.006437)
Supplement: Supporting Information [file supp_g3.113.006437_FigureS9.pdf]

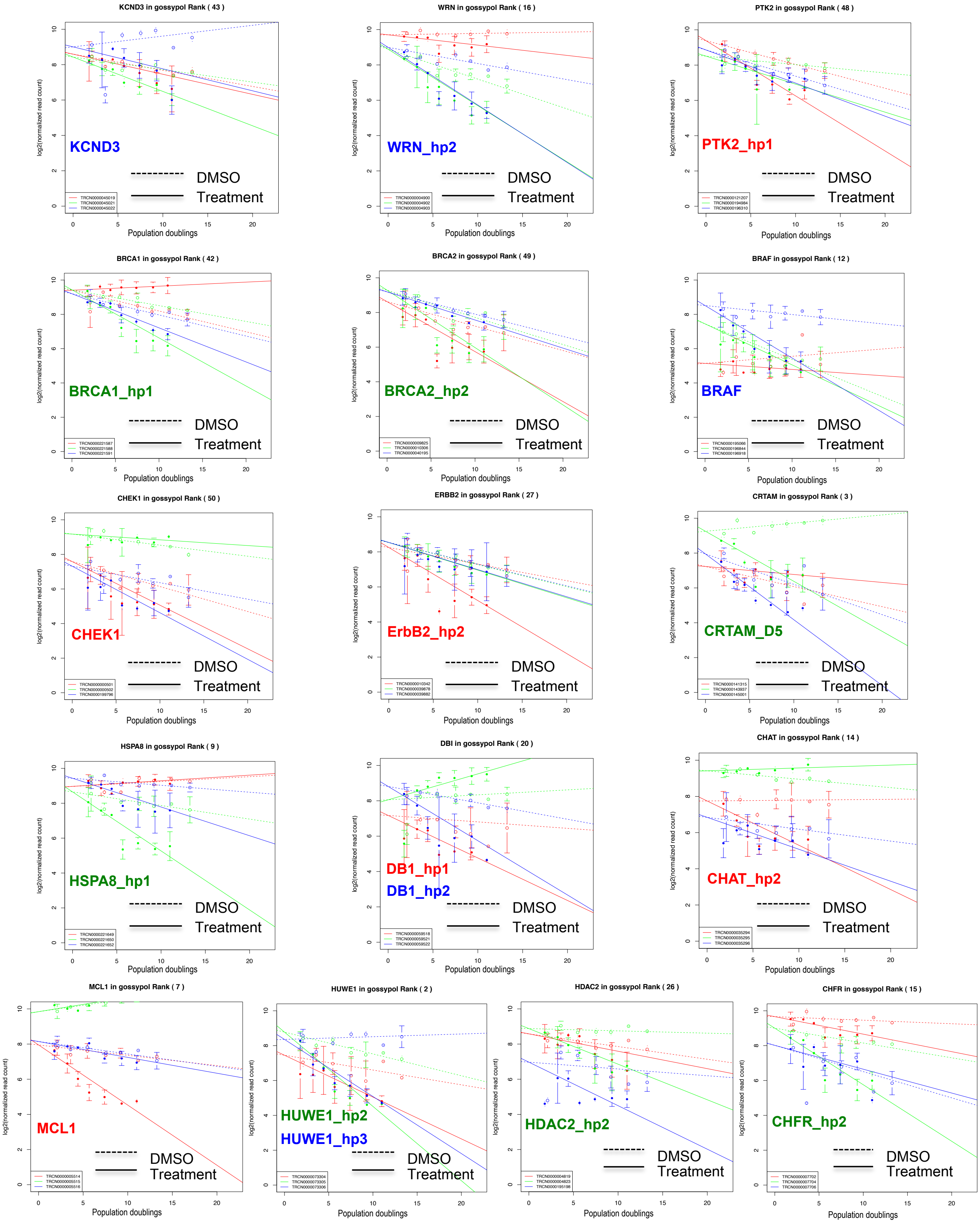

Figure S9 Potential gossypol targets are shown in sixteen linear plots. As in Figure S6, depletion of the following genes, KCND3, WRN, PTK2, BRCA1, BRCA2, BRAF, CHEK1, ERBB2, CRTAM, HSPA8, DBI, CHAT, MCL1, HUWE1, HDAC2 and CHFR confer sensitivity to gossypol treatment in A549 as shown solid line.
