# Supplementary material for: Miniature Short Hairpin RNA Screens to Characterize Antiproliferative Drugs
Source: G3 (Bethesda). 2013 Aug 1;3(8):1375–87. doi: 10.1534/g3.113.006437 (PMC3737177; doi:10.1534/g3.113.006437)
Supplement: Supporting Information [file supp_g3.113.006437_TableS1.pdf]

Class	Compound	Screening concentration (uM)	GI50	Reference	Clinical application
Antiproliferative	hydroxy urea	90	526.8uM in A549	NCI-60	antineoplastic
	etoposide	0.35	0.951uM in A549	NCI-60	antineoplastic
	camptothecin (nM)	5	19.4uM in A549	NCI-60	antineoplastic
	doxorubicin (nM)	6	0.049uM in A549	NCI-60	antineoplastic
	vincristine (nM)	6.5	10.8uM in A549	NCI-60	antineoplastic
	amsacrine	12.5	17.6uM in A549	NCI-60	antineoplastic
	methotrexate	0.03	0.023uM in A549	[1]	antineoplastic
	taxol (nM)	1	0.789uM in A549	NCI-60	antineoplastic
	gossypol	5	40uM in K562 (leukaemia), 44uM in H69 (small cell lung), 26uM in SK-mel-19 (melanoma)	[2]	antineoplastic
	methyl methanesulfonate (%)	0.0012			antineoplastic
	vorinostat	1.25	2.5 to 7.5uM in prostate cell lines LNCaP, PC-3 and TSU-Pr1	[4]	antineoplastic
	gefitinib	4	4.5uM in A549	[5]	antineoplastic
	mitomycin C	9	0.196uM in A549	NCI-60	antineoplastic
	imatinib	10	15uM in SMS-KCRN (neuroblastoma)	[6]	antineoplastic
	marimastat (BB-2516)	16	0.2uM in HT-1080/MT1 (fibrosarcoma)	[7]	antineoplastic
	digoxin (nM)	12	70nM in MDA-MB-231 (breast cancer)	[9]	heart treatment
	cyclosporin A	0.45	8.29uM in A549	NCI-60	immunosuppressive
	mycophenolic acid	0.4	41uM in A549	NCI-60	immunosuppressive
	rapamycin (nM)	1	2.93uM in A549	NCI-60	immunosuppressive
	tacrolimus	22	30uM in HEK293	[10]	immunosuppressive
	rotenone	0.1	10uM in SH-Sy5Y (neuroblastoma)	[11]	insecticide, and pesticide
	roscovitine	3	Between 13 to 36uM in various diffuse large B-cell lymphoma cell lines	[12]	treatment of non-small cell lung cancer (NSCLC), leukemia, HIV infection, herpes simplex infection
	mitaplatin	1	3.47uM in KB-3-1 (epidermoid tumor) , 6.84uM in BEL 7404 (hepatoma cell line)	[3]	antineoplastic
	retinoic acid	25	Non-small cell lung cancer: 70uM in A549, 65uM in H460, 80uM in H157	[8]	antineoplastic
Non antiproliferative	racecadotril	90			antidiarrhea
	artemisinin	22.5	16.8uM (Human Foreskin Fibroblasts)	[13]	anti-infective
	sulfasalazine	900	500uM in U87 (glioblastoma)	[14]	anti-inflammatory
	indomethacin	95	92.15uM (U138-MG glioma)	[15]	anti-inflammatory (NSAID)
	naproxen	96	1450uM in HCA-7 (colon cancer)	[16]	anti-inflammatory (NSAID)
	ibuprofen	450	900uM in HCT-116 (colon cancer)	[17]	anti-inflammatory (NSAID)
	salicylate	700	1867uM HT-29 (colon cancer)	[18]	anti-inflammatory (NSAID)
	verapamil	35	45uM Te671 (medulloblastoma)	[19]	antiarrhythmic, treatment of angina, hypertension
	tigecycline	50	3 to 8uM in leukemia cell lines	[20]	antibiotic
	erythromycin	200	712uM in CCL13 (human liver cell line)	[21]	antibiotic
	warfarin	120	184uM in mouse D3 embryonic stem cells	[22]	anticoagulant
	metformin	85	2000 to 3000uM in thyroid cancer cell lines	[23]	antihyperglycemic
	orlistat	11.2	7.5 to 21.5uM in breast cancer cell lines	[24]	antilipemic
	lovastatin	16.5	10uM in mesothelioma cell line	[25]	antilipemic
	trifluoperazine	10	Non-small cell lung cancer: 14uM in CL83, 8.5uM in CL152, 15uM in H1975	[26]	antipsychotic
	haloperidol	17.5	Prostate cancer cell lines: 177uM for LNCaP, 208uM for PC3	[27]	antipsychotic
	clozapine	16.5	250uM in hepatocytes	[28]	antipsychotic
	methimazole	400			antithyroid
	isoproterenol	27.5			asthma and bronchospasm
	aminophylline	225	428uM CEM-GH (leukemia)	[29]	asthma and bronchospasm
	propanolol	33	11.6uM in A549	NCI-60	bronchospasm and heart treatment
	mancozeb	42.5	Colon cancer cell lines: 200uM in HT-29, 80uM in Caco2	[30]	fungicide
	allopurinol	250			hyperuricemia treatment
	sildenafil	46	4.1uM in B-cell from CLL patient	[32]	pulmonary hypertension
	naltrexone	80			treatment of alcohol dependence
	MPTP	210	400uM in N2a (mouse neuroblastoma)	[31]	neurotoxin, parkinson disease

In gray: non FDA approved drugs (preclinicals, tools,…)

Table S1 Compounds used in the screens and their published GI_{50}s (drug concentrations that causes 50% cell growth inhibition). Values from the NCI-60 where retrieved from the NCI/NIH Developmental Therapeutics Program website (dtp.nci.nih.gov).
