# Supplementary material for: Miniature Short Hairpin RNA Screens to Characterize Antiproliferative Drugs
Source: G3 (Bethesda). 2013 Aug 1;3(8):1375–87. doi: 10.1534/g3.113.006437 (PMC3737177; doi:10.1534/g3.113.006437)
Supplement: Supporting Information [file supp_g3.113.006437_TableS3.pdf]

For the best experience, open this PDF portfolio in Acrobat 9 or Adobe Reader 9, or later.

Get Adobe Reader Now!
