# Supplementary material for: Miniature Short Hairpin RNA Screens to Characterize Antiproliferative Drugs
Source: G3 (Bethesda). 2013 Aug 1;3(8):1375–87. doi: 10.1534/g3.113.006437 (PMC3737177; doi:10.1534/g3.113.006437)
Supplement: Supporting Information [file supp_g3.113.006437_TableS4.pdf]

Class	Chemical	Primary hits	Reported known target	Rank #	TRC_hpID	Target Sequence	Percentage transcript remaining
Antiproliferative	etoposide	top 31	TOP1	28	TRCN0000003990	CAGAGTTGGATGGTCAGGAAT	4
			TOP2B	154	TRCN0000049284	GCAGCCTCTAATTGTGGCATT	83
			ABCB1	180	TRCN0000059683	CCGAACACATTGGAAGGAAAT	ND
		bot 29	TOP2A	1078	TRCN0000049278	GCCCAAGTGTTCTTTAGCTTT	ND
	camptothecin	top 54	TOP1	1039	TRCN0000003987	TGAAGGGCGAGTGAATCTAAG	6
				1057	TRCN0000003990	CAGAGTTGGATGGTCAGGAAT	4
		bot 15		1066	TRCN0000003991	GCTTCTCTAGTCCACCACAAA	24
	doxorubicin	top 45	TOP1	216	TRCN0000003991	GCTTCTCTAGTCCACCACAAA	24
	amsacrine	top 46	TOP1	6	TRCN0000003990	CAGAGTTGGATGGTCAGGAAT	4
				7	TRCN0000003987	TGAAGGGCGAGTGAATCTAAG	ND
			TOP2B	120	TRCN0000134040	CCAGAAACTTTCCTTGCTAAT	27
		bot 17	TOP2A	1098	TRCN0000049278	GCCCAAGTGTTCTTTAGCTTT	21
				1097	TRCN0000049279	GCCTGATTTGTCTAAGTTTAA	19
				1083	TRCN0000049280	GCTCCAAATCAATATGTGATT	21
	methotrexate	top 44	FPGS	15	TRCN0000045928	CCAGTTTGACTATGCCGTCTT	37
			FPGS	73	TRCN0000045930	TCAGACACAGTTGGAAGCCAT	68
			SLC22A6	116	TRCN0000043254	CCCTTTCTCAATGGCACAGAA	ND
			ABCC3	219	TRCN0000059406	GCACTGCTGCACAACAAGATA	ND
	taxol	top 40	ITGB3	70	TRCN0000003236	CCTTAGCCTTTGTCCCAGAAT	11
	gossypol	top 68	MCL1	7	TRCN0000005514	GCTGTGTTAAACCTCAGAGTT	ND
			BCL2L11	123	TRCN0000001051	ATGGTTATCTTACGACTGTTA	33
			BCL2	189	TRCN0000010303	TGGATGACTGAGTACCTGAAC	33
	vorinostat	top 65	HDAC9	16	TRCN0000196384	GAGCAGTTAATAGGCTTTAAA	10
			HDAC11	43	TRCN0000197158	GTTTCTGTTTGAGCGTGTGGA	17
			HDAC4	61	TRCN0000004830	CGTGGGTTTCAACGTCAACAT	57
			HDAC5	63	TRCN0000004838	GCCGGGTTTGATGCTGTTGAA	41
			HDAC2	159	TRCN0000004823	GCAAATACTATGCTGTCAATT	13
	gefitinib	top 61	RIPK2	51	TRCN0000006349	GCACCATTTCTGGATCTCAAA	92
				69	TRCN0000006347	CCAGGCTTAATTGCCCTACAA	98
			SRC	112	TRCN0000195339	CATCCTCAGGAACCAACAATT	18
	imatinib	top 27	MAPK8	8	TRCN0000194860	CAGTAAGGACTTACGTTGAAA	13
			FYN	18	TRCN0000003100	CTTACCGATCTGTCTGTCAAA	57
			FIP1L1	41	TRCN0000074422	CGAATGGGACTTGAAGTTATA	38
			ABL1	48	TRCN0000121279	CCTCAGTTCGGTGAAGGAAAT	10
				113	TRCN0000121281	ACTTGGTGAAGGTAGCTGATT	15
			ETV6	89	TRCN0000003854	GTTGTTAGTATCATGGTGTTT	12
			BCR	215	TRCN0000195722	CCCTCACTGTTGTATCTTGAA	15
	marimastat	top 34	ADAM17	109	TRCN0000052168	CCAGCAGCATTCGGTAAGAAA	23
	digoxin	top 70	SLCO4C1	170	TRCN0000038309	GCCCTGCTATTGGCTATGTAT	ND
	cyclosporinA	top 30	ABCB1	46	TRCN0000059683	CCGAACACATTGGAAGGAAAT	ND
	mycophenolic	top 18	IMPDH1	204	TRCN0000026570	CCTGAAGAAGAACCGAGACTA	ND
	rapamycin	top 27	MTOR	48	TRCN0000221544	GCTGTGCTACACTACAAACAT	ND
			ABCB1	97	TRCN0000059685	CGACAGAATAGTAACTTGTTT	ND
			FKBP3	72	TRCN0000063539	CCACTTGGTTACAGCCTATAA	2
			FKBP4	131	TRCN0000152554	GCATGGAGAAAGGAGAACATT	0
			FKBP5	225	TRCN0000000235	CGAAGGAGCAACAGTAGAAAT	5
		bot 32	FKBP1A	1,096	TRCN0000005951	GCCAAACTGACTATATCTCCA	5
				1,097	TRCN0000005953	GAGAGCCAAACTGACTATATC	15
				1,098	TRCN0000005949	AGAGAGCCAAACTGACTATAT	4
	tacrolimus	top 31	IL2RG	29	TRCN0000058470	ACGACAATTCTGACGCCCAAT	ND
			PPP3CB	138	TRCN0000002814	CCCGGAAAGAAATCATAAGAA	8
			MTOR	141	TRCN0000221543	GCAACCCTTCTTTGACAACAT	ND
			FKBP1A	153	TRCN0000005951	GCCAAACTGACTATATCTCCA	5
			IL2RA	204	TRCN0000059165	GCCACAGAGAGAATTTATCAT	ND
Non antiproliferative	racecadotril	top 53	MME	217	TRCN0000046823	CCAGGCAATTTCAGGATTATT	8
	salfasalazine	top 57	NAT2	146	TRCN0000034912	CTGGTGATGGATCCCTTACTA	ND
	ibuprofen	top 53	CXCR1	129	TRCN0000009132	GCCACTGAGATTCTGGGATTT	ND
	salicylate	top 33	SLC22A6	78	TRCN0000043253	GCAGGGATATATCCCAGGAAA	ND
	verapamil	top 62	CYP3A4	41	TRCN0000064225	CCCTGAAAGATTCAGCAAGAA	ND
			ABCB1	228	TRCN0000059684	GCAGCAATTAGAACTGTGATT	ND
	warfarin	top 41	VKORC1	2	TRCN0000038972	GCTCTCGCTCTACGCGCTGCA	7
			CYP3A4	22	TRCN0000064225	CCCTGAAAGATTCAGCAAGAA	ND
	lovastatin	top 49	CYP3A4	126	TRCN0000064225	CCCTGAAAGATTCAGCAAGAA	ND
			HMGCR	174	TRCN0000046448	GCAGTGATAAAGGAGGCATTT	79
	clozapine	top 50	CYP3A4	90	TRCN0000064225	CCCTGAAAGATTCAGCAAGAA	ND
			HRH1	119	TRCN0000011675	CCCTGGAAATTCTGCCTTATT	34
			DRD4	206	TRCN0000014453	GCCCGTACTGTGCGGCCTCAA	ND
	methimazole	top 34	TPO	138	TRCN0000045969	CGACAAGATCCTGGACTTGTA	91
	isoproterenol	top 63	ADCY10	182	TRCN0000078371	CCTGTTCAAGTATTCCATTAA	58
	aminophylline	top 48	ADORA2A	91	TRCN0000008042	CCACACCAATTCGGTTGTGAA	51
			ADORA1	145	TRCN0000008038	CCTTACCTACATTGCCATCTT	ND
	allopurinol	top 34	XDH	204	TRCN0000028098	GCAACTTTACTGTTTCAGAAA	ND

Table S4 List of potential targets for each of the 28 drugs with reported targets. In grey are the reported targets belonging to the top hits. Percentage of remaining transcript calculated by qPCR in A549, MCF7 or HEK293T/17 are also reported (source CCBR-OICR Lentiviral Technology Cancer).
