# Supplementary material for: Miniature Short Hairpin RNA Screens to Characterize Antiproliferative Drugs
Source: G3 (Bethesda). 2013 Aug 1;3(8):1375–87. doi: 10.1534/g3.113.006437 (PMC3737177; doi:10.1534/g3.113.006437)
Supplement: Supporting Information [file supp_g3.113.006437_References.pdf]

1. Rosowsky, A., et al., *Analogues of N alpha-(4-amino-4-deoxypteroyl)-N delta-hemiphthaloyl-L-ornithine (PT523) modified in the side chain: synthesis and biological evaluation.* J Med Chem, 1997. **40**(3): p. 286-99.
2. Shelley, M.D., et al., *Structure-activity studies on gossypol in tumor cell lines.* Anticancer Drugs, 2000. **11**(3): p. 209-16.
3. Xue, X., et al., *Mitaplatin increases sensitivity of tumor cells to cisplatin by inducing mitochondrial dysfunction.* Mol Pharm, 2012. **9**(3): p. 634-44.
4. Butler, L.M., et al., *Suberoylanilide hydroxamic acid, an inhibitor of histone deacetylase, suppresses the growth of prostate cancer cells in vitro and in vivo.* Cancer Res, 2000. **60**(18): p. 5165-70.
5. Van Schaeybroeck, S., et al., *Chemotherapy-induced epidermal growth factor receptor activation determines response to combined gefitinib/chemotherapy treatment in non-small cell lung cancer cells.* Mol Cancer Ther, 2006. **5**(5): p. 1154-65.
6. Beppu, K., et al., *Effect of imatinib mesylate on neuroblastoma tumorigenesis and vascular endothelial growth factor expression.* J Natl Cancer Inst, 2004. **96**(1): p. 46-55.
7. Wolf, K., et al., *Compensation mechanism in tumor cell migration: mesenchymal-amoeboid transition after blocking of pericellular proteolysis.* J Cell Biol, 2003. **160**(2): p. 267-77.
8. Soriano, A.F., et al., *Synergistic effects of new chemopreventive agents and conventional cytotoxic agents against human lung cancer cell lines.* Cancer Res, 1999. **59**(24): p. 6178-84.
9. Prassas, I. and E.P. Diamandis, *Novel therapeutic applications of cardiac glycosides.* Nat Rev Drug Discov, 2008. **7**(11): p. 926-35.
10. Lamoureux, F., et al., *Quantitative proteomic analysis of cyclosporine-induced toxicity in a human kidney cell line and comparison with tacrolimus.* J Proteomics, 2011. **75**(2): p. 677-94.
11. Kim, H.J., et al., *Naringin Protects against Rotenone-induced Apoptosis in Human Neuroblastoma SH-SY5Y Cells.* Korean J Physiol Pharmacol, 2009. **13**(4): p. 281-5.
12. Lacrima, K., et al., *Cyclin-dependent kinase inhibitor seliciclib shows in vitro activity in diffuse large B-cell lymphomas.* Leuk Lymphoma, 2007. **48**(1): p. 158-67.
13. Arav-Boger, R., et al., *Artemisinin-derived dimers have greatly improved anti-cytomegalovirus activity compared to artemisinin monomers.* PloS one, 2010. **5**(4): p. e10370.
14. Robe, P.A., et al., *In vitro and in vivo activity of the nuclear factor-kappaB inhibitor sulfasalazine in human glioblastomas.* Clin Cancer Res, 2004. **10**(16): p. 5595-603.
15. Bernardi, A., et al., *Selective cytotoxicity of indomethacin and indomethacin ethyl ester-loaded nanocapsules against glioma cell lines: an in vitro study.* Eur J Pharmacol, 2008. **586**(1-3): p. 24-34.
16. Tavolari, S., et al., *Licofelone, a dual COX/5-LOX inhibitor, induces apoptosis in HCA-7 colon cancer cells through the mitochondrial pathway independently from its ability to affect the arachidonic acid cascade.* Carcinogenesis, 2008. **29**(2): p. 371-80.
17. Khwaja, F., et al., *Ibuprofen inhibits survival of bladder cancer cells by induced expression of the p75NTR tumor suppressor protein.* Cancer Res, 2004. **64**(17): p. 6207-13.
18. Hixson, L.J., et al., *Antiproliferative effect of nonsteroidal antiinflammatory drugs against human colon cancer cells.* Cancer Epidemiol Biomarkers Prev, 1994. **3**(5): p. 433-8.
19. Schmidt, W.F., et al., *Antiproliferative effect of verapamil alone on brain tumor cells in vitro.* Cancer Res, 1988. **48**(13): p. 3617-21.
20. Skrtic, M., et al., *Inhibition of mitochondrial translation as a therapeutic strategy for human acute myeloid leukemia.* Cancer Cell, 2011. **20**(5): p. 674-88.
21. Viluksela, M., P.J. Vainio, and R.K. Tuominen, *Cytotoxicity of macrolide antibiotics in a cultured human liver cell line.* J Antimicrob Chemother, 1996. **38**(3): p. 465-73.
22. Groebe, K., et al., *Unexpected common mechanistic pathways for embryotoxicity of warfarin and lovastatin.* Reprod Toxicol, 2010. **30**(1): p. 121-30.
23. Chen, G., et al., *Metformin inhibits growth of thyroid carcinoma cells, suppresses self-renewal of derived cancer stem cells, and potentiates the effect of chemotherapeutic agents.* J Clin Endocrinol Metab, 2012. **97**(4): p. E510-20.
24. Menendez, J.A., L. Vellon, and R. Lupu, *Antitumoral actions of the anti-obesity drug orlistat (XenicalTM) in breast cancer cells: blockade of cell cycle progression, promotion of apoptotic cell death and PEA3-mediated transcriptional repression of Her2/neu (erbB-2) oncogene.* Ann Oncol, 2005. **16**(8): p. 1253-67.
25. Rubins, J.B., et al., *Lovastatin induces apoptosis in malignant mesothelioma cells.* Am J Respir Crit Care Med, 1998. **157**(5 Pt 1): p. 1616-22.
26. Yeh, C.T., et al., *Trifluoperazine, an antipsychotic agent, inhibits cancer stem cell growth and overcomes drug resistance of lung cancer.* Am J Respir Crit Care Med, 2012. **186**(11): p. 1180-8.

27. Marrazzo, A., et al., *Antiproliferative activity of phenylbutyrate ester of haloperidol metabolite II [(+/-)-MRJF4] in prostate cancer cells.* Eur J Med Chem, 2011. **46**(1): p. 433-8.
28. Li, A.P., et al., *Cryopreserved human hepatocytes: characterization of drug-metabolizing enzyme activities and applications in higher throughput screening assays for hepatotoxicity, metabolic stability, and drug-drug interaction potential.* Chem Biol Interact, 1999. **121**(1): p. 17-35.
29. Ogawa, R., et al., *Inhibition of PDE4 phosphodiesterase activity induces growth suppression, apoptosis, glucocorticoid sensitivity, p53, and p21(WAF1/CIP1) proteins in human acute lymphoblastic leukemia cells.* Blood, 2002. **99**(9): p. 3390-7.
30. Hoffman, L. and D. Hardej, *Ethylene bisdithiocarbamate pesticides cause cytotoxicity in transformed and normal human colon cells.* Environ Toxicol Pharmacol, 2012. **34**(2): p. 556-73.
31. De Girolamo, L.A., A.J. Hargreaves, and E.E. Billett, *Protection from MPTP-induced neurotoxicity in differentiating mouse N2a neuroblastoma cells.* J Neurochem, 2001. **76**(3): p. 650-60.
32. Sarfati, M., et al., *Sildenafil and vardenafil, types 5 and 6 phosphodiesterase inhibitors, induce caspase-dependent apoptosis of B-chronic lymphocytic leukemia cells.* Blood, 2003. **101**(1): p. 265-9.
